# Multi‐Technique Integration Identifies O‐GlcNAc Transferase in Fibroblast‐Like Synoviocytes as a Therapeutic Target for Rheumatoid Arthritis

**DOI:** 10.1002/advs.77466

**Published:** 2026-08-31

**Authors:** Duoli Xie, Jie Huang, Chunhao Cao, Zhenchao Tang, Xingchen Li, Jianmin Guo, Xinxin Chen, Fang Wang, Dongyi He, Jie Li, Yu Du, Bing He, Jianhua Yao, Aiping Lu, Chao Liang

**Affiliations:** ^1^ Department of Systems Biology School of Life Sciences Southern University of Science and Technology Shenzhen China; ^2^ Institute of Integrated Bioinformedicine and Translational Science (IBTS) School of Chinese Medicine Hong Kong Baptist University Hong Kong SAR China; ^3^ AI for Life Sciences Lab Tencent Shenzhen China; ^4^ Department of Rheumatology, Guanghua Hospital Affiliated to Shanghai University of Traditional Chinese Medicine Shanghai University of Traditional Chinese Medicine Shanghai China; ^5^ Department of Laboratory Medicine Peking University Shenzhen Hospital Shenzhen China; ^6^ Department of Orthopedics, The Second Affiliated Hospital of Chongqing Medical University Chongqing Medical University Chongqing China; ^7^ Shanghai University of Traditional Chinese Medicine Shanghai China; ^8^ State Key Laboratory of Proteomics National Center for Protein Sciences (Beijing) Beijing Institute of Lifeomics Beijing China

**Keywords:** artificial intelligence, fibroblast‐like synoviocytes, O‐GlcNAc transferase, PROTAC, rheumatoid arthritis

## Abstract

Rheumatoid arthritis (RA) progression is driven by the pathogenic transformation of stromal fibroblast‐like synoviocytes (FLSs). Using artificial intelligence‐guided analysis of a synovial single‐cell transcriptomic dataset, we identify O‐GlcNAc transferase (OGT) as a key regulator enriched in RA‐FLSs. Gain‐ and loss‐of‐function studies demonstrate that OGT is necessary to drive aggressive FLS phenotypes and exacerbate experimental arthritis. Mechanistically, OGT‐mediated O‐GlcNAcylation stabilizes SAP130, promoting histone deacetylation at the BTG2 promoter. This facilitates the deposition of repressive histone methylation marks, leading to BTG2 epigenetic silencing and ultimately fueling FLS aggression. To translate this mechanistic insight, we develop an FLS‐targeted proteolysis‐targeting chimera (PROTAC) by conjugating the OGT inhibitor OSMI‐1 to the AS1411 aptamer, which selectively binds surface nucleolin (NCL) overexpressed on pathogenic FLSs and, upon internalization, employs NCL as a molecular bridge to recruit the E3 ligase MDM2 for cell‐selective OGT degradation. This PROTAC suppresses FLS pathogenicity, attenuates arthritis in mice, and demonstrates additive therapeutic benefit when combined with the TNF‐α inhibitor etanercept. Collectively, our work establishes a translational paradigm that progresses from AI‐driven target discovery and mechanistic elucidation to the rational design of a cell‐type‐specific degradation therapy, offering a strategy to overcome the stromal‐driven therapeutic barrier in RA.

## Graphical abstract

AI‑guided single‑cell transcriptomics identifies OGT as a key regulator enriched in rheumatoid arthritis fibroblast‑like synoviocytes (RA‐FLSs). Inflammatory stimuli activate the OGT–SAP130 axis, which modulates the H3K27ac/H3K27me3 balance and epigenetically silences BTG2, promoting aggressive transformation of RA‑FLSs. A targeted PROTAC is developed to selectively degrade OGT in pathogenic FLSs, suppressing their aggressive phenotypes for RA therapy.

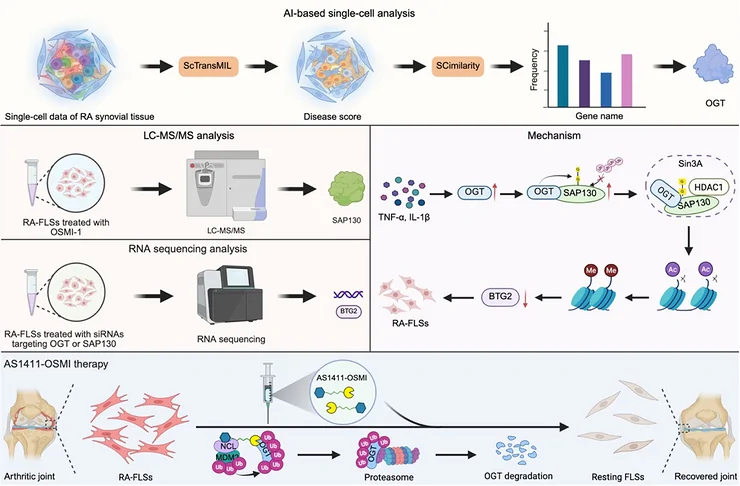

## 1 Introduction

Rheumatoid arthritis (RA) is a chronic autoimmune disorder characterized by symmetrical joint inflammation, morning stiffness, and progressive destruction of bone and cartilage, affecting approximately 0.5%–1% of adults worldwide [1, 2]. Over the past three decades, the therapeutic landscape has been revolutionized with the advent of disease‐modifying antirheumatic drugs (DMARDs), evolving from conventional synthetic agents (e.g., methotrexate and leflunomide) to biologic DMARDs such as TNF‐α inhibitors (e.g., etanercept (ETA) and adalimumab) and, more recently, to targeted synthetic DMARDs like JAK inhibitors (e.g., baricitinib and tofacitinib). Despite these advances, 25%–40% of patients still fail to achieve or sustain an adequate therapeutic response [3, 4, 5]. Moreover, even those who reach stringent clinical remission may continue to exhibit radiographic joint damage [6, 7]. This persistent structural deterioration arises largely from an autonomous, stromal‐driven disease component mediated by fibroblast‐like synoviocytes (FLSs), underscoring a fundamental limitation of current immunosuppression‐focused treatment strategies.

FLSs, key resident stromal cells within the synovium, are not merely passive responders to inflammation but active contributors to disease chronicity and joint destruction. Under persistent inflammatory conditions, FLSs undergo pathogenic transformation, acquiring an aggressive, tumor‑like phenotype marked by aberrant proliferation, resistance to apoptosis, and enhanced migratory and invasive capacity into adjacent bone and cartilage [8]. These transformed FLSs further establish a self‑reinforcing destructive cycle by secreting an array of proinflammatory cytokines and chemokines that recruit and activate immune cells, thereby perpetuating the inflammatory response. Simultaneously, they directly degrade the extracellular matrix by sustained release of matrix metalloproteinases into the joint microenvironment [9, 10]. Such functional autonomy positions FLSs as central mediators of irreversible joint damage, operating largely independently of ongoing systemic immune activity. Together, these insights establish FLSs as a critical therapeutic target, and interventions that modulate their pathogenic functions represent a promising strategy to overcome current therapeutic limitations and achieve meaningful structural repair in RA.

Single‐cell RNA sequencing (scRNA‐seq) enables systematic dissection of cellular composition and intracellular communication networks within tissues. However, the high dimensionality, sparsity, and scale of these datasets necessitate sophisticated computational methods to resolve rare, pathogenic cell states and identify their core regulatory drivers [11]. Conventional methods, which often rely on predefined markers or differential expression analyses designed for bulk RNA‐seq, are inherently biased toward abundant cell types and fail to capture context‐specific drivers of disease. Consequently, they lack the statistical power to detect rare or previously undefined populations and to elucidate the coordinated gene‑regulatory programs that drive pathology [12, 13, 14]. To overcome these limitations, we employed an artificial intelligence (AI)‐based scRNA‐seq pipeline that synergistically combines hypothesis‐free discovery with functional annotation of cell states. Our strategy employed scTransMIL, a scRNA‐seq Transformer‐based Multi‐Instance Learning framework, to identify rare cell populations associated with pathological phenotypes using only sample‐level labels across entire datasets [15]. Candidate cells were then characterized using SCimilarity, which projects them into a unified embedding space derived from a large‐scale reference atlas via a pretrained deep neural network. This step enabled data‐driven hypothesis generation and functional annotation of cell identity and potential pathological roles through quantitative similarity assessment [16]. Applying this AI‑guided framework to a public scRNA‐seq dataset [17], we identified O‐GlcNAc transferase (OGT) as a potential regulatory driver specific to pathogenic FLSs in RA.

OGT catalyzes the attachment of O‐linked N‐acetylglucosamine (O‐GlcNAc) to target proteins, thereby regulating diverse cellular processes [18]. While OGT dysfunction has been implicated in various diseases, its specific role in the aggressive transformation of FLSs in RA remains poorly understood [19]. In this study, we demonstrated its upregulation in FLSs from human RA synovium and in an arthritic mouse model. Genetic or pharmacological modulation of OGT in vitro markedly altered the aggressive phenotypes of FLSs. Critically, FLS‐specific OGT knockout in arthritic mice attenuated joint swelling, synovial hyperplasia, and bone and cartilage erosion, establishing OGT as a key pathogenic driver in RA through its action in FLSs. Mechanistically, we elucidated that OGT‑catalyzed O‑GlcNAcylation stabilized Sin3A‐associated protein 130 (SAP130), a subunit of the Sin3A/histone deacetylase (HDAC) corepressor complex [20]. This resulted in epigenetic silencing of *BTG2* [21, 22], characterized by reduced histone acetylation and increased repressive histone methylation, thereby promoting the aggressive transformation of RA‐FLSs. Based on these findings, we hypothesized that therapeutic inhibition of OGT in pathogenic FLSs could represent a viable strategy for RA treatment. However, the conventional OGT inhibitor OSMI‐1 [23] showed only modest efficacy in arthritic mice, likely due to its insufficient cell‐type specificity for pathogenic FLSs. These results underscore the pressing need to develop new therapeutic modalities that selectively target OGT in pathogenic FLSs for RA intervention.

Proteolysis‐targeting chimeras (PROTACs) are heterobifunctional molecules consisting of an E3 ligase recruiter linked to a target protein‐binding moiety, thereby directing the target protein for ubiquitination and proteasomal degradation [24]. Building on this concept, we previously developed a cell‐selective PROTAC platform centered on the AS1411 aptamer targeting nucleolin (NCL) [25]. Unlike conventional PROTACs, our AS1411‑based system preferentially degrades proteins in disease‐relevant cells that overexpress NCL on their surface [25]. Mechanistically, AS1411 recognizes surface‐exposed NCL, facilitating selective internalization. Once internalized, AS1411 exploits NCL as a molecular bridge to recruit the E3 ligase murine double minute 2 (MDM2), resulting in cell‑type‑restricted degradation of the target protein [25]. Notably, our recent studies revealed that pathogenic FLSs in RA also exhibit high surface NCL expression [26]. This led us to hypothesize that conjugating OSMI‑1 to AS1411 could yield a PROTAC that selectively degrades OGT in pathogenic FLSs, thereby overcoming the lack of cellular specificity associated with conventional OSMI‑1. Supporting this hypothesis, we found that AS1411 accumulated in the inflamed synovium of arthritic mice, and the AS1411‑OSMI conjugate selectively degraded OGT in pathogenic FLSs and suppressed their aggressive phenotypes in vitro. Systemic administration of this PROTAC alleviated disease severity in arthritic mouse models. Combination therapy with this PROTAC and ETA demonstrated additive therapeutic benefit, supporting the translational potential of this targeted protein degradation strategy for RA treatment.

In summary, this work establishes a novel translational paradigm that navigates from the AI‐guided identification of OGT as a pathogenic driver in FLSs, through the mechanistic dissection of the OGT‑SAP130‑BTG2 signaling axis, to the rational design of a targeted PROTAC for RA treatment. This integrated framework provides a precise strategy to intervene in stromal‐driven pathology, thereby addressing a central therapeutic bottleneck in RA.

## 2 Results

### 2.1 OGT is Identified as a Key Disease‐Associated Driver in RA

**FIGURE 1:**
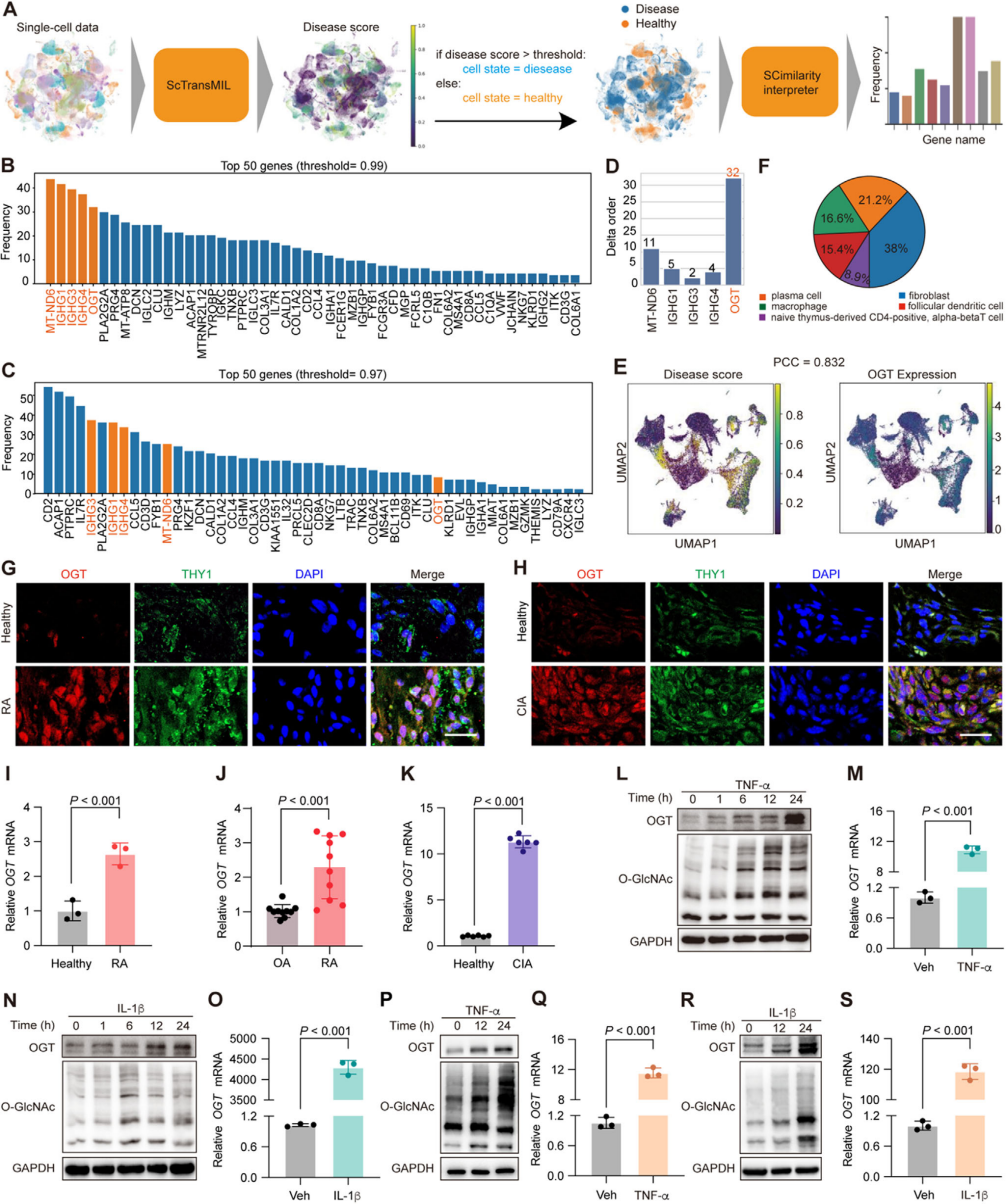
Identification of OGT as a key disease‐associated driver in RA. (A) AI‐based scRNA‐seq analysis flowchart for RA‐related cells and genes. Briefly, scRNA‐seq data were analyzed using the scTransMIL model to calculate disease scores at cellular resolution. Cells are classified as diseased/healthy based on a defined threshold, and the SCimilarity interpreter generates a gene frequency plot in which high‐frequency genes represent strong disease associations. n = 79. (B and C) Frequency plots showing the top 50 genes identified at a threshold of 0.99 (B) or 0.97 (C). n = 79. (D) Delta order of top 5 genes when disease score threshold decreases from 0.99 to 0.97. n = 79. (E) UMAP visualization showing a correlation between disease score and *OGT* expression. n = 79. (F) Cell‐type landscape by SCimilarity annotation after retaining every disease cell at the 0.99 threshold. n = 79. (G) IF staining of synovial tissues from healthy controls and RA patients using anti‐OGT and anti‐THY1 antibodies. THY1 served as a fibroblastic marker. n = 3. Scale bars, 25 µm. (H) IF staining of synovial tissues from healthy controls and CIA mice using anti‐OGT and anti‐THY1 antibodies. n = 5. Scale bars, 25 µm. (I) *OGT* mRNA levels in synovial tissues from healthy controls and RA patients. n = 3. (J) *OGT* mRNA levels in synovial tissues from OA and RA patients. n = 10. (K) *OGT* mRNA levels in synovial tissues from healthy controls and CIA mice. n = 6. (L) OGT and O‐GlcNAcylation levels in RA‐FLSs upon stimulation with 10 ng/mL TNF‐α at the indicated time points. n = 3. (M) *OGT* mRNA levels in RA‐FLSs upon stimulation with 10 ng/mL TNF‐α for 24 h. n = 3. (N) OGT and O‐GlcNAcylation levels in RA‐FLSs upon stimulation with 10 ng/mL IL‐1β at the indicated time points. n = 3. (O) *OGT* mRNA levels in RA‐FLSs upon stimulation with 10 ng/mL IL‐1β for 24 h. n = 3. (P) OGT and O‐GlcNAcylation levels in mouse FLSs upon stimulation with 10 ng/mL TNF‐α at the indicated time points. n = 3. (Q) *OGT* mRNA levels in mouse FLSs upon stimulation with 10 ng/mL TNF‐α for 24 h. n = 3. (R) OGT and O‐GlcNAcylation levels in mouse FLSs upon stimulation with 10 ng/mL IL‐1β at the indicated time points. n = 3. (S) *OGT* mRNA levels in mouse FLSs upon stimulation with 10 ng/mL IL‐1β for 24 h. n = 3. Data are presented as mean ± SD, and statistical significance is calculated by the Mann‐Whitney U test (I and J) and unpaired *t*‐test (K, M, O, Q, and S).

We determined the reliability of the scTransMIL model using a public scRNA‐seq dataset of synovial tissues from RA and healthy individuals [17] by examining the inferred single‐cell disease scores, which exhibited a clear bimodal distribution and indicated high‐confidence classification (Figure S1A). Then, we imported the scRNA‐seq data into the model and calculated disease scores at cellular resolution. Cells were assigned to diseased or healthy states using predefined thresholds, and the SCimilarity interpreter generated gene‐frequency plots in which high‐frequency genes represent strong disease associations (Figure 1A). At a threshold of 0.99 the top five genes were mitochondrially encoded NADH dehydrogenase 6 (MT‐ND6), immunoglobulin heavy constant gamma 1 (IGHG1), IGHG3, IGHG4, and OGT (Figure 1B). When the threshold was lowered to 0.97, these genes were no longer all among the top five (Figure 1C). Comparing the two rankings, we computed a delta order and found that OGT displayed the largest rank change, identifying it as the most threshold‐sensitive gene and suggesting a strong association with RA (Figure 1D). Expression patterns showed that OGT levels mirrored disease scores, corroborating their biological relevance to RA (Figure 1E). To identify the corresponding cell types, we performed SCimilarity‐based annotation and retained only those cells captured by the stringent 0.99 threshold (Figure S1B). Among the five most abundant cell types in this disease‐enriched pool, fibroblasts constitute the largest fraction (Figure 1F and Figure S1C). Visualization of fibroblast markers, including thy‐1 cell surface antigen (*THY1*), platelet‐derived growth factor receptor alpha (*PDGFRA*), c‐x‐c motif chemokine ligand 12 (*CXCL12*), chloride intracellular channel 5 (*CLIC5*), *NCL*, proteoglycan 4 (*PRG4*), podoplanin (*PDPN*), gamma‐glutamyltransferase 5 (*GGT5*), and dickkopf‐related protein 3 (*DKK3*), confirmed their selective enrichment within the RA‐associated fraction (Figure S1D). Together, these analyses revealed that OGT was a gene specifically elevated in disease‐associated fibroblasts, implicating it as a potential driver of pathogenic FLS states in RA.

To establish the relevance of OGT with FLSs in RA, we assessed its expression in human synovial tissues. immunofluorescence (IF) staining revealed that OGT levels were markedly upregulated in these tissues from RA patients compared with those from healthy controls. Importantly, we observed significant enrichment of OGT within THY1‐positive FLSs (Figure 1G). We next examined synovial tissues from collagen‐induced arthritis (CIA) mice. Consistent with the human data, OGT expression was substantially elevated in CIA synovium (Figure 1H). Quantitative reverse transcription polymerase chain reaction (qRT‐PCR) analysis confirmed OGT upregulation at the transcriptional level in human RA synovial tissues compared to healthy or osteoarthritis (OA) controls (Figure 1I,J), as well as in synovial tissues from CIA mice compared to healthy controls (Figure 1K). In RA, immune cells infiltrate the synovium and secrete proinflammatory cytokines, which drive the pathological activation of FLSs [27]. To investigate whether OGT expression in RA‐FLSs responds to cytokine stimulation such as TNF‐α and IL‐1β, we isolated primary RA‐FLSs. These RA‐FLSs expressed high levels of fibroblastic markers THY1 and vimentin [28, 29], but not the macrophage marker CD68 [30], confirming their identity (Figure S2A). Western blot and qRT‐PCR analyses revealed that TNF‐α stimulation significantly increased OGT protein and mRNA levels (Figure 1L,M). Similarly, IL‐1β treatment upregulated OGT expression in RA‐FLSs (Figure 1N,O). We also isolated and characterized primary mouse FLSs using THY1 and vimentin (Figure S2B). The mouse FLSs showed enhanced OGT expression upon TNF‐α or IL‐1β stimulation (Figure 1P–S). Given that OGT is the sole enzyme catalyzing O‐GlcNAcylation, we investigated whether this upregulation resulted in a functional increase in protein O‐GlcNAcylation. As hypothesized, both TNF‐α and IL‐1β markedly enhanced global O‐GlcNAcylation levels in RA‐FLSs and mouse FLSs (Figure 1L,N,P,R).

### 2.2 OGT Promotes the Aggressive Transformation of RA‐FLSs In Vitro

**FIGURE 2:**
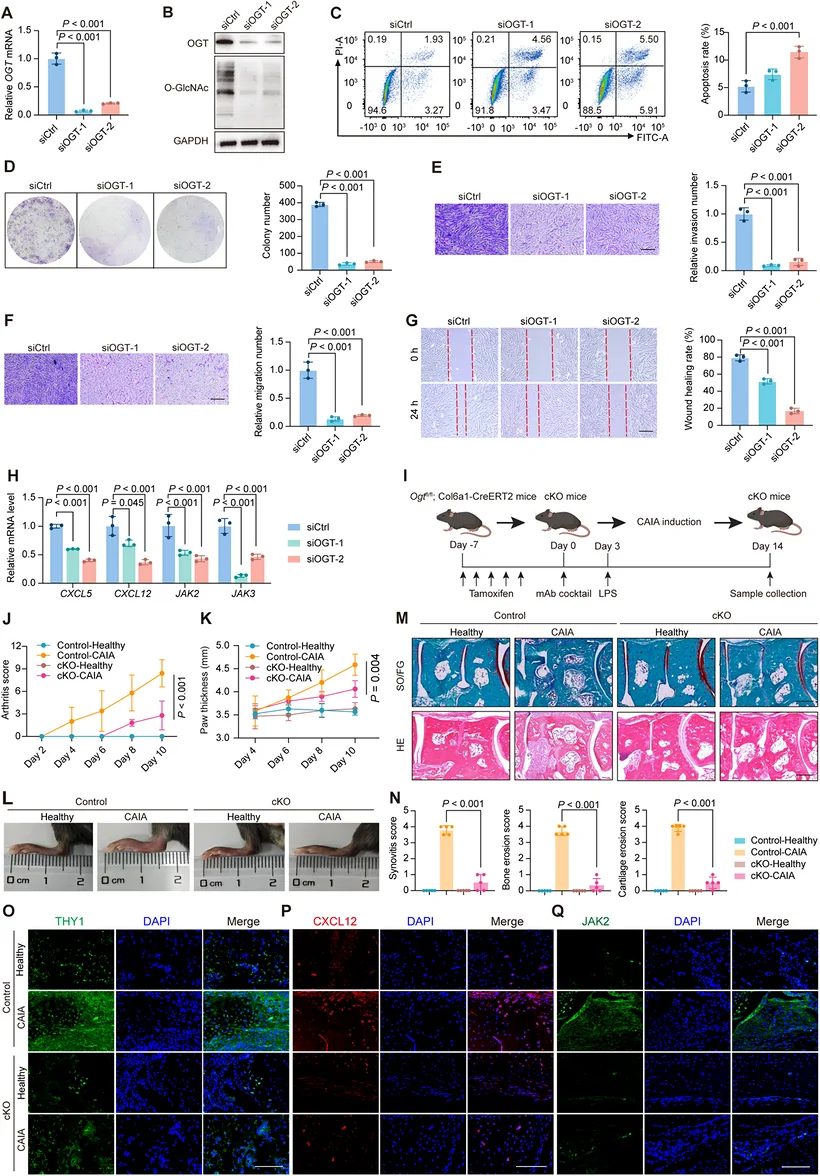
Pathogenic role of OGT in aggressive transformation of RA‐FLSs and CAIA mouse models. (A) *OGT* mRNA levels in RA‐FLSs after transfection with siCtrl, siOGT‐1, or siOGT‐2. n = 3. (B) OGT and O‐GlcNAcylation levels in RA‐FLSs after transfection with siCtrl, siOGT‐1, or siOGT‐2. n = 3. (C) Apoptosis of RA‐FLSs transfected with siCtrl, siOGT‐1, or siOGT‐2, as determined by flow cytometry. Quantification is shown on the right. n = 3. (D) Colony formation of RA‐FLSs transfected with siCtrl, siOGT‐1, or siOGT‐2. Quantification is shown on the right. n = 3. (E) Transwell invasion of RA‐FLSs transfected with siCtrl, siOGT‐1, or siOGT‐2. Quantification is shown on the right. n = 3. Scale bars, 50 µm. (F) Transwell migration of RA‐FLSs transfected with siCtrl, siOGT‐1, or siOGT‐2. Quantification is shown on the right. n = 3. Scale bars, 50 µm. (G) Wound healing of RA‐FLSs transfected with siCtrl, siOGT‐1, or siOGT‐2. Quantification is shown on the right. n = 3. Scale bars, 50 µm. (H) mRNA expression of *CXCL12*, *CXCL5*, *JAK2*, and *JAK3* in RA‐FLSs transfected with siCtrl, siOGT‐1, or siOGT‐2. n = 3. (I) Schematic diagram for establishing the CAIA mouse model using control and FLS‐specific OGT cKO mice. Tamoxifen and corn oil were intraperitoneally administered to *Ogt* ^fl/fl^; Col6a1‐CreERT2 mice to generate FLS‐specific OGT cKO mice and control mice, respectively. Then, both control and OGT cKO mice received a tail vein injection of the type II collagen (CII) mAb cocktail on day 0, followed by intraperitoneal injection of LPS on day 3 to trigger arthritis. (J and K) Arthritis score (J) and hind paw thickness (K) of control and cKO mice with or without CAIA induction. Mice without CAIA were designated as the healthy group. n = 5. (L) Representative hind paw images of control mice and cKO mice with or without CAIA induction. n = 5. (M) SO/FG and HE staining of paw sections of control mice and cKO mice with or without CAIA induction. n = 5. Scale bar, 200 µm. (N) Quantitative scoring of synovitis, bone, and cartilage erosion on SO/FG‐ and HE‐ stained sections of control mice and cKO mice with or without CAIA induction. n = 5. (O‐Q) IF staining of paw sections of control mice and cKO mice with or without CAIA induction, using anti‐THY1 (O), anti‐CXCL12 (P), or anti‐JAK2 (Q) antibodies. n = 5. Scale bar, 100 µm. Data are represented as mean ± SD, and statistical significance is calculated by one‐way ANOVA with Dunnett's test (A and C‐H) or Tukey's test (N) and two‐way ANOVA (J and K).

To demonstrate the functional significance of OGT in RA, we conducted a series of gain‐ and loss‐of‐function experiments in RA‐FLSs in vitro. First, OGT was silenced using RNA interference, which effectively reduced both OGT mRNA and protein levels and led to a corresponding decrease in global O‑GlcNAcylation (Figure 2A,B). Flow cytometric analysis showed that silencing of OGT significantly promoted the apoptosis of RA‐FLSs (Figure 2C). Colony formation assay indicated that OGT knockdown reduced the proliferation of RA‐FLSs (Figure 2D). Transwell and wound healing assays demonstrated that silencing of OGT markedly impaired the invasive and migratory capacities of RA‐FLSs (Figure 2E–G). Additionally, OGT silencing downregulated mRNA levels of proinflammatory mediators, including *CXCL5*, *CXCL12*, *janus kinase 2* (*JAK2)*, and *JAK3* (Figure 2H). Then, we overexpressed OGT in RA‐FLSs (Figure S3A,B). TUNEL assay revealed that OGT overexpression inhibited apoptosis (Figure S3C), while EdU assay demonstrated that overexpressed OGT promoted proliferation of RA‐FLSs (Figure S3D). Transwell and wound healing assays showed that overexpressed OGT enhanced the invasive and migratory abilities of RA‐FLSs (Figure S3E–G). The proinflammatory mediators, including *CXCL5*, *CXCL12*, *JAK2*, and *JAK3*, were upregulated by OGT overexpression (Figure S3H). Complementing the genetic approach, pharmacological inhibition of OGT with OSMI‐1 effectively reduced O‐GlcNAcylation without affecting OGT mRNA and protein expression (Figure S3I,J). OSMI‐1 treatment promoted apoptosis, suppressed proliferation, invasion, and migration, and reduced mRNA expression of inflammatory mediators (Figure S3K–P). Together, these results demonstrate that OGT promotes aggressive transformation of RA‑FLSs in vitro.

### 2.3 FLS‐Specific Deletion of OGT Attenuates the Progression of Experimental Arthritis

To investigate the functional role of OGT in FLSs in vivo, we generated mice harboring floxed *Ogt* alleles and the Col6a1‐CreERT2 transgene (*Ogt^fl/fl^*; Col6a1‐CreERT2) (Figure S4A,B). Then, *Ogt^fl/fl^*; Col6a1‐CreERT2 mice received an injection of tamoxifen (TAM) to induce conditional *Ogt* knockout (cKO) deletion in FLSs, as Col6a1 is predominantly expressed in FLSs [31, 32, 33, 34]. In contrast, *Ogt^fl/fl^*; Col6a1‐CreERT2 mice injected with corn oil were used as controls (Figure 2I). Western blot and IF analysis confirmed that TAM administration successfully induced *Ogt* knockout in the synovial tissues of mice (Figure S4C,D). However, it has been reported that Col6a1 is also expressed in other mesenchymal populations within the joint microenvironment [35, 36]. Therefore, it was important to verify the specificity of *Ogt* deletion. To address this, we examined OGT protein levels in other mesenchymal populations of adjacent joint tissues, specifically osteoblasts and chondrocytes, from cKO and control mice. Our results demonstrated that OGT expression in these cell types was minimally reduced in cKO mice (Figure S4E,F). This preservation of OGT expression in osteoblasts and chondrocytes is reasonably attributed to the markedly lower Col6a1 expression in these cell types compared to that in FLSs (Figure S4G), ensuring that Cre‑mediated recombination occurs predominantly in FLSs. These findings are consistent with previous reports showing that Col6a1‑Cre transgenic mouse models are primarily used to study FLSs in arthritis and are less commonly employed for targeted deletion in osteoblasts or chondrocytes [31, 37]. To investigate the effect of FLS‐specific OGT knockout on arthritis progression, we employed the collagen antibody‐induced arthritis (CAIA) model (Figure 2I). Clinical assessment revealed that cKO mice exhibited significantly attenuated arthritis scores and reduced paw swelling compared to control mice (Figure 2J–L). Histopathological analysis of paw sections using Safranin O/Fast Green (SO/FG) and hematoxylin and eosin (HE) staining confirmed that synovitis, bone erosion, and cartilage destruction were markedly ameliorated in cKO mice (Figure 2M,N). IF analysis showed a significant downregulation of key pathogenic markers, including THY1, CXCL12, and JAK2, in the synovial tissues of cKO mice compared to controls (Figure 2O–Q). Collectively, these findings demonstrate that genetic ablation of OGT specifically in FLSs effectively suppresses experimental arthritis.

### 2.4 SAP130 is Identified as a Direct O‐GlcNAc Substrate of OGT in RA‐FLSs

**FIGURE 3:**
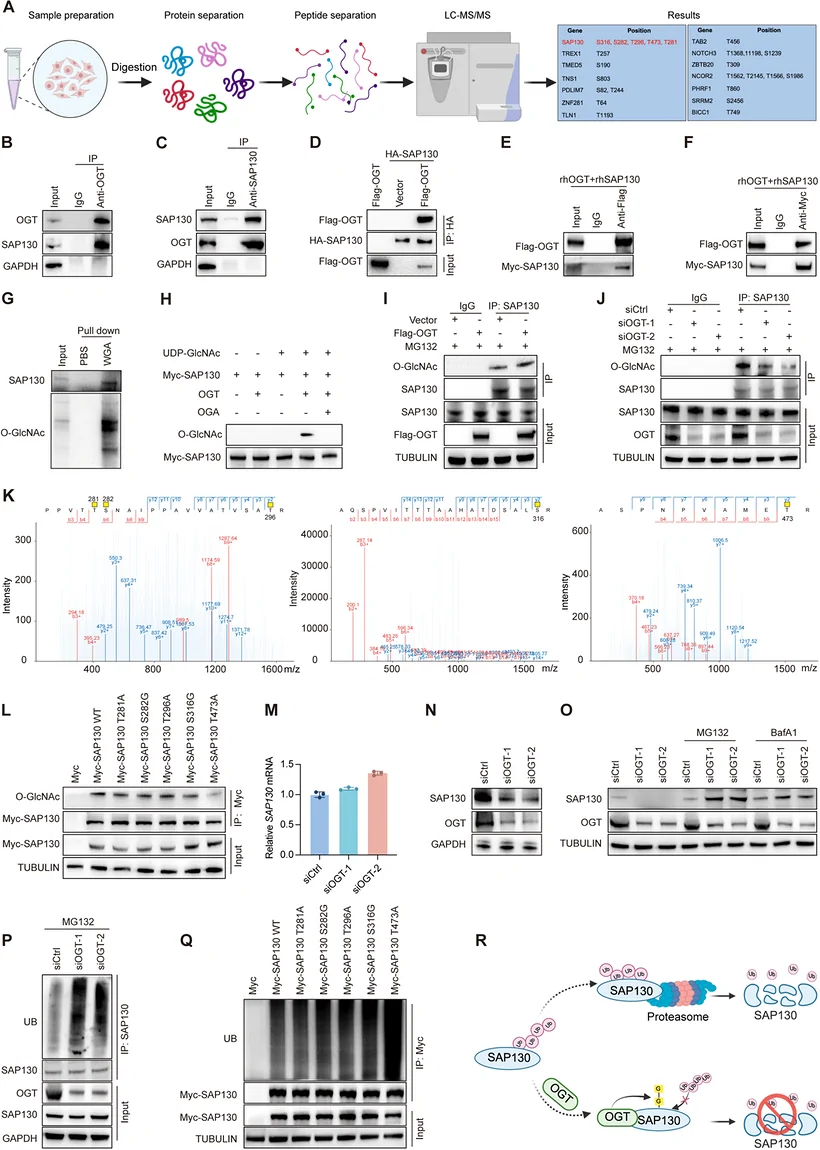
Stabilization of SAP130 by OGT‐mediated O‐GlcNAcylation. (A) Workflow of LC‐MS/MS‐based analysis for identifying OGT‐interacting proteins and their potential O‐GlcNAcylation sites. Briefly, RA‐FLSs were lysed, followed by protein digestion, peptide separation, and LC‐MS/MS detection. The right panel summarizes the identified protein candidates and their potential O‐GlcNAcylation modification sites obtained via this workflow. (B and C) Co‐IP assays for the interaction between OGT and SAP130 in RA‐FLSs using an anti‐OGT antibody (B) and an anti‐SAP130 antibody (C), respectively. n = 3. (D) Co‐IP assay for the interaction between OGT and SAP130 in 293T cells overexpressing HA‐tagged SAP130 (HA‐SAP130), after transfection with the Flag‐empty vector or the vector expressing Flag‐tagged OGT (Flag‐OGT). n = 3. (E and F) Co‐IP assays for the interaction between OGT and SAP130 in vitro using an anti‐OGT antibody (E) and an anti‐SAP130 antibody (F), respectively. 2 µg/ml Flag‐tagged rhOGT was incubated with 2 µg/ml Myc‐tagged rhSAP130 for 7 h before the co‐IP assay. 15% of the rhOGT‐rhSAP130 mixture was used as input. n = 3. (G) WGA pull‐down assay for determining O‐GlcNAcylation levels of SAP130 in RA‐FLSs. n = 3. (H) In vitro O‐GlcNAcylation assays with Myc‐SAP130 as a substrate, in the presence of UDP‐GlcNAc, OGT, or OGA. n = 3. (I) O‐GlcNAcylation levels of SAP130 in RA‐FLSs transfected with the Flag‐empty vector or the vector expressing Flag‐OGT, in the presence of 5 µM proteasome inhibitor MG132. n = 3. (J) O‐GlcNAcylation levels of SAP130 in RA‐FLSs transfected with siCtrl, siOGT‐1, or siOGT‐2, in the presence of 5 µM MG132. n = 3. (K) Potential O‐GlcNAcylation modification sites (T281, S282, T296, S316, and T473) on SAP130 identified via LC‐MS/MS analysis. (L) Co‐IP assay using anti‐Myc antibody beads to assess O‐GlcNAcylation levels of overexpressed Myc‐SAP130 WT and its mutants (T281A, S282A, T296A, S316G, or T473A) in 293T cells. n = 3. (M and N) mRNA (M) and protein (N) levels of SAP130 in RA‐FLSs transfected with siCtrl, siOGT‐1, or siOGT‐2. n = 3. (O) SAP130 protein levels in RA‐FLSs transfected with siCtrl, siOGT‐1, or siOGT‐2, in the presence of 5 µM MG132 or 400 nM BafA1. n = 3. (P) Ubiquitination levels of SAP130 in RA‐FLSs transfected with siCtrl, siOGT‐1, or siOGT‐2, in the presence of 5 µM MG132. n = 3. (Q) Ubiquitination levels of overexpressed Myc‐SAP130 WT and its mutants (T281A, S282A, T296A, S316G, or T473A) in 293T cells. n = 3. (R) Schematic model illustrating that OGT‐mediated O‐GlcNAcylation stabilizes SAP130 by inhibiting its ubiquitination and proteasomal degradation. Data are presented as mean ± SD, and statistical significance is calculated by one‐way ANOVA with Dunnett's test (M).

We conducted liquid chromatography‐tandem mass spectrometry (LC‐MS/MS)‐based O‐GlcNAc proteomic profiling of RA‐FLSs treated with DMSO or OSMI‐1 (Figure 3A). Comparative analysis identified several candidate proteins with altered O‐GlcNAcylation upon OSMI‐1 treatment. Among these proteins, SAP130 emerged as a top candidate, showing a marked reduction in O‐GlcNAcylation levels at five potential residues, including T281, S282, T296, S316, and T473 (Figure 3A). Co‐immunoprecipitation (co‐IP) assays confirmed an interaction between endogenous OGT and SAP130 in RA‐FLSs (Figure 3B,C). This interaction was validated by ectopic co‐expression of Flag‐tagged OGT and HA‐tagged SAP130 in 293T cells (Figure 3D) and by direct binding assays using purified recombinant human OGT (rhOGT) and rhSAP130 (Figure 3E,F), demonstrating a physical association between the two proteins. We next confirmed that SAP130 is an authentic O‐GlcNAcylated substrate. Wheat germ agglutinin (WGA) pull‐down assays successfully enriched SAP130 from RA‐FLS lysates (Figure 3G). We showed that OGT directly O‐GlcNAcylated SAP130 in vitro in the presence of UDP‐GlcNAc, the substrate for O‐GlcNAcylation (Figure 3H). Conversely, O‐GlcNAcase (OGA), which removes O‐GlcNAc moieties [38], reversed the O‐GlcNAcylation of SAP130 (Figure 3H). Consistent with these findings, OGT overexpression in RA‐FLSs increased SAP130 O‐GlcNAcylation (Figure 3I), whereas OGT knockdown decreased it (Figure 3J). Since LC‐MS/MS had mapped five potential O‐GlcNAcylation sites on SAP130 (Figure 3K), we mutated each residue by substituting threonine with alanine (T→A) or serine with glycine (S→G). Among the single‐point mutants (T281A, S282G, T296A, S316G, and T473A), only T473A significantly abolished SAP130 O‐GlcNAcylation (Figure 3L), identifying T473 as the primary site targeted by OGT.

### 2.5 OGT‐Mediated O‐GlcNAcylation Stabilizes SAP130 by Antagonizing Its Proteasomal Degradation

To investigate whether OGT regulates SAP130 expression, we measured SAP130 levels following OGT silencing in RA‐FLSs. While SAP130 mRNA remained unchanged after OGT knockdown (Figure 3M), its protein expression was markedly decreased (Figure 3N). Given that O‐GlcNAcylation often protects proteins from ubiquitin‑proteasomal degradation [39, 40, 41], we tested whether this pathway mediates SAP130 destabilization upon OGT loss. We demonstrated that the proteasomal inhibitor MG132 largely restored SAP130 protein levels in OGT‐silenced RA‐FLSs, whereas the lysosomal inhibitor Bafilomycin A1 (BafA1) showed minimal effect (Figure 3O). Consistently, ubiquitination of SAP130 increased upon OGT knockdown (Figure 3P), indicating that OGT mainly stabilizes SAP130 by counteracting the ubiquitin‑proteasomal pathway. We further compared ubiquitination of wild‑type (WT) SAP130 and its point mutants at potential O‑GlcNAcylation sites (T281A, S282G, T296A, S316G, and T473A). The T473A mutant displayed significantly higher ubiquitination than WT (Figure 3Q), demonstrating that O‑GlcNAcylation at T473 inhibits SAP130 ubiquitination. Together, these findings establish that OGT stabilizes SAP130 by catalyzing O‑GlcNAcylation at T473, thereby blocking ubiquitination and protecting SAP130 from proteasomal degradation (Figure 3R).

### 2.6 SAP130 Functions as a Key Mediator of the OGT‐Driven Aggressive Transformation of RA‐FLSs

**FIGURE 4:**
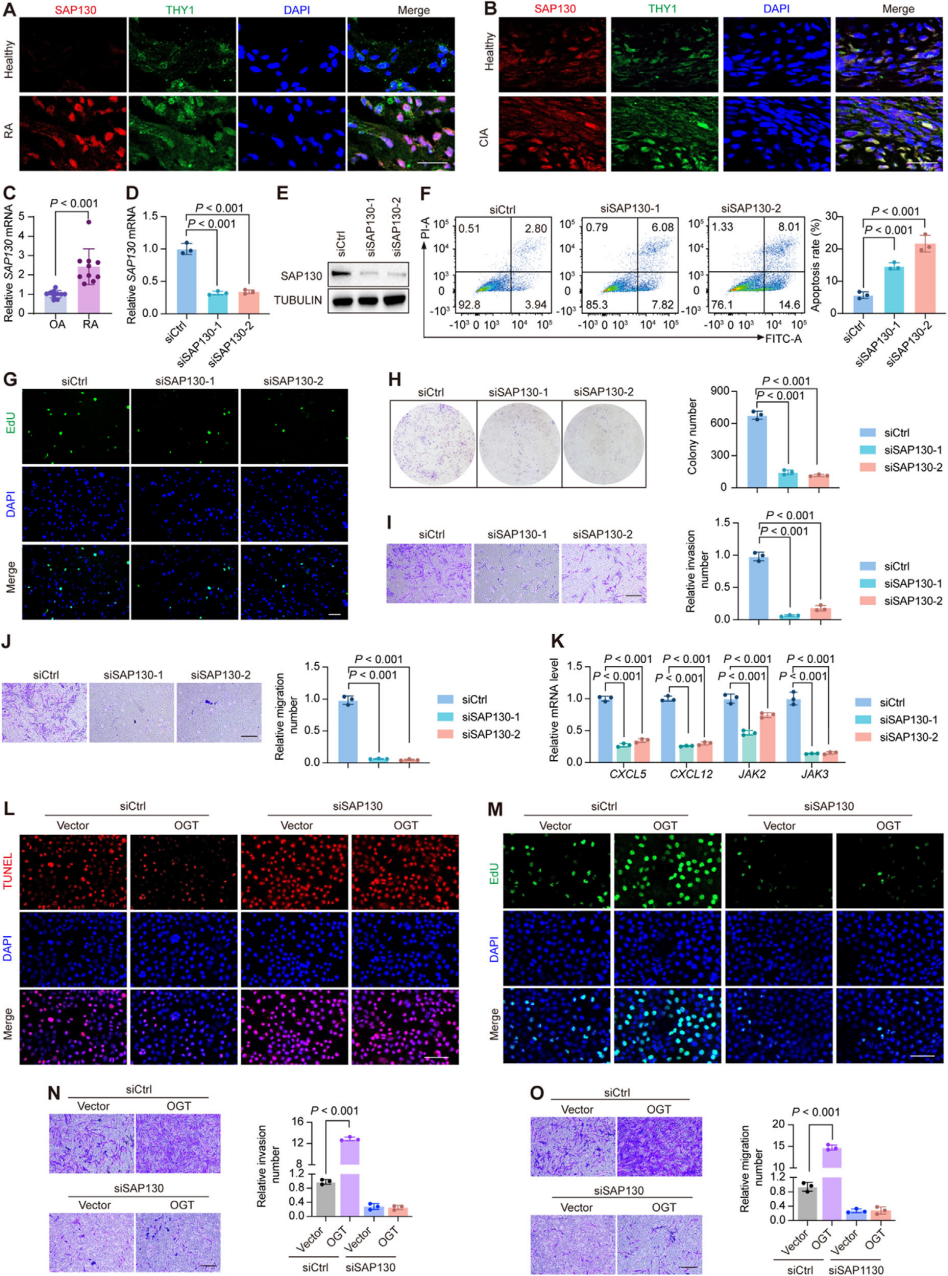
SAP130‐dependent aggressive transformation of RA‐FLSs driven by OGT. (A) IF staining of synovial tissues from healthy controls and RA patients using anti‐SAP130 and anti‐THY1 antibodies. n = 3. Scale bars, 25 µm. (B) IF staining of synovial tissues from healthy controls and CIA mice using anti‐SAP130 and anti‐THY1 antibodies. n = 5. Scale bars, 25 µm. (C) *SAP130* mRNA levels in synovial tissues from OA and RA patients. n = 10. (D and E) mRNA (D) and protein (E) levels of SAP130 in RA‐FLSs transfected with siCtrl, siSAP130‐1, or siSAP130‐2. n = 3. (F) Apoptosis of RA‐FLSs transfected with siCtrl, siSAP130‐1, or siSAP130‐2, as determined by flow cytometry. Quantification is shown on the right. n = 3. (G) Proliferation of RA‐FLSs transfected with siCtrl, siSAP130‐1, or siSAP130‐2, as measured by EdU staining. n = 3. Scale bars = 100 µm. (H) Colony formation of RA‐FLSs transfected with siCtrl, siSAP130‐1, or siSAP130‐2. Quantification is shown on the right. n = 3. (I) Transwell invasion of RA‐FLSs transfected with siCtrl, siSAP130‐1, or siSAP130‐2. Quantification is shown on the right. n = 3. Scale bars, 50 µm. (J) Transwell migration of RA‐FLSs transfected with siCtrl, siSAP130‐1, or siSAP130‐2. Quantification is shown on the right. n = 3. Scale bars, 50 µm. (K) mRNA expression of *CXCL5*, *CXCL12*, *JAK2*, and *JAK3* in RA‐FLSs transfected with siCtrl, siSAP130‐1, or siSAP130‐2. n = 3. (L) Apoptosis of SAP130‐silenced RA‐FLSs after transfection with the empty vector or the OGT‐overexpressing vector, as measured by TUNEL staining. n = 3. Scale bars, 100 µm. (M) Proliferation of SAP130‐silenced RA‐FLSs after transfection with the empty vector or the OGT‐overexpressing vector, as measured by EdU staining. n = 3. Scale bars, 100 µm. (N) Transwell invasion of SAP130‐silenced RA‐FLSs after transfection with the empty vector or the OGT‐overexpressing vector. Quantification is shown on the right. n = 3. Scale bars, 50 µm. (O) Transwell migration of SAP130‐silenced RA‐FLSs after transfection with the empty vector or the OGT‐overexpressing vector. Quantification is shown on the right. n = 3. Scale bars, 50 µm. Data are presented as mean ± SD, and statistical significance is calculated by the Mann‐Whitney U test (C), one‐way ANOVA with Dunnett's test (D, F, and H‐K), and Tukey's test (N and O).

IF analysis revealed that SAP130 levels, mirroring the pattern of OGT, were significantly elevated in synovial tissues from RA patients and CIA mice compared to healthy controls (Figure 4A,B). qRT‐PCR analysis showed higher OGT mRNA levels in human RA synovial tissues than in OA controls (Figure 4C). We investigated the functional impact of SAP130 on the aggressive phenotypes of RA‐FLSs. We observed that efficient knockdown of SAP130, confirmed at both mRNA and protein levels (Figure 4D,E), significantly promoted apoptosis (Figure 4F) and suppressed proliferation, invasion, and migration of RA‐FLSs (Figure 4G–J). Furthermore, mRNA expression of proinflammatory mediators (e.g., CXCL5, CXCL12, JAK2, and JAK3) was markedly downregulated upon SAP130 knockdown (Figure 4K). Conversely, SAP130 overexpression inhibited apoptosis and enhanced proliferative, invasive, and migratory capacities of RA‐FLSs (Figure S5A–G). We also assessed the impact of the SAP130 T473A mutant on the aggressive behaviors of RA‑FLSs. Our results demonstrated that overexpression of the T473A mutant failed to effectively inhibit apoptosis or enhance the proliferative, invasive, and migratory capacities of RA‑FLSs (Figure S6A–D). This functional deficiency is attributable to the inability of the SA130 T473A mutant to undergo O‑GlcNAcylation and subsequent stabilization by OGT, leading to its accelerated degradation via the ubiquitin‑proteasomal pathway. To determine whether SAP130 is required for OGT‑driven pathogenicity, we overexpressed OGT in SAP130‐silenced RA‐FLSs. OGT overexpression failed to restore the aggressive phenotypes impaired by SAP130 knockdown (Figure 4L–O), indicating that OGT's pathogenic role in RA‐FLSs is SAP130‐dependent.

Functionally, SAP130 is a subunit of the Sin3A/HDAC corepressor complex, responsible for recruiting the Sin3A/HDAC1 complex to specific genomic loci to deacetylate histones and silence target genes [42]. Building on this, we investigated whether the OGT‐SAP130 axis regulates histone acetylation in RA‐FLSs. Knockdown of either SAP130 or OGT significantly increased global histone acetylation levels (Figure S7A,B). Importantly, the ability of OGT to suppress acetylation was dependent on SAP130, as OGT overexpression failed to reverse elevated acetylation in SAP130‐silenced RA‐FLSs (Figure S7C). Since the Sin3A/HDAC1 complex recruited by SAP130 is the primary executor of histone deacetylation, we determine whether its enzymatic core, HDAC1, is required for OGT‐driven pathogenic effects. We silenced HDAC1 in RA‐FLSs and found that OGT overexpression failed to promote the aggressive phenotypes of these cells (Figure S7D—G). Collectively, these results reveal that OGT stabilizes SAP130 to facilitate the Sin3A/HDAC1‐mediated histone deacetylation, an essential event driving the aggressive transformation of RA‐FLSs.

### 2.7 The OGT‐SAP130 Axis Reprograms H3K27 Modification to Regulate BTG2 Expression in RA‐FLSs

**FIGURE 5:**
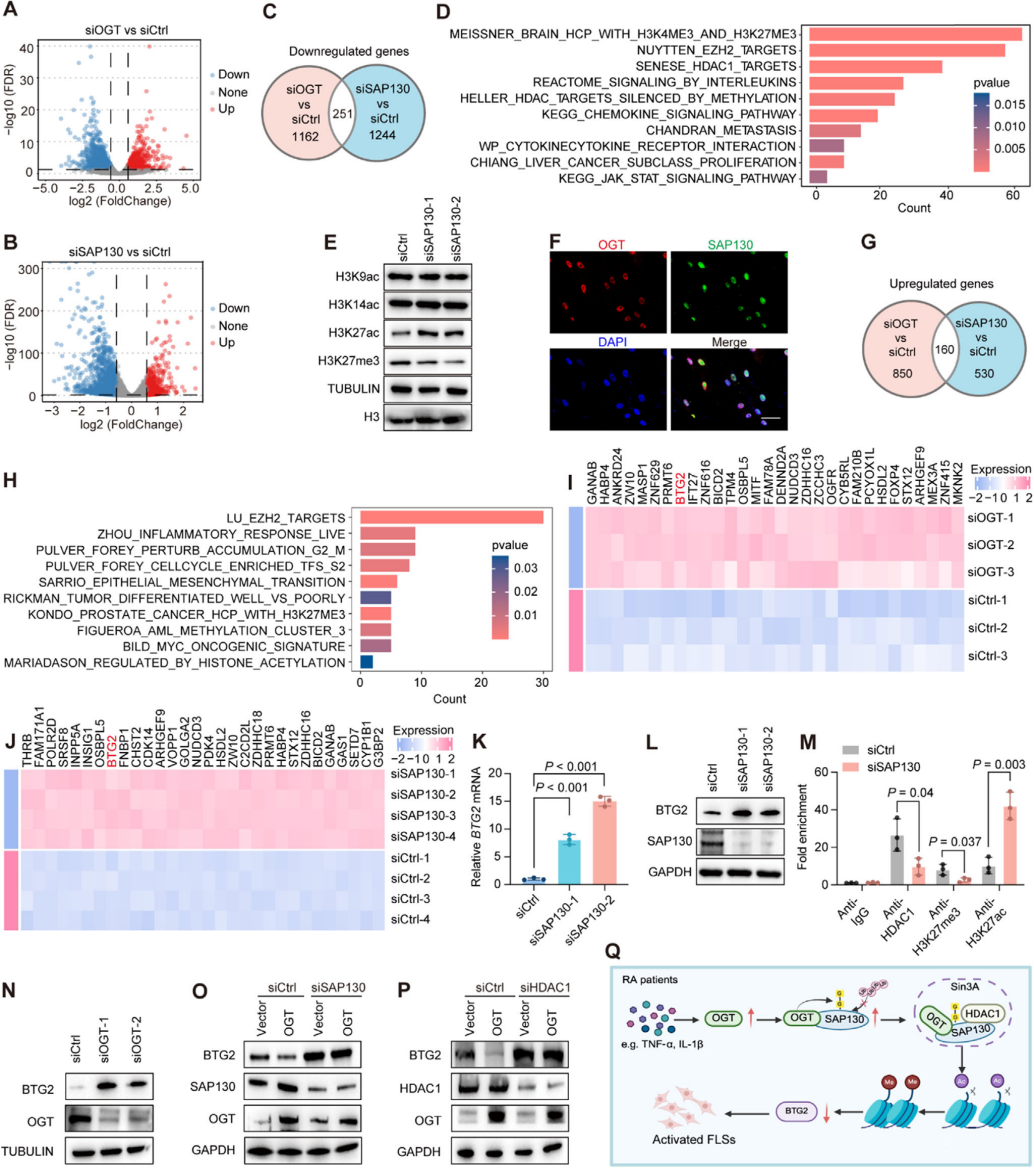
Epigenetic regulation of BTG2 by the OGT‐SAP130 axis. (A) Volcano plot of differentially expressed genes in RA‐FLSs transfected with siOGT versus siCtrl, as determined by RNA sequencing. n = 3. Fold change > 2, *P* < 0.05. (B) Volcano plot of differentially expressed genes in RA‐FLSs transfected with siSAP130 versus siCtrl, as determined by RNA sequencing. n = 4. Fold change > 2, *P* < 0.05. (C) Venn diagram showing the overlapping set of downregulated genes in RA‐FLSs transfected with siOGT (versus siCtrl, n = 3) and those from siSAP130 (versus siCtrl, n = 4). (D) Pathway enrichment analysis of the overlapping downregulated genes in RA‐FLSs transfected with siOGT (versus siCtrl, n = 3) and those from siSAP130 (versus siCtrl, n = 4). Fold change > 2, *P* < 0.05. (E) Levels of histone modifications (H3K9ac, H3K14ac, H3K27ac, and H3K27me3) in RA‐FLSs after transfection with siCtrl, siSAP130‐1, or siSAP130‐2. n = 3. (F) Colocalization of OGT with SAP130 in RA‐FLSs. n = 3. Scale bars, 50 µm. (G) Venn diagram showing the overlapping set of upregulated genes in RA‐FLSs transfected with siOGT (versus siCtrl, n = 3) and those from siSAP130 (versus siCtrl, n = 4). (H) Pathway enrichment analysis of the overlapping upregulated genes in RA‐FLSs transfected with siOGT (versus siCtrl, n = 3) and those from siSAP130 (versus siCtrl, n = 4). Fold change > 2, *P* < 0.05. (I) Heatmap of the top 30 upregulated genes in RA‐FLSs transfected with siOGT versus siCtrl. n = 3. (J) Heatmap of the top 30 upregulated genes in RA‐FLSs transfected with siSAP130 versus siCtrl. n = 4. (K and L) mRNA (K) and protein (L) levels of BTG2 in RA‐FLSs transfected with siCtrl, siSAP130‐1, or siSAP130‐2. n = 3. (M) ChIP‐qPCR analysis of HDAC1, H3K27me3, and H3K27ac at the *BTG2* promoter in RA‐FLSs transfected with siCtrl or siSAP130. n = 3. (N) BTG2 protein levels in RA‐FLSs transfected with siCtrl, siOGT‐1, or siOGT‐2. n = 3. (O) BTG2 protein levels in SAP130‐silenced RA‐FLSs after transfection with the empty vector or the OGT‐overexpressing vector. n = 3. (P) BTG2 protein levels in HDAC1‐silenced RA‐FLSs after transfection with the empty vector or the OGT‐overexpressing vector. n = 3. (Q) The regulatory model of the OGT‐SAP130‐BTG2 axis. Data are presented as mean ± SD, and statistical significance is calculated by one‐way ANOVA with Dunnett's test (K) and unpaired *t*‐test (M).

We performed transcriptome profiling via RNA‐seq on RA‐FLSs following knockdown of OGT or SAP130 (Figure 5A,B). Comparative analysis identified 251 genes that were consistently downregulated upon silencing of either OGT or SAP130 (Figure 5C). Pathway analysis revealed that these genes were enriched in inflammation‑related pathways (e.g., chemokine, cytokine, and JAK signaling), cell proliferation, and metastasis (Figure 5D), aligning with the pro‑aggressive role of the OGT‑SAP130 axis in RA‐FLSs. Notably, we observed that epigenetic pathways related to trimethylation of histone H3 at lysine 27 (H3K27me3) and HDAC1‐mediated histone deacetylation were also significantly enriched (Figure 5D). Given this strong epigenetic signature, we assessed the impact of SAP130 on relevant histone modifications. Silencing SAP130 in RA‐FLSs specifically increased the activating mark histone H3 lysine 27 acetylation (H3K27ac) and decreased the repressive mark H3K27me3, without affecting H3K9ac or H3K14ac levels (Figure 5E). This shift in the H3K27ac/H3K27me3 balance, together with SAP130's known role in recruiting the Sin3A/HDAC1 complex, supports a model wherein the OGT‐SAP130 axis regulates gene expression by modulating this key histone modification switch. Further, we confirmed prominent nuclear colocalization of OGT and SAP130 in RA‐FLSs (Figure 5F), demonstrating their spatial proximity for coordinated epigenetic regulation.

We also analyzed genes upregulated upon knockdown of OGT or SAP130 in RA‐FLSs. Transcriptomic profiling identified 160 consistently upregulated genes (Figure 5G), which were strongly associated with cell‑cycle regulation, oncogenic progression, inflammatory response, and histone modification (Figure 5H). Among the top 30 upregulated genes in OGT‑ or SAP130‑silenced cells, BTG2 emerged as a common gene (Figure 5I,J) and was selected for further study given its established role as an antiproliferative gene [21, 22]. Consistent with this, SAP130 knockdown significantly increased both BTG2 mRNA and protein levels in RA‑FLSs (Figure 5K,L). To determine whether the Sin3A/HDAC1 complex is directly recruited to the BTG2 promoter by SAP130, we performed chromatin immunoprecipitation followed by quantitative PCR (ChIP‑qPCR) in SAP130‑silenced RA‑FLSs. Silencing SAP130 markedly reduced HDAC1 and H3K27me3 occupancy while substantially increasing H3K27ac enrichment at the *BTG2* promoter (Figure 5M). Similarly, OGT knockdown also significantly upregulated BTG2 expression (Figure 5N). Importantly, OGT overexpression failed to suppress the elevated BTG2 levels in SAP130‑silenced (Figure 5O) or HDAC1‑silenced RA‑FLSs (Figure 5P), demonstrating that the OGT‐SAP130 axis‑mediated repression of BTG2 depends on the Sin3A/HDAC1 complex. In addition, we overexpressed SAP130 in BTG2‑silenced RA‑FLSs. Notably, SAP130 overexpression failed to further inhibit apoptosis, promote proliferative, invasive, or migratory capacities of BTG2‑silenced cells, or upregulate expression of the proinflammatory mediators, including *CXCL5*, *CXCL12*, *JAK2*, and *JAK3* (Figure S8A–E). These results collectively indicate that BTG2 serves as the critical molecular bridge connecting the OGT‑SAP130 axis to the aggressive phenotypes of RA‑FLSs. Collectively, our data support a working model wherein inflammatory stimuli activate the OGT‑SAP130 axis, which in turn recruits the Sin3A/HDAC1 complex to modulate the H3K27ac/H3K27me3 balance and epigenetically silence BTG2, thereby promoting the aggressive transformation of RA‑FLSs (Figure 5Q).

### 2.8 The OGT Inhibitor OSMI‐1 Shows Limited Efficacy in CIA Mice

Since we demonstrated that OSMI‐1 inhibited the aggressive transformation of RAFLSs in vitro, we evaluated its therapeutic potential in vivo. CIA mice were treated systemically with vehicle (Veh), OSMI‐1, the TNF‐α inhibitor ETA, or a combination of OSMI‐1 and ETA (OSMI‐1 + ETA) (Figure S9A). Although OSMI‐1 initially induced a modest reduction in arthritis scores, the effect was not sustained, and scores remained significantly elevated throughout the treatment period (Figure S9B). Micro‐computed tomography (micro‐CT) and histopathological analysis (SO/FG and HE staining) revealed that OSMI‐1 treatment did not substantially ameliorate joint destruction, synovitis, bone erosion, or cartilage destruction compared to the Veh group (Figure S9C–E). In contrast, ETA treatment resulted in a significant and sustained suppression of arthritis scores (Figure S9B) and provided robust protection against structural joint destruction, as evidenced by micro‐CT imaging, SO/FG, and HE staining (Figure S9C–E). Notably, combining OSMI‐1 with ETA did not confer a significant additive benefit compared to ETA alone (Figure S9B–E). These in vivo findings demonstrated that systemic inhibition of OGT with OSMI‐1 offers only limited therapeutic efficacy in CIA mice.

### 2.9 An AS1411‐Based PROTAC is Designed for Selectively Degrading OGT in RA‐FLSs

**FIGURE 6:**
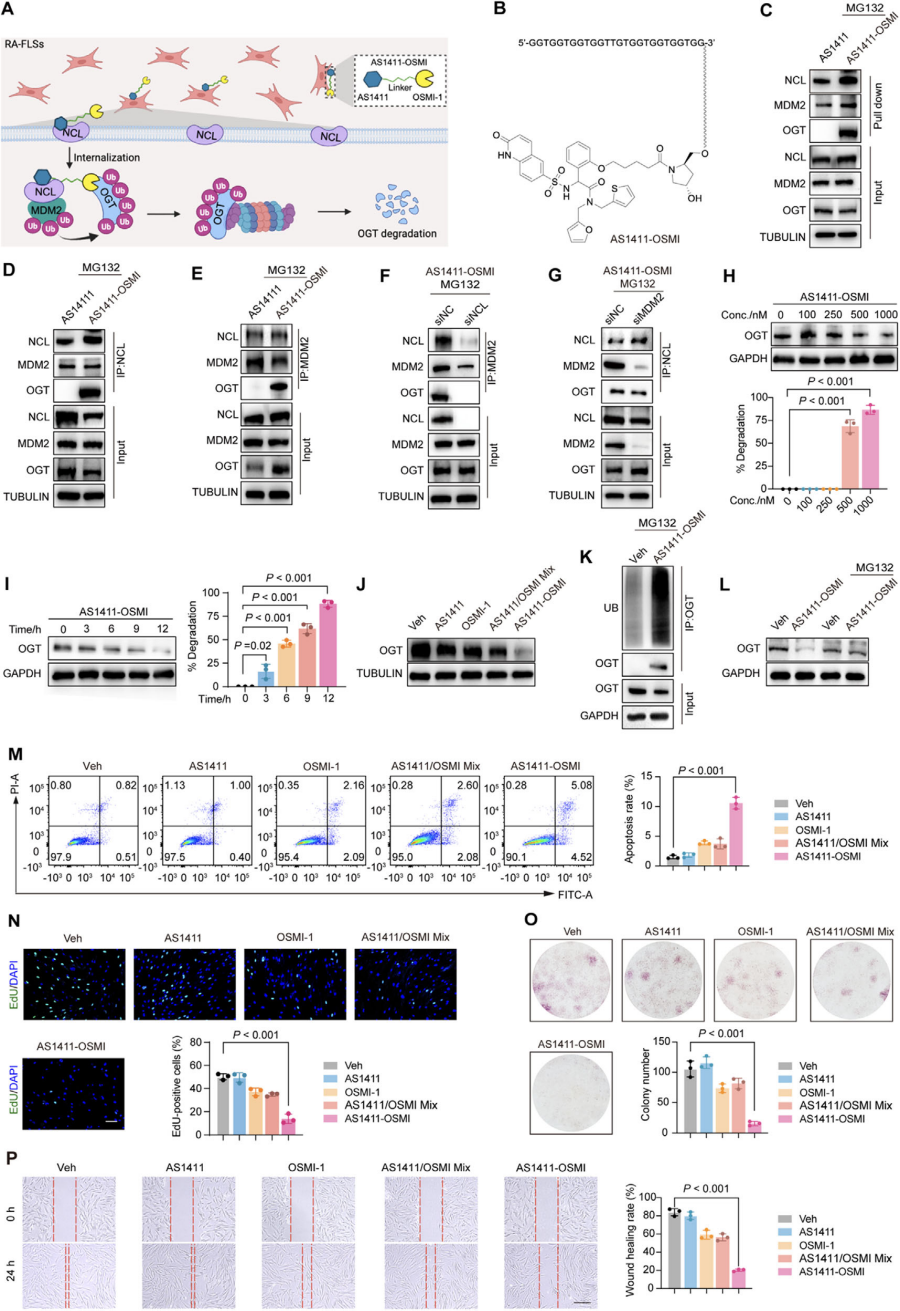
Effects of AS1411‐OSMI on OGT expression and phenotype of RA‐FLSs. (A) Schematic illustration of the AS1411‐based PROTAC design and its mechanism of action. The heterobifunctional molecule AS1411‐OSMI is constructed by covalently conjugating AS1411 with OSMI‐1. This conjugate selectively binds to NCL, which is overexpressed on the surface of RA‐FLSs, thereby facilitating its cellular internalization. Once inside RA‐FLSs, AS1411‐OSMI mediates the formation of a quaternary complex composed of MDM2, NCL, PROTAC, and OGT, ultimately promoting the ubiquitination and subsequent degradation of OGT. (B) Chemical structure of AS1411‐OSMI. (C) Pull‐down assay of NCL, MDM2, and OGT using streptavidin‐coated agarose beads for RA‐FLSs after treatment with 500 nM biotin‐labeled AS1411 or AS1411‐OSMI for 6 h, in the presence of 5 µM MG132. n = 3. (D and E) Co‐IP of NCL, MDM2, and OGT for RA‐FLSs after treatment with 500 nM AS1411 or AS1411‐OSMI for 6 h using an anti‐NCL antibody (D) or an anti‐MDM2 antibody (E), in the presence of 5 µM MG132. n = 3. (F) Co‐IP of NCL, MDM2, and OGT in siNCL‐transfected RA‐FLSs treated with 500 nM AS1411‐OSMI for 6 h using an anti‐MDM2 antibody, in the presence of 5 µM MG132. n = 3. (G) Co‐IP of NCL, MDM2, and OGT in siMDM2‐transfected RA‐FLSs treated with 500 nM AS1411‐OSMI for 6 h using an anti‐NCL antibody, in the presence of 5 µM MG132. n = 3. (H) OGT protein levels in RA‐FLSs after treatment with AS1411‐OSMI at the indicated concentrations for 12 h. Quantification is shown on the bottom. n = 3. (I) OGT protein levels in RA‐FLSs after treatment with 1 µM AS1411‐OSMI at the indicated time points. Quantification is shown on the right. n = 3. (J) OGT protein levels in RA‐FLSs treated with Veh, AS1411, OSMI‐1, AS1411/OSMI Mix, or AS1411‐OSMI at a concentration of 1µM for 12 h. n = 3. (K) Ubiquitination levels of OGT in RA‐FLSs after treatment with Veh or 1 µM AS1411‐OSMI for 12 h, in the presence of 5 µM MG132. n = 3. (L) OGT protein levels in RA‐FLSs treated with Veh or 1 µM AS1411‐OSMI for 12 h, in the presence or absence of 5 µM MG132. n = 3. (M) Apoptosis of RA‐FLSs treated with Veh, AS1411, OSMI‐1, AS1411/OSMI Mix, or AS1411‐OSMI at a concentration of 1 µM. Quantification is shown on the right. n = 3. (N) Proliferation of RA‐FLSs treated with Veh, AS1411, OSMI‐1, AS1411/OSMI Mix, or AS1411‐OSMI at a concentration of 1 µM, as measured by EdU staining. Quantification is shown on the bottom right. n = 3. Scale bars,100 µm. (O) Colony formation of RA‐FLSs treated with Veh, AS1411, OSMI‐1, AS1411/OSMI Mix, or AS1411‐OSMI at a concentration of 1 µM. Quantification is shown on the bottom right. n = 3. (P) Wound healing assay of RA‐FLSs treated with Veh, AS1411, OSMI‐1, AS1411/OSMI Mix, or AS1411‐OSMI at a concentration of 1 µM. Quantification is shown on the right. n = 3. Scale bars, 50 µm. Data are presented as mean ± SD, and statistical significance is calculated by one‐way ANOVA with Tukey's test (M‐P) or Dunnett's test (H and I).

We attributed the limited in vivo efficacy of OSMI‑1 to its poor cellular specificity for pathogenic FLSs. To address this, we aimed to develop a pathogenic FLS‑targeted OGT degrader using PROTAC technology [24]. This strategy is built on two key foundations: (i) our prior identification of NCL as a highly expressed surface protein on pathogenic FLSs [26], and (ii) our established cell‑selective PROTAC platform centered on NCL‐targeting AS1411 [25]. In this platform, AS1411 serves a dual role: it acts as a cell‑targeting ligand by binding to NCL overexpressed on diseased cells, and upon internalization, it engages the cytoplasmic NCL‐MDM2 interaction to recruit the E3 ligase MDM2, enabling cell‑type‑restricted protein degradation when linked to a target protein‑binding ligand [25]. Prior to constructing the pathogenic FLS‑targeted OGT degrader, we validated the targeting specificity of the AS1411. AS1411 exhibited time‐ and concentration‐dependent binding to RA‐FLSs in vitro (Figure S10A–C). Over 60% of the AS1411 was efficiently internalized into RA‐FLSs within the incubation period (Figure S10D). This high uptake efficiency is consistent with the well‐characterized property of AS1411 in surface NCL‐overexpressing tumor cells, wherein it exploits NCL‐dependent binding to achieve rapid and robust cellular entry [43]. We next examined the intracellular trafficking of internalized AS1411 by assessing its colocalization with lysosomal markers in RAFLSs. Notably, we observed minimal overlap between the AS1411 signal and lysosomes, indicating that AS1411 does not accumulate in degradative compartments (Figure S10E). This observation aligns with earlier studies demonstrating that AS1411 is primarily internalized into tumor cells via micropinocytosis, an uptake route that favors endosomal escape [44]. Such trafficking behavior would provide a mechanistic basis for the efficient intracellular recruitment of MDM2 by our AS1411‐based PROTAC, thereby enabling subsequent OGT ubiquitination and degradation. Further, in vivo and ex vivo imaging of AS1411 confirmed its specific accumulation and enrichment in inflamed hind paws and synovium in CIA mice (Figure S10F,G). Then, we conjugated AS1411 to OSMI‑1 to generate the heterobifunctional PROTAC, AS1411‑OSMI (Figure 6A). In our previous study, we found that AS1411 conjugates with the ligand attached at the 3' terminus achieved significantly greater degradation efficacy than those with 5'‑terminal conjugation [25]. We therefore selected the 3' position for conjugating OSMI‑1 in this PROTAC design (Figure 6B and Scheme S1). The proposed mechanism involves AS1411‑mediated recognition of surface‑exposed NCL on pathogenic FLSs, which facilitates selective cellular internalization while sparing non‐FLS cells with low or absent surface NCL. Following entry, AS1411 engages the cytoplasmic NCL‐MDM2 interaction to recruit MDM2, thereby directing OGT for ubiquitination and degradation in a cell‑type‑restricted manner (Figure 6A).

### 2.10 AS1411‐OSMI Enables Assembly of the MDM2‐NCL‐PROTAC‐OGT Quaternary Complex

The central premise of PROTAC technology is the induced proximity between a target protein and an E3 ligase [45]. We therefore hypothesized that AS1411‐OSMI operates through this mechanism by bringing OGT into proximity with the NCL‐MDM2 complex. Our pull‐down assays verified that the unconjugated AS1411 only pulled down NCL and MDM2 but not OGT in RA‐FLSs, whereas AS1411‐OSMI successfully pulled down NCL, MDM2, and OGT (Figure 6C). We also treated RA‐FLSs with AS1411 or AS1411‐OSMI and performed co‐IP assays. Our results demonstrated that MDM2 and OGT were co‐precipitated with NCL in RA‐FLSs incubated with AS1411‐OSMI but not in cells treated with AS1411 (Figure 6D). We also showed the efficient co‐precipitation of NCL and OGT with MDM2 in RA‐FLSs with the presence of AS1411‐OSMI (Figure 6E). These results indicated that AS1411‐OSMI enabled the formation of MDM2‐NCL‐PROTAC‐OGT quaternary complexes. Upon silencing NCL in RA‐FLSs, we observed that, with the presence of AS1411‐OSMI, MDM2 failed to co‐precipitate OGT (Figure 6F), while OGT still was co‐precipitated with NCL in MDM2‐silenced RA‐FLSs (Figure 6G). These findings confirmed that NCL acts as an essential molecular bridge between MDM2 and OGT in the assembly of the MDM2‐NCL‐PROTAC‐OGT quaternary complexes.

### 2.11 AS1411‐OSMI Induces OGT Degradation and Suppresses Aggressive Phenotypes in RA‐FLSs

We determined the ability of AS1411‐OSMI to degrade OGT in RA‐FLSs in vitro. Treatment with AS1411‐OSMI resulted in a dose‐ and time‐dependent reduction in OGT protein levels (Figure 6H,I). This degradation was specific to pathogenic FLSs, as no OGT degradation was observed in surface NCL‐negative ATDC5 chondroprogenitor cells or MC3T3‐E1 preosteoblasts [26] (Figure S11A,B). When RA‐FLSs were treated with Veh, AS1411, OSMI‐1, a physical mixture of AS1411 and OSMI‐1 (AS1411/OSMI Mix), or AS1411‐OSMI, only AS1411‐OSMI effectively induced OGT degradation (Figure 6J). AS1411‐OSMI treatment significantly enhanced OGT ubiquitination (Figure 6K), and the OGT degradation induced by AS1411‐OSMI was abolished by the proteasome inhibitor MG132 (Figure 6L), confirming its dependence on the ubiquitin‐proteasome pathway. We next evaluated the efficacy of AS1411‐OSMI in suppressing aggressive phenotypes of RA‐FLSs in vitro. Notably, even at a substantially lower dose (1 µM) than the effective concentration of OSMI‐1 used in the above in vitro study (40 µM), AS1411‐OSMI treatment significantly increased apoptosis in RA‐FLSs compared to Veh, AS1411, OSMI‐1, or the AS1411/OSMI Mix, as determined by flow cytometry (Figure 6M). EdU staining and colony formation assays further showed that AS1411‐OSMI remarkably inhibited the proliferation of RA‐FLSs (Figure 6N,O). Wound healing assays revealed that AS1411‐OSMI significantly suppressed migration compared to other treatments (Figure 6P). In contrast, OSMI‐1 at an equivalent dose (1 µM) only had a mild effect on modulating these aggressive phenotypes in RA‐FLSs in vitro (Figure 6M–P). Collectively, these results demonstrate that targeted degradation of OGT by AS1411‐OSMI is more effective than enzymatic inhibition by OSMI‑1 in suppressing pathogenic functions of RA‐FLSs.

### 2.12 AS1411‐OSMI Monotherapy or Combination Therapy With ETA Alleviates Arthritis in CIA Mice

**FIGURE 7:**
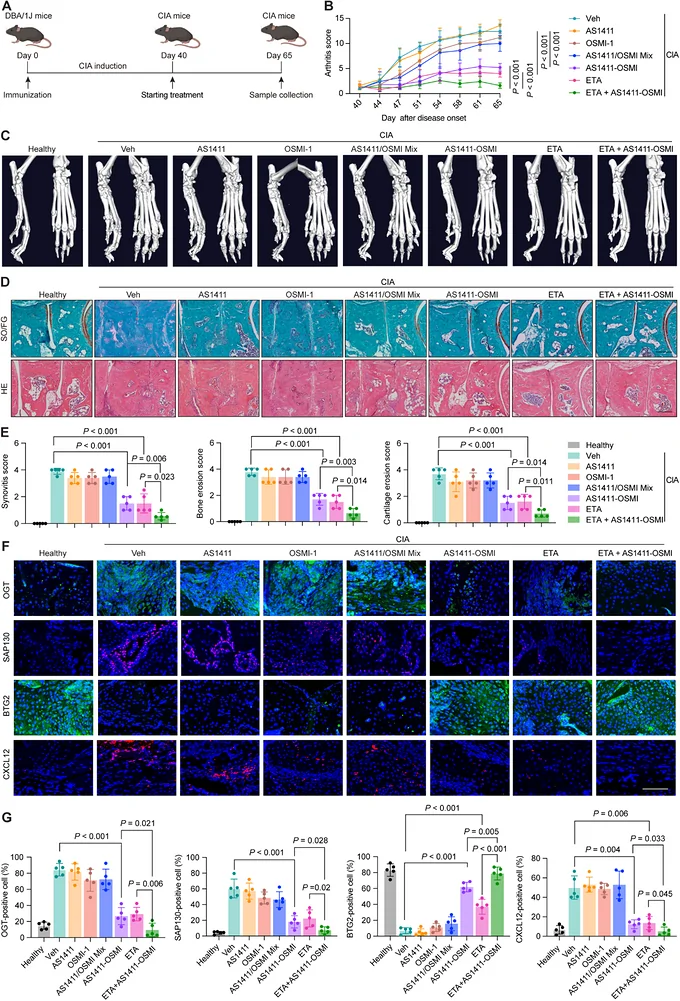
Therapeutic effects of AS1411‐OSMI alone or in combination with ETA in vivo. (A) Experimental design for CIA mouse models. DBA/1J mice were immunized with CII emulsified in CFA to induce CIA. Following CIA establishment, CIA mice received systemic treatments with Veh, AS1411, OSMI‐1, AS1411/OSMI Mix, AS1411‐OSMI, ETA, or ETA + AS1411‐OSMI for 25 days. The dosage of AS1411OSMI was determined as 2.5 µmol/kg based on preliminary doseresponse experiments, in which this concentration yielded maximal OGT degradation in vivo. AS1411, OSMI‐1, or AS1411/OSMI Mix were given at equimolar doses for direct comparison. ETA was intraperitoneally administered at 5 mg/kg, and non‐immunized mice served as healthy controls. (B) Arthritis scores of CIA mice from different treatment groups. n = 5. (C) Micro‐CT images of the hind paws of healthy controls or CIA mice from different treatment groups. n = 5. (D) Representative images of SO/FG and HE staining on sections of the hind paws of healthy controls or CIA mice from different treatment groups. n = 5. Scale bars, 200 µm. (E) Quantification of synovitis, bone, and cartilage erosion on SO/FG‐ and HE‐stained sections. n = 5. (F) IF staining of OGT, SAP130, BTG2, and CXCL12 on sections of the hind paws of healthy controls or CIA mice from different treatment groups. n = 5. Scale bar, 100 µm. (G) Quantification of positive cells (%) for IF staining of OGT, SAP130, BTG2, and CXCL12. n = 5. Data are presented as mean ± SD, and statistical significance is calculated by two‐way ANOVA (B) and one‐way ANOVA with Tukey's test (E and G).

We administered CIA mice with Veh, AS1411, OSMI‐1, AS1411/OSMI Mix, AS1411‐OSMI, ETA, or a combination of ETA with AS1411‐OSMI (ETA + AS1411‐OSMI) (Figure 7A). Compared to Veh, AS1411, OSMI, or AS1411/OSMI Mix, AS1411‐OSMI effectively reduced the arthritic score in CIA mice (Figure 7B). Micro‐CT analysis, histological staining, and quantitative analysis demonstrated that AS1411‐OSMI attenuated synovitis, bone erosion, and cartilage destruction in CIA mice, achieving efficacy comparable to that of ETA (Figure 7C–E). Strikingly, combination therapy with AS1411‐OSMI and ETA yielded an additive therapeutic benefit, outperforming either monotherapy across both clinical and structural endpoints (Figure 7B–E). IF analysis of synovial tissues from treated mice demonstrated that AS1411‐OSMI significantly downregulated the expression of OGT, SAP130, and CXCL12, while markedly upregulated BTG2 level (Figure 7F,G). To rigorously evaluate the in vivo cell selectivity and safety of our PROTAC, we examined OGT expression in the spleen, which contains various immune cells, notably B cells reported to also express surface NCL, albeit at relatively low levels [46]. OGT was not degraded in these immune cells, as evidenced by comparable presence of OGT in CD45R‐positive B cells between AS1411‑OSMI‑treated and Veh‑treated mice (Figure S12A). Furthermore, we confirmed that surface NCL levels on FLSs were markedly higher than those on B cells from AS1411‑OSMI‑treated mice (Figure S12B), explaining the selective OGT degradation in FLSs and the minimal off‑target effects. Comprehensive serum biochemistry analysis revealed that AS1411‐OSMI, either alone or in combination with ETA, did not induce significant hepatotoxicity or nephrotoxicity, as assessed by albumin (ALB), alkaline phosphatase (ALP), alanine aminotransferase (ALT), aspartate Aminotransferase (AST), total protein (TP), creatinine (CR), uric acid (UA), and blood urea nitrogen (BUN) (Figure S12C–J). Furthermore, no significant damage to major organs, including the heart, liver, spleen, lung and kidney, was observed in the mice after treatment with AS1411‐OSMI, either alone or in combination with ETA (Figure S12K).

## 3 Discussion

RA remains a persistent therapeutic challenge, not owing to a paucity of immunosuppressive agents, but because stroma‐driven joint destruction frequently progresses even under ostensibly controlled inflammation [6, 7]. Here, we establish a comprehensive translational framework that integrates AI‐guided target discovery, mechanistic deconvolution, and the rational engineering of a cell‐type‐specific degradation therapy designed to directly intervene within this pathogenic stromal compartment. Our findings identify OGT as a previously unrecognized contributor that orchestrates the aggressive epigenetic reprogramming of disease‐propagating FLSs, and demonstrate that its targeted degradation via an aptamer‐based PROTAC can achieve precise and selective attenuation of FLS pathogenicity while maintaining a favorable safety profile. Beyond RA, this work provides a generalizable framework for tackling other diseases driven by pathological stromal fibroblasts, such as systemic sclerosis and cancer‐associated fibroblast‐dependent malignancies [47, 48].

The application of AI to scRNA‐seq data has emerged as a powerful strategy to deconvolve cellular heterogeneity and nominate disease drivers in an unbiased manner [49]. In this study, through an AI‐guided analysis of synovial scRNA‐seq data that integrates the scTransMIL framework with SCimilarity, we pinpointed OGT as an FLS‐enriched hub gene whose expression correlates with pathogenic synovial subsets in RA. This computational nomination, validated by gain‐ and loss‐of‐function genetics including FLS‐specific OGT knockout, establishes OGT as a critical driver of the aggressive phenotypes of pathogenic FLSs. Notably, conventional differential expression approaches, which rely on predefined markers or bulk‐derived comparisons, failed to prioritize OGT with the same specificity, underscoring the power of hypothesis‐free AI architectures to unmask context‐dependent pathogenic regulators that might otherwise remain obscured by cellular heterogeneity [50]. This integrated computational strategy thus provides a blueprint for AI‐driven target discovery in complex chronic inflammatory diseases.

Accumulating evidence indicates that diverse epigenetic alterations, including DNA methylation, histone methylation, histone acetylation, and histone phosphorylation, contribute to the imprinted pathological phenotypes of FLSs in RA [51]. Our mechanistic findings add a new dimension to this paradigm by revealing that OGT‐mediated O‐GlcNAcylation stabilizes SAP130, a subunit of the Sin3A/HDAC corepressor complex [42]. This stabilization licenses the Sin3A/HDAC complex to silence BTG2 through coordinated epigenetic remodeling. Specifically, we observed that OGT‐driven SAP130 stabilization promotes localized histone deacetylation at the *BTG2* gene promoter, leading to the loss of the activation‐associated H3K27ac mark [52], while simultaneously facilitating deposition of the repressive H3K27me3 modification [53]. This shift in the H3K27ac/H3K27me3 balance, from a transcriptionally permissive to a repressive chromatin state, results in durable epigenetic silencing of BTG2, thereby fueling FLS aggression. This signaling cascade, which links OGT‐mediated O‐GlcNAcylation to stable reprogramming of the FLS epigenome, positions OGT as a key gatekeeper of stromal pathology. Notably, this mechanism delineates a p53‐independent regulatory axis for BTG2, a gene classically characterized as a transcriptional target of p53 [54, 55]. Moreover, our observation that OGT‐mediated O‐GlcNAcylation stabilizes SAP130 by inhibiting its ubiquitination and proteasomal degradation aligns with previous reports demonstrating that O‐GlcNAcylation shields diverse substrate proteins from proteasomal turnover [39, 40, 41].

The OGT inhibitor OSMI‑1 effectively suppressed the aggressive phenotypes of RA‑FLS in vitro, consistent with a central role for OGT in driving FLS pathogenicity. However, despite this clear on‑target activity, systemic administration of OSMI‑1 failed to achieve sustained therapeutic benefit in CIA mice. This striking disconnect between potent in vitro efficacy and limited in vivo performance underscores a fundamental challenge in systemically targeting ubiquitously expressed enzymes [56]. We attribute this limitation primarily to the poor cellular specificity of OSMI‑1, which lacks preferential accumulation in pathologic FLSs. Importantly, recent work from our laboratory has revealed that OGT plays a protective role in osteoblast differentiation and chondrocyte development [40, 57]. Thus, systemic OGT inhibition carries a dual liability: suboptimal efficacy due to inadequate joint enrichment, coupled with the risk of on‑target toxicity in bone and cartilage compartments where OGT activity is physiologically essential. These considerations motivated the development of a cell‑selective degradation strategy designed to uncouple therapeutic OGT suppression in pathogenic FLSs from preservation of its essential functions in healthy skeletal tissues.

The AS1411‐OSMI conjugate represents a next‐generation PROTAC paradigm in which cell‐type selectivity is conferred not by the target‐protein warhead OSMI‑1, but by the NCL‐targeting AS1411 aptamer itself. By co‐opting NCL, a surface protein we identified as highly and specifically expressed on pathogenic FLSs [26], as both an internalization portal and a scaffold for MDM2 recruitment, this architecture achieves dual‐layer specificity. First, cellular entry is restricted to pathogenic FLSs with high surface NCL expression, ensuring that the PROTAC is internalized preferentially by disease‐relevant cells. Second, once internalized, degradation proceeds only upon concurrent engagement of intracellular NCL and the E3 ligase MDM2 [25], both of which are abundantly expressed in pathogenic FLSs [26, 58]. This strategy effectively converts the pan‐cell inhibitor into a cell‐type‐selective therapeutic. The failure of unconjugated OSMI1, AS1411 alone, or simple physical mixtures to recapitulate OGT degradation underscores the essential role of covalent conjugation in enabling targeted protein degradation. Importantly, the modular nature of this aptamer‐based PROTAC platform, wherein the warhead can be swapped while retaining the NCL‐targeting moiety, provides a versatile framework for developing additional stromal FLS‐targeted therapies by simply exchanging the target‐binding ligand.

Systemic administration of AS1411‐OSMI resulted in profound OGT degradation and significant histological protection in CIA mice, with efficacy comparable to TNF‐α inhibition. Comprehensive serum biochemistry profiling revealed no detectable hepatotoxicity or nephrotoxicity, underscoring the safety of this targeted approach. Critically, OGT protein levels remained unchanged in nontargeted cell types, including chondroprogenitors and preosteoblasts in vitro and B cells in vivo, confirming that the PROTAC selectively spares lineages vulnerable to pan‑OGT inhibition. These observations establish a favorable therapeutic window for AS1411OSMI and validate the NCL‐directed PROTAC platform as a clinically viable strategy for stromal‐selective intervention. Moreover, beyond its monotherapeutic efficacy, the AS1411‐OSMI conjugate demonstrated additive therapeutic benefit when combined with ETA, a TNF‐α inhibitor widely used in RA management [53]. This combinatorial efficacy highlights the orthogonal logic of pairing systemic cytokine blockade with precise stromal targeting, a rational combination strategy that simultaneously engages both the immune and stromal pillars of RA pathogenesis. Notably, the AS1411 aptamer used in this study is an unmodified ssDNA oligonucleotide without phosphorothioate linkages, 2'‐O‐methyl substitutions, 3'‐inverted dT, or other stabilizing chemical modifications [59]. Normally, unmodified ssDNA is susceptible to rapid nuclease degradation in serum. However, AS1411 adopts a stable G‑quadruplex (G4) structure under physiological conditions, which confers intrinsic resistance to nuclease‑mediated degradation by reducing the accessibility of the phosphodiester backbone to serum nucleases [60, 61]. This structural protection, together with the rapid targeting and internalization by pathogenic FLSs that overexpress surface NCL in the inflamed synovium, likely provides sufficient local drug exposure to achieve the observed therapeutic efficacy.

Several considerations warrant further investigation in future studies. First, the molecular mechanisms underlying OGT upregulation in response to inflammatory stimuli (such as TNF‐α and IL‐1β) in pathogenic FLSs remain to be fully elucidated. Identifying the upstream signaling pathways that drive OGT expression under inflammatory conditions could reveal additional nodes for therapeutic intervention. Second, although the AS1411‐OSMI conjugate demonstrates potent and selective activity, further optimization of the PROTAC scaffold, particularly the composition, length, and conjugation site of the linker, may enhance degradation efficiency and pharmacokinetic properties [62]. Third, our functional assays and in vivo data collectively support the FLS‑specific OGT degradation achieved through NCL‐mediated targeting, future unbiased proteomic studies are warranted to fully assess PROTAC selectivity and rule out potential off‑target effects. Fourth, the heterogeneity of human RA, including variations in serotypes and treatment backgrounds, necessitates future validation in larger, stratified, multi‑center cohorts to fully establish the clinical utility of the OGT/SAP130/BTG2 axis across diverse patient subpopulations. In addition, more comprehensive studies, including detailed biodistribution, clearance, and joint accumulation analyses, will be essential for fully characterizing the in vivo behavior of AS1411‐OSMI. Addressing these questions will be essential for realizing the full therapeutic potential of this stromal‐targeted degradation strategy.

In summary, this study establishes a new RA therapeutic modality that targets the autonomous, epigenetically reprogrammed FLSs rather than the infiltrating immune system. By coupling the discovery power of AI with the mechanistic specificity of targeted protein degradation, we demonstrate that the stromal pathogenic compartment is not only targetable but also druggable with high precision. This paradigm shift, from systemic immunosuppression to cell‐selective stromal rewiring, may offer a path to transcend the current therapeutic ceiling in RA.

## 4 Materials and Methods

### 4.1 AI‐Based scRNA‐Seq Analysis

This study employed an AI‐driven automated pipeline to analyze scRNA‐seq data. First, quality control was performed on the raw data using Scanpy [63]. Subsequently, based on quality‐controlled data, the attention‐based multiple instance learning framework of the scTransMIL model was utilized to calculate disease correlation scores for each cell [64]. Building on this, we adopted a dual‐threshold strategy to classify cells. A strict threshold of 0.99 was used to identify high‐confidence RA‐associated cells, while a moderate threshold of 0.97 was applied to capture a broader population of disease‐associated cells. To decipher the genetic features associated with the disease, we further applied the SCimilarity interpretation tool [64]. By comparing disease‐state cells with healthy‐state cells, it generated gene frequency plots and ranked genes based on their association strength. It identified threshold‐sensitive genes by comparing the dual‐threshold results. All analyses were conducted in a Python 3.10 environment, utilizing key packages such as scTransMIL, SCimilarity, Scanpy, as well as Matplotlib and Seaborn.

### 4.2 Collection of Human Samples

Synovial tissues were obtained from RA and OA patients who underwent knee joint replacement surgery and from non‐arthritic individuals undergoing knee surgery for traumatic injuries, and their basic information is shown in Table S1. All clinical procedures were approved by the Committees of Clinical Ethics in the Guanghua Hospital Affiliated to Shanghai University of Traditional Chinese Medicine (GHYYYBC003). We obtained informed consent from the participants.

### 4.3 Cell Culture

RA‐FLSs and mouse FLSs were isolated from synovial tissues [65, 66, 67]. Mouse osteoblasts were isolated from long bones [68, 69]. Mouse chondrocytes were isolated from articular cartilage of knee joints [70, 71]. Mouse B cells were isolated from spleens [72]. MC3T3‐E1 preosteoblasts (subclone 4, CRL‐2593) and 293T cells (CRL‐3216) were obtained from American Type Culture Collection (ATCC). Chondroprogenitor cell line ATDC5 (99072806) was obtained from the European Collection of Authenticated Cell Cultures (ECACC). RA‐FLSs were maintained in Dulbecco's Modified Eagle's medium (DMEM) (Corning, Cat# 10‐013‐CVRC) supplemented with 20% fetal bovine serum (FBS) (Corning, Cat# 35‐081‐CV). Mouse FLSs and 293T cells were cultured in DMEM medium supplemented with 10% FBS. MC3T3‐E1 cells were cultured in α‐Minimum Essential Medium (α‐MEM) (Gibco, Cat# 12571063) with 10% FBS supplementation. ATDC5 cells were cultured in a 1:1 mixture of DMEM and Ham's F12 medium (DMEM/F12) supplemented with 10% FBS. All media were supplemented with 1% penicillin‐streptomycin (Gibco, Cat# 15140122). All cultured cells were confirmed to be mycoplasma‐free and cultured in a humidified incubator at 37°C with 5% CO_2_, with the culture medium refreshed every 2–3 days. For the identification of RA‐FLSs, IF staining was performed using specific cell‐surface markers, vimentin (1: 200, Abcam, Cat# ab92547) and THY1 (1: 200, Sigma‐Aldrich, Cat# SAB4200497), by the confocal microscope (Zeiss, LSM980). For mouse FLSs, the same markers were used for characterization by flow cytometry (BD Biosciences, FACSCanto SORP).

### 4.4 Apoptosis Assay

RA‐FLSs were seeded into 6‐well culture plates and allowed to adhere for 24 h. After being treated with different treatments in each study. Harvested cells were stained using Annexin V‐FITC Apoptosis Detection Kit (Beyotime, Cat# C1062L) according to the instructions. Flow cytometry was performed by the FACSCanto SORP (BD Biosciences). The data analysis was performed using the FlowJo software [73].

### 4.5 TUNEL Assay

After different treatments in each study, RA‐FLSs were fixed in 4% formalin for 10 min and incubated with Cy3‐labeled dUTP and terminal deoxynucleotidyl transferase enzyme mixture from a One Step TUNEL Apoptosis Assay Kit (Beyotime, Cat# C1086) for 1 h at room temperature. Cell nuclei were counterstained using the DAPI‐containing mounting medium (Beyotime, Cat# P0131), and apoptotic cells were imaged using a confocal fluorescence microscope (Zeiss, LSM980) [74].

### 4.6 Colony Formation Assay

RA‐FLSs were collected and seeded in 6‐well plates. After different treatments in each study, when most single clones (> 50 cells) formed (about 2–3 weeks), the colonies were stained with 0.1% crystal violet (Aladdin, Cat# C110703) for 30 min after being fixed with 4% paraformaldehyde (Beyotime, Cat# P0099) for 20 min [65]. The number of colonies was counted using ImageJ software.

### 4.7 Transwell Assay

Before harvesting and resuspending in DMEM medium without FBS, RA‐FLSs were treated with different treatments. Then, 200 µL serum‐free medium containing RA‐FLSs (5 × 10^3^ cells/well) was added to the top chamber of 24‐well Transwell plates with 8 µm pore polyester membrane (Jet, Cat# TCS013024). Especially in the invasion assay, cells (8 × 10^3^ cells/well) were seeded into the top chamber coated with Matrigel (Corning, Cat# 354234). 600 µL DMEM with 20% FBS was added to the bottom chamber to induce migration or invasion. Cells that had passed through the membrane were stained with 0.1% crystal violet (Aladdin, Cat# C110703) after being fixed with 4% paraformaldehyde (Beyotime, Cat# P0099). Images were acquired using an inverted light microscope (Nikon, ECLIPSE Ts2) [75].

### 4.8 Wound Healing Assay

RA‐FLSs were treated with different treatments in each study and cultured in 6‐well culture plates. Following a confluence of more than 90%, artificial scratches were created using a 200 µl pipette tip. Then, the cells were washed with PBS and cultured in DMEM without FBS. The artificial scratches were photographed using an inverted light microscope (Nikon, ECLIPSE Ts2) and quantified with the ImageJ software. The wound healing rate was calculated [76].

### 4.9 Western Blot Analysis

Total protein extracted from cells or tissues was quantified by a BCA assay (Thermo Scientific, Cat# 23225). Protein samples were separated by sodium dodecyl sulfate‐polyacrylamide gel electrophoresis (SDS‐PAGE) and transferred onto PVDF membranes (Millipore, Cat# IPVH00010). After blocking using 5% skim milk (BD, Cat# 232100), the membrane was incubated overnight at 4°C with primary antibodies (anti‐OGT,1:1000, Abcam, Cat# ab96718; anti‐H3K27me3, 1:1000, Abcam, Cat# ab6002; anti‐H3K9ac, 1:1000, Abcam, Cat# ab32129; anti‐H3K14ac, 1:1000, Abcam, Cat# ab52946; anti‐H3K27ac, 1:1000, Abcam, Cat# ab4729; anti‐H3, 1:30000, Abcam, Cat# ab1791; anti‐RL2, 1:1000, Invitrogen, Cat# MA1‐072; anti‐SAP130, 1:1000, Abcam, Cat# AB111739; anti‐Ubiquitin,1:1000, CST, Cat#58395; anti‐THY1, 1:1000, Sigma‐Aldrich, Cat# SAB4200497; anti‐CD90, 1:1000, Proteintech, Cat# 5246; anti‐TUBULIN, 1:5000, ABclonal, Cat# AC012; anti‐β‐actin, 1:1000, ABclonal, Cat# AC050; anti‐BTG2, 1:1000, Proteintech, Cat# 22339‐1‐AP; anti‐acetyl lysine, 1:1000, Abcam, Cat# ab61257); anti‐MDM2, 1:1000, Proteintech, Cat# 27883‐1‐AP; anti‐NCL, 1:1000, Proteintech, Cat# 10556‐1‐AP; anti‐MYC, 1:5000, ABclonal, Cat# AE070; anti‐Flag, 1:5000, ABclonal, Cat# AE005; anti‐Na/K‐ATPase,1:1000, CST, Cat# 3010S). Then, specific HRP‐conjugated secondary antibodies (1:4000, ABclonal, Cat# AS003/AS014) were used to incubate the membrane. Immunodetection was performed using an enhanced chemiluminescence ECL kit (ABclonal, Cat# RM00021P) through a chemiluminescence imaging system (Tanon, Multi5200).

### 4.10 qRT‐PCR

Total RNA was isolated using the TransZol Up Plus RNA Kit (TransGen, Cat# ER501‐01‐V2). cDNA was synthesized by PrimeScript RT reagent Kit (Takara, Cat# RR037A). Then, the gene expression was measured by the CFX96 Touch instrument (Bio‐Rad) with the Green qPCR SuperMix (TransGen, Cat# AQ601‐01‐V2). Data were normalized to the expression levels of the housekeeping gene GAPDH and expressed as mean ± SEM. Results were calculated as fold change compared to the expression level of each gene in control samples using the comparative threshold cycle (Ct) method with the formula 2^ ^(‐△△Ct)^. The specific primers used are listed in Table S2.

### 4.11 ChIP‐qPCR

A ChIP‐qPCR assay was performed according to the manufacturer's instructions (Active Motif, Cat# 53009). Briefly, RA‐FLSs were collected and cross‐linked with formaldehyde for 10 min at room temperature to prepare chromatin. The crosslinking reaction was stopped by adding glycine, and the chromatin was enzymatically digested to generate 200 ∼ 500 bp DNA fragments. Immunoprecipitation was performed by adding anti‐HDAC1 (3 µg, CST, Cat# 2062), anti‐H3K27me3 (3 µg, Abcam, Cat# ab6002), anti‐H3K27ac (3 µg, Abcam, Cat# ab4729), anti‐IgG (3 µg, Abconal, Cat# AC005), and Protein G magnetic beads to each sample, which were then incubated overnight at 4°C. Following the final elution, cross‐link reversal, and proteinase K digestion of the immunoprecipitated chromatin, the samples should be subjected to a DNA clean‐up step using the Active Motif Chromatin IP DNA Purification Kit. Then, qPCR was performed using specific primers for the *BTG2* gene promoters, as listed in Table S2.

### 4.12 In Vivo Fluorescence Imaging Analysis

Cy5‐labeled NC or Cy5‐labeled AS1411 (Sangon) was intravenously given to the CIA mice via the tail vein. An IVIS Spectrum imaging system (PerkinElmer) was used to examine the fluorescence of NC and AS1411. After the mice's sacrifice, the synovium and hind paws were collected for biphotonic imaging. All images were acquired with an exposure time of 1 s. NC: 5′‐CCTCCTCCTCCTTCTCCTCCTCCTCC‐3′. AS1411: 5′‐GGTGGTGGTGGTTGTGGTGGTGGTGG‐3′ [25].

### 4.13 Co‐IP Assay

After different treatments, the cell lysates or recombinant protein samples were incubated with the corresponding primary antibodies (anti‐OGT, 1: 200, Abcam, Cat# ab96718; anti‐SAP130,1: 200, Proteintech, Cat# 12130‐1‐AP; anti‐MYC, 1:5000, ABclonal, Cat# AE070; anti‐Flag, 1:5000, ABclonal, Cat# AE005) overnight at 4°C and then incubated with Protein A/G Magnetic Beads (Thermo Scientific, Cat# 88802) at room temperature for 2 h. To reduce nonspecific binding, the beads were extensively washed using a DynaMag‐2 Magnet (Thermo Scientific, Cat# 12321D). The samples were heated to 100°C for 10 min with SDS‐PAGE protein loading buffer. The protein samples were subjected to Western blot analysis.

### 4.14 WGA Pull‐Down Assay

The WGA pull‐down assay was performed to enrich O‐GlcNAcylated proteins as previously described [77]. In brief, RA‐FLSs were lysed in lysis buffer (Thermo Scientific, Cat# PI87787), and the cell lysates were incubated with WGA‐coupled agarose beads (Vector Laboratories, Cat# AL‐1023) at 4°C for 4 h. Agarose beads were washed four times with lysis buffer and then heated to 100°C for 10 min with SDS‐PAGE protein loading buffer. The protein samples were subjected to Western blot analysis.

### 4.15 In Vitro O‐GlcNAcylation Assay

The in vitro O‐GlcNAcylation assay was performed as previously described [78]. Briefly, 1 µg of recombinant His‐OGT protein (aa323‐1041) (Creative Biomart, Cat# OGT‐940H) was incubated with 2 µg of recombinant Myc‐SAP130 (Creative Biomart, Cat# SAP130‐2509H) in 60 µL reaction buffer (50 mM Tris‐HCl, 12.5 mM MgCl_2_, 2 mM UDP‐GlcNAc, 1 mM DTT, pH 7.5) at 37°C for 4 h. For O‐GlcNAc removal, O‐GlcNAcylated SAP130 was incubated with 5 µg OGA at 37°C for 4 h in 60 µL reaction buffer. The reaction products were separated by SDS‐PAGE and subjected to Western blot analysis.

### 4.16 Gene Silencing by siRNA

RA‐FLSs were seeded in 6‐well plates and allowed to adhere for 24 h. According to the manufacturer's instructions, the cells were transfected at a final concentration of 20 nM siRNA using Lipofectamine RNAiMAX Reagent (Thermo Fisher, Cat# 13778150). The sequences of siRNA are listed in Table S3.

### 4.17 Construction of Plasmids

The pCDH‐CMV and pCDH‐CMV‐Flag vectors were used to overexpress OGT. The pCS2‐SP6‐MYC and pCS2‐SP6‐HA vectors were used to clone the full‐length SAP130 and its mutations (T281A, S282G, T296A, S316G, and T473A). The *BTG2* gene promoter (‐1∼‐2000 bp) was cloned into pGL3‐Basic to carry out the dual‐luciferase reporter assay. All constructs were verified by Sanger sequencing. Sequences of primers in plasmid construction are shown in Table S4.

### 4.18 EdU Proliferation Assay

Cell proliferation was determined using a BeyoClick EdU Cell Proliferation Kit with Alexa Fluor 488 (Beyotime, Cat# C0071S). Briefly, RA‐FLSs (1 × 10^4^ cells per well) were seeded in confocal plates and allowed to adhere for 24 h. After different treatments in each study, 50 µL of EdU was added to the cells and incubated at 37°C. The cells were fixed in 4% formalin for 10 min, permeabilized with 0.2% Triton X‐100 for 10 min and blocked with 5% BSA in PBS for 1 h. A staining cocktail including 430 µL Click reaction buffer, 50 µL Click additive solution, 1 µL Alexa Fluor 488, and 20 µL CuSO_4_ were added to the cells for 30 min. Finally, the cells were counterstained with DAPI, and images were acquired using a confocal fluorescence microscope [79].

### 4.19 RNA‐Seq

RNA sequencing was conducted by Novogene. The raw sequencing data were first evaluated using FastQC for quality assurance. To obtain high‐quality reads, the data were trimmed using Trimmomatic. These high‐quality reads were then aligned to the reference genome using HISAT2, and mapping statistics were compiled. Subsequently, the featureCounts was integrated into the pipeline to quantify gene expression levels. It quickly and accurately counted the number of reads mapped to each gene or feature, using the aligned read files as input and the gene annotation in GTF format to assign reads to the corresponding genes. Differentially expressed genes (DEGs) between siOGT vs siCtrl groups and siSAP130 vs siCtrl were identified based on criteria of *p*‐value < 0.05 and |log_2_FC| > 1. Finally, for the differential expression analysis, the read counts generated by featureCounts were used to perform hierarchical clustering, volcano plots, and enrichment analysis.

### 4.20 LC‐MS/MS Analysis

A data‐independent acquisition parallel accumulation serial fragmentation (DIA‐PASEF) method based on liquid chromatography‐tandem mass spectrometry was employed for analysis. The tryptic peptides were separated using an Easy‐nLC 1000 ultra‐high performance liquid chromatography system. Mobile phase A consisted of water containing 0.1% formic acid and 2% acetonitrile, while mobile phase B was acetonitrile containing 0.1% formic acid. The peptides were eluted with a gradient from 9% to 24% B over 0–18 min, 24% to 35% B over 18–22 min, 35% to 90% B over 22–26 min, and maintained at 90% B from 26–30 min, at a constant flow rate of 450 nL/min. The eluted peptides were ionized via a capillary source with an electrospray voltage of 1.6 kV and analyzed using a timsTOF Pro mass spectrometer. Mass spectrometry detection was performed in DIA‐PASEF mode with a full MS scan range of 100–1700 m/z and an MS/MS scan range of 425–1025 m/z. The isolation window was set at 25 m/z, with 8 PASEF MS/MS scans acquired per cycle. For database searching, the DDA data were processed using Spectronaut (v17.0) software with the Pulsar search engine, searching against the Homo_sapiens_9606_SP_20230103.fasta database (20 389 entries) concatenated with a reverse decoy database. Carbamidomethylation of cysteine was set as a fixed modification, while acetylation of protein N‐termini, methionine oxidation, and O‐glycosylation were specified as variable modifications. The maximum number of missed cleavages was set to 2, and the false discovery rate for proteins, peptides, and PSMs was adjusted to <1%.

### 4.21 Animals

Male DBA/1J mice aged 6–8 weeks were purchased from the Gempharmatech Co. All transgenic mice were purchased from Cyagen Biosciences Inc. We bred *Ogt* ^fl/fl^ mice with the Col6a1‐CreERT2 mice to generate the inducible *Ogt* cKO mice (*Ogt* ^fl/fl^; Col6a1‐CreERT2 mice). For inducible deletion of *Ogt*, male mice aged 2 months were intraperitoneally administered with 100 mg/kg TAM (MCE, Cat# HY‐13757A) for 5 consecutive days. All mice were housed in the specific pathogen‐free (SPF) mouse facility of the Experimental Animal Center of the Southern University of Science and Technology. The housing conditions were maintained at a constant ambient temperature of 20–22°C, a relative humidity range of 40–60%, a 12‐h light/dark cycle, and continuous ad libitum access. All animal procedures were approved by the Institutional Animal Care and Use Committee at the Southern University of Science and Technology (SUSTech‐JY202201009).

### 4.22 CAIA Mouse Model

The CAIA mouse model was induced following a previously established protocol [80]. 5 mg/mouse of a specific combination of monoclonal antibodies against CII (mAb cocktail) (Chondrex, Cat# 53010) was injected on day 0, followed by an LPS injection (50 µg) on day 3 into the control and cKO mice. Arthritis progresses rapidly, and acute inflammation peaks on day 7–10. The scoring system ranged from 0 to 4: 0 = normal; 1 = mild but definite redness and swelling of the ankles or redness and swelling of any degree in any single digit; 2 = moderate to severe redness and swelling of the ankles; 3 = redness and swelling of the entire foot including the digits; and 4 = maximally inflamed limb, with involvement of multiple joints. A score of 1 or above was considered indicative of arthritis [80].

### 4.23 CIA Mouse Model

The CIA mouse model was induced as previously described [81]. In brief, bovine CII (Chondrex, Cat# GCDD0002) (2 mg/mL) was emulsified with Complete Freund's adjuvant (CFA) (Chondrex, Cat# 7001) (4 mg/mL), followed by subcutaneous injection of 100 µL of the emulsion at the tail of DBA/1J mouse. When the onset of arthritis occurred, its severity was monitored by evaluating clinical arthritis scores. The mouse arthritis scoring was graded from 0–4 as the following shown: sore 0 = normal; sore 1 = one joint has redness and swelling; sore 2 = two joints have redness and swelling; sore 3 = all three joints have redness and swelling; and sore 4 = maximum redness and swelling of the entire paw. The arthritic scores of four paws were summed. A mouse with a score of 1 or above was regarded as arthritic.

### 4.24 Micro‐CT Analysis

The joints were fixed in 10% formalin (Solarbio, Cat# G2161) and then transferred to 70% ethanol. Scanning was performed using a high‐resolution µCT scanner (Bruker, SKYSCAN 1276) with a voxel size of 10 µm, at 60 kVp/100 µAmp. The images were reconstructed to generate three‐dimensional (3D) images using NRecon software and Date Viewer. The µCT data were processed using CTAn software, and images were generated using CTVOX software. Scanned images were processed using the same thresholds to keep the 3D structural rendering of each sample.

### 4.25 HE and SO/FG Staining

After µCT analysis, the hind paws were decalcified for 30 days in 10% EDTA (OKA, Cat# GCDD0002). After dehydration, embedding, and sectioning, the sections were deparaffinized and hydrated using a gradient concentration of alcohol. Then, HE staining (Servicebio, Cat# G1076) and SO/FG staining (Solarbio, Cat# G1371) were performed according to the standard protocol. Finally, the sections were photographed using a digital pathology system (Leica, Aperio GT 450). Histopathological features such as synovial inflammation, bone erosion, and cartilage erosion were scored from 0 to 3, as the following criteria: 0 = healthy, 1 = minor synovial inflammation/bone erosion/cartilage erosion, 2 = moderate synovial inflammation/bone erosion/cartilage erosion, 3 = severe synovial inflammation/bone erosion/cartilage erosion [82, 83].

### 4.26 IF Staining

Tissue sections were deparaffinized with xylene, rehydrated, and processed for antigen retrieval. Cultured cells were seeded on glass coverslips at about 60–70% confluence, fixed with 4% paraformaldehyde for 15 min, and then permeabilized with 0.2% Triton X‐100 (Beyotime, Cat# ST795) for 10 min. After blocking with blocking buffer (Beyotime, Cat# P0260) for 1 h, and incubated with primary antibodies (anti‐OGT, 1:200, Abcam, Cat# ab96718, PTM Bio, Cat# PTM‐5497; anti‐THY1, 1:200, Sigma‐Aldrich, Cat# SAB4200497; anti‐RL2, 1:200, Invitrogen, Cat# MA1‐072; anti‐CXCL12, 1:200, Proteintech, Cat# 17402‐1‐AP; anti‐JAK2, 1:200, Epizyme, Cat# R013499; anti‐SAP130, 1:200, Abcam, Cat# AB111739; anti‐BTG2, 1:200, Proteintech, Cat# 22339‐1‐AP; anti‐Mouse CD45R (B220), 1:200, Proteintech, Cat# 65139‐1) at 4°C overnight. After washing, the sections were incubated with anti‐mouse Alexa Fluor 488 (Abcam, Cat# ab150113) or anti‐rabbit Alexa Fluor 488 (Abcam, Cat# ab150077) for 1 h at room temperature. Finally, the sections were counterstained with DAPI (Abcam, Cat# ab104139), and images were acquired using a confocal fluorescence microscope (Zeiss, LSM980) [83].

### 4.27 Statistical Analysis

All data are presented as mean ± SD. For comparisons between two independent groups, continuous variables were analyzed using an unpaired t‐test. Comparisons among three or more independent groups were performed using oneway ANOVA followed by Dunnett's test or Tukey's test. Two‑way ANOVA followed by Tukey's test was used to evaluate differences between groups stratified by two independent variables. For analysis of human sample data, non‑parametric tests were applied [84]. Specifically, the Mann‑Whitney U test was used for comparisons between two independent groups. All statistical analyses were conducted using SPSS software (version 29.0). *P* < 0.05 is considered statistically significant.

## Author Contributions

C.L. and A.L. conceived and designed the study. D.X. and J.H. performed animal and cell experiments, wrote and revised the manuscript. C.L., B.H., and J.Y. revised the manuscript. C.C., Z.T., B.H., and F.W. performed bioinformatics analysis. X.L., J.G., and X.C. reviewed the relevant literature, and Y.D., D.H., and J.L. collected patient samples.

## Funding

This work is supported by the Shenzhen Medical Research Fund (B2502016), the National Key R&D Program of China (2024YFC3506200 and 2024YFC3506205), the National Natural Science Foundation Council of China (82472394 and 82673069), the Shenzhen Science and Technology Program (JCYJ20210324104201005 and SGDX20240115112400001), the Hong Kong General Research Fund (12102722, 12104825, and 12106424), the Hong Kong RGC Theme‐based Research Scheme (T12‐201/20‐R), and the Shenzhen LingGene Biotech Co. Ltd.

## Conflicts of Interest

The authors declare the following competing financial interest(s): A patent application has been filed related to this work by the Shenzhen LingGene Biotech Co., Ltd.

## Supporting information




**Supporting File**: advs77466‐sup‐0001‐SuppMat.pdf.

## Data Availability

The data that support the findings of this study are available from the corresponding author upon reasonable request. The data are not publicly available due to privacy or ethical restrictions.
