# Supplementary material for: Multi‐Technique Integration Identifies O‐GlcNAc Transferase in Fibroblast‐Like Synoviocytes as a Therapeutic Target for Rheumatoid Arthritis
Source: Adv Sci (Weinh). 2026 Aug 31:e77466. Online ahead of print. doi: 10.1002/advs.77466 (PMC13528181; doi:10.1002/advs.77466)
Supplement: Supplementary file 1 [file ADVS-9999-e77466-s001.pdf]

Supplementary Materials for

Multi-Technique Integration Identifies OGT in Fibroblast-Like Synoviocytes as a Therapeutic Target for Rheumatoid Arthritis

Duoli Xie[1, 2#], Jie Huang[1, 2#], Chunhao Cao[1, 2#], Zhenchao Tang[3#], Xingchen Li[1], Jianmin Guo[1, 2], Xinxin Chen[1], Fang Wang[3], Dongyi He[4], Jie Li[5], Yu Du[6*], Bing He[3*], Jianhua Yao[3*], Aiping Lu[2, 7*], Chao Liang[1, 2, 8*]

[1]Department of Systems Biology, School of Life Sciences, Southern University of Science and Technology, Shenzhen, 518055, China.

[2]Institute of Integrated Bioinformedicine and Translational Science (IBTS), School of Chinese Medicine, Hong Kong Baptist University, Hong Kong SAR, 999077, China.

[3]AI for Life Sciences Lab, Tencent, Shenzhen, 518000, China.

[4]Department of Rheumatology, Guanghua Hospital Affiliated to Shanghai University of Traditional Chinese Medicine, Shanghai University of Traditional Chinese Medicine, Shanghai, 201203, China.

[5]Department of Laboratory Medicine, Peking University Shenzhen Hospital, Shenzhen, 518055, China.

[6]Department of Orthopedics, The Second Affiliated Hospital of Chongqing Medical University, Chongqing Medical University, Chongqing, 400010, China.

[7]Shanghai University of Traditional Chinese Medicine, Shanghai, 200032, China.

[8]State Key Laboratory of Proteomics, National Center for Protein Sciences (Beijing), Beijing Institute of Lifeomics, Beijing, 100850, China.

#These authors contributed equally to this study.

**Correspondence: Yu Du, duyu@hospital.cqmu.edu.cn; Bing He, owenbhe@tencent.com; Jianhua Yao, jianhuayao@tencent.com; Aiping Lu, aipinglu@hkbu.edu.hk, Chao Liang, liangc@sustech.edu.cn*

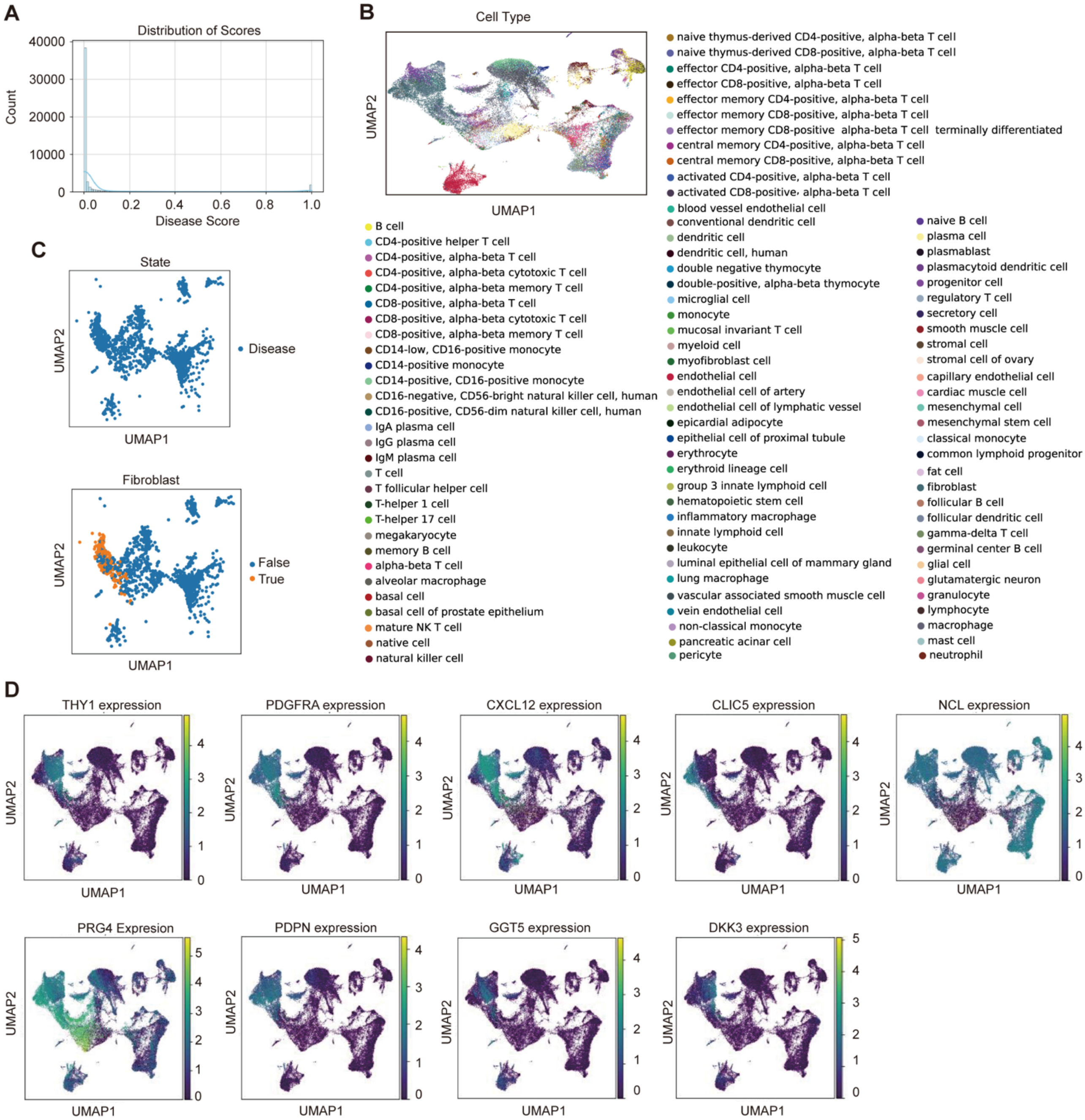

Fig. S1. A canonical FLS signature of fibroblasts with high *OGT* expression. (A) Distribution of disease scores at single-cell resolution inferred by scTransMIL. n = 79. **(B)** UMAP of zero-shot annotation using SCimilarity. n = 79. **(C)** UMAP of fibroblast cells among disease cells. n = 79. (**D)** UMAP of *THY1*, *PDGFRA*, *CXCL12*, *CLIC5*, *NCL*, *PRG4*, *PDPN*, *GGT5*, and *DKK3* expression on the single-cell atlas. n = 79.

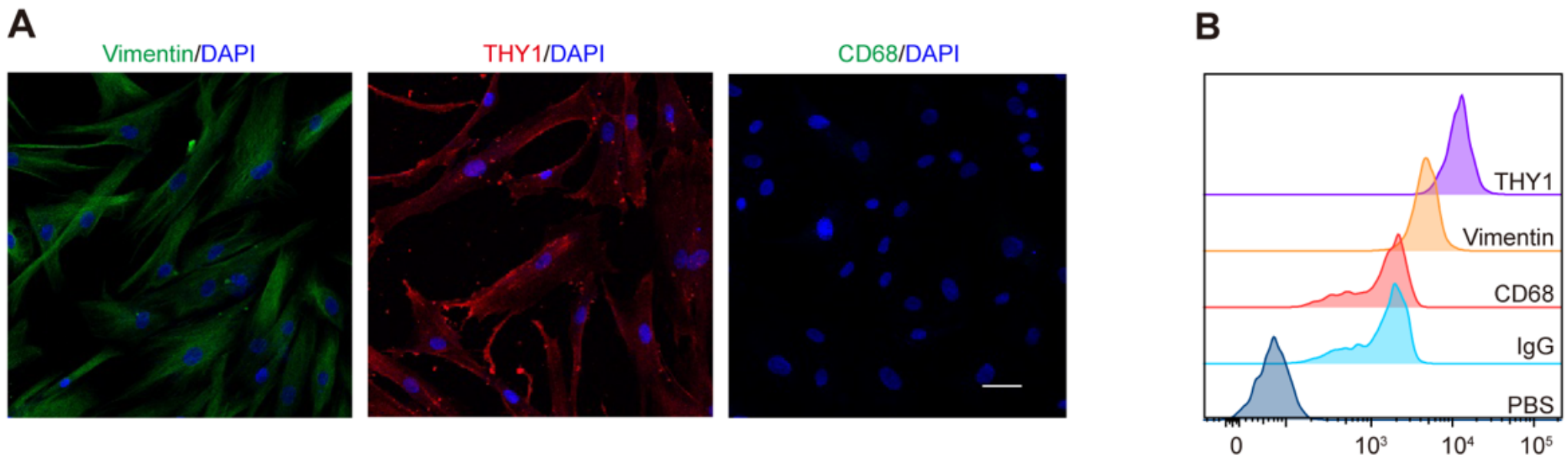

Fig. S2. Characterization of RA-FLSs and mouse FLSs. (A) IF staining of fibroblastic markers (vimentin and THY1) and a macrophage marker CD68 for RA-FLSs. n = 3. Scale bars, 50 μm. **(B)** Flow cytometry analysis of fibroblastic markers (vimentin and THY1) and a macrophage marker CD68 for mouse FLSs. PBS and IgG isotypes were used as negative controls to detect the non-specific background signal. n = 3.

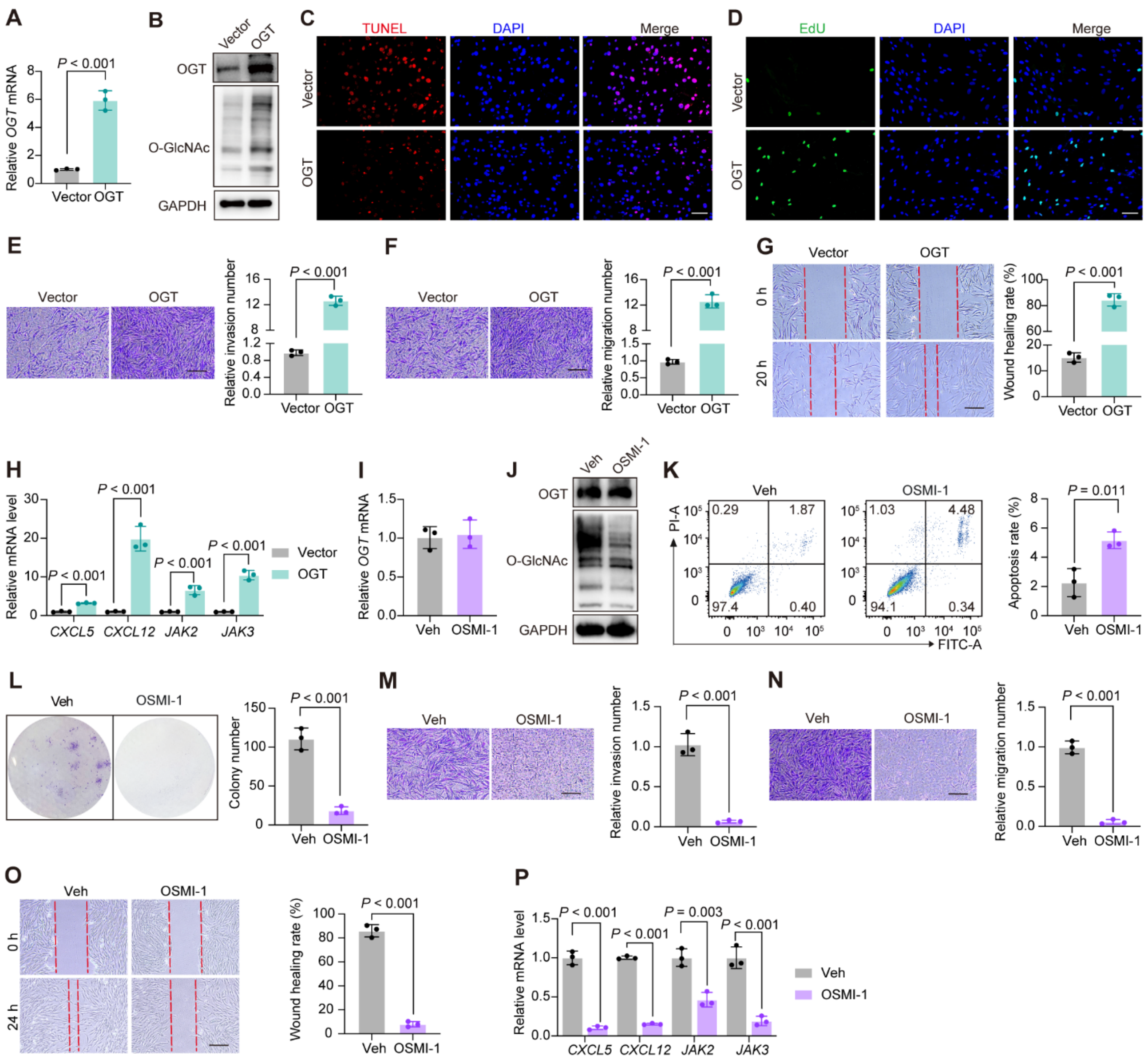

Fig. S3. Effects of OGT overexpression and inhibition on RA-FLSs. (A) *OGT* mRNA levels in RA-FLSs after transfection with the empty vector or the OGT-overexpressing vector. n = 3. **(B)** OGT and O-GlcNAcylation levels in RA-FLSs after transfection with the empty vector or the OGT-overexpressing vector. n = 3. **(C)** Apoptosis of RA-FLSs after transfection with the empty vector or the OGT-overexpressing vector, as measured by TUNEL staining. n = 3. Scale bars, 100 μm. **(D)** Proliferation of RA-FLSs after transfection with the empty vector or the OGT-overexpressing vector, as measured by EdU staining. n = 3. Scale bars, 100 μm. **(E)** Transwell invasion of RA-FLSs after transfection with the empty vector or the OGT-overexpressing vector. Quantification is shown on the right. n = 3. Scale bars, 50 μm. **(F)** Transwell migration of RA-FLSs after transfection with the empty vector or the OGT-overexpressing vector. Quantification is shown on the right. n = 3. Scale bars, 50 μm. **(G)** Wound healing assays of RA-FLSs after transfection with the empty vector or the OGT-overexpressing vector. Quantification is shown on the right. n = 3. Scale bars, 50 μm. **(H)** mRNA expression of *CXCL5*, *CXCL12*, *JAK2*, and *JAK3* in RA-FLSs after transfection with the empty vector or the OGT-overexpressing vector. n = 3. **(I)** *OGT* mRNA levels in RA-FLSs after treatment with Veh or OSMI-1 at a concentration of 40 μM. n = 3. **(J)** OGT and O-GlcNAcylation levels in RA-FLSs after treatment with Veh or OSMI-1 at a concentration of 40 μM. n = 3. **(K)** Apoptosis of RA-FLSs after treatment with Veh or OSMI-1 at a concentration of 40 μM, as determined by flow cytometry. Quantification is shown on the right. n = 3. **(L)** Colony

formation of RA-FLSs after treatment with Veh or OSMI-1 at a concentration of 40 μM. Quantification is shown on the right. n = 3. **(M)** Transwell invasion of RA-FLSs after treatment with Veh or OSMI-1 at a concentration of 40 μM. Quantification is shown on the right. n = 3. Scale bars, 50 μm. **(N)** Transwell migration of RA-FLSs after treatment with Veh or OSMI-1 at a concentration of 40 μM. Quantification is shown on the right. n = 3. Scale bars, 50 μm. **(O)** Wound healing assays of RA-FLSs after treatment with Veh or OSMI-1 at a concentration of 40 μM. Quantification is shown on the right. n = 3. Scale bars, 50 μm. **(P)** mRNA expression of *CXCL5*, *CXCL12*, *JAK2*, and *JAK3* in RA-FLSs after treatment with Veh or OSMI-1 at a concentration of 40 μM. n = 3. Data are presented as mean ± SD, and statistical significance is calculated by unpaired *t*-test (A, E-I, and K-P).

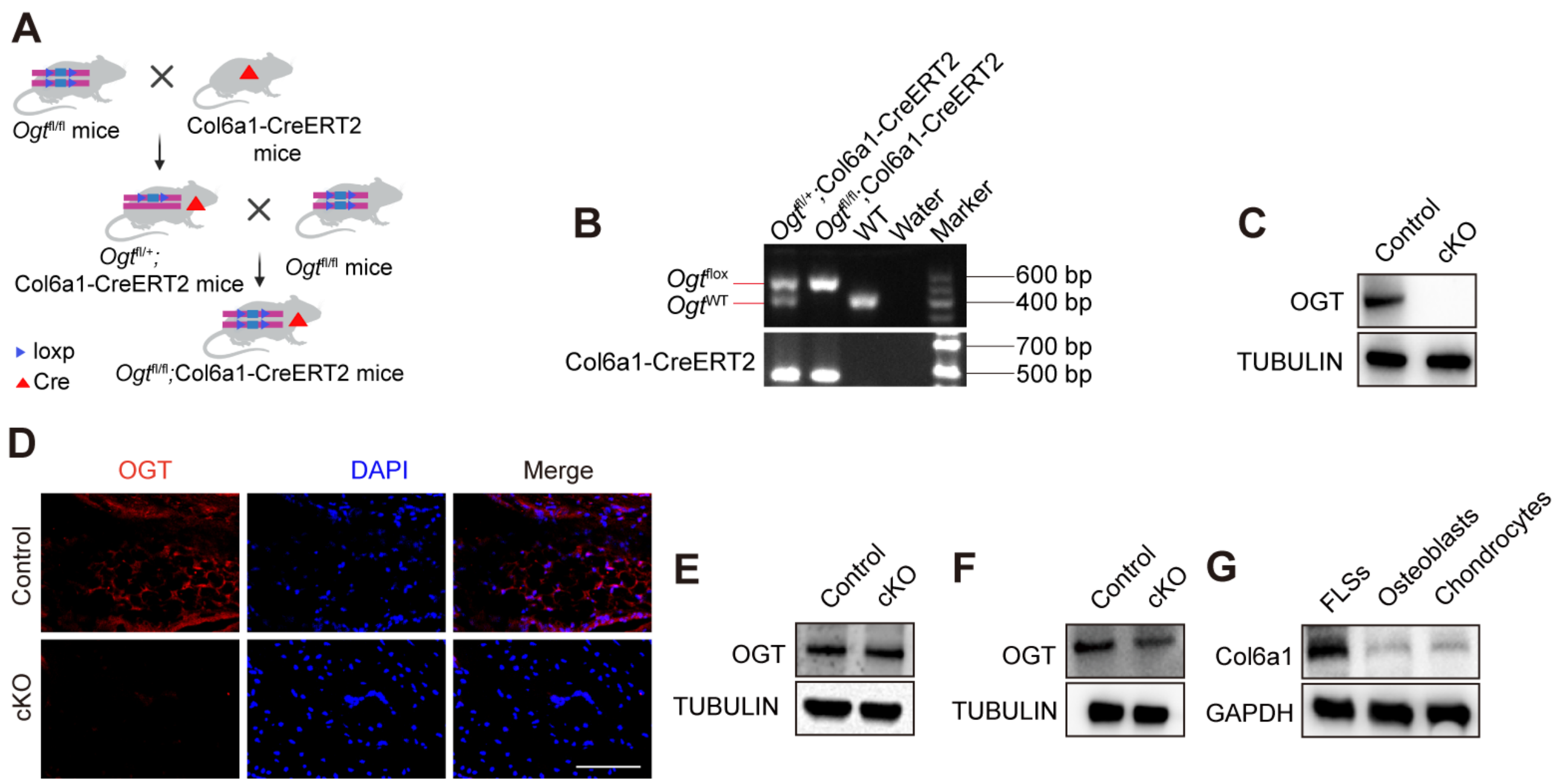

Fig. S4. Generation and characterization of FLS-specific *Ogt* cKO mice. (A) Breeding strategy of *Ogt*$^{fl/fl}$; Col6a1-CreERT2 mice. **(B)** Genotyping PCR verification of *Ogt*$^{fl/+}$; Col6a1-CreERT2 mice, *Ogt*$^{fl/fl}$; Col6a1-CreERT2 mice, and WT mice using tail DNA. n = 3. **(C)** Western blot analysis of OGT in synovial tissues of control and cKO mice. n = 3. **(D)** IF staining of OGT expression on synovial sections of control and cKO mice. n = 3. Scale bar = 100 μm. **(E)** Western blot analysis of OGT in osteoblasts of control and cKO mice. n = 3. **(F)** Western blot analysis of OGT in chondrocytes of control and cKO mice. n = 3. **(G)** Western blot analysis of Col6a1 in FLSs, osteoblasts, and chondrocytes of cKO mice. n = 3.

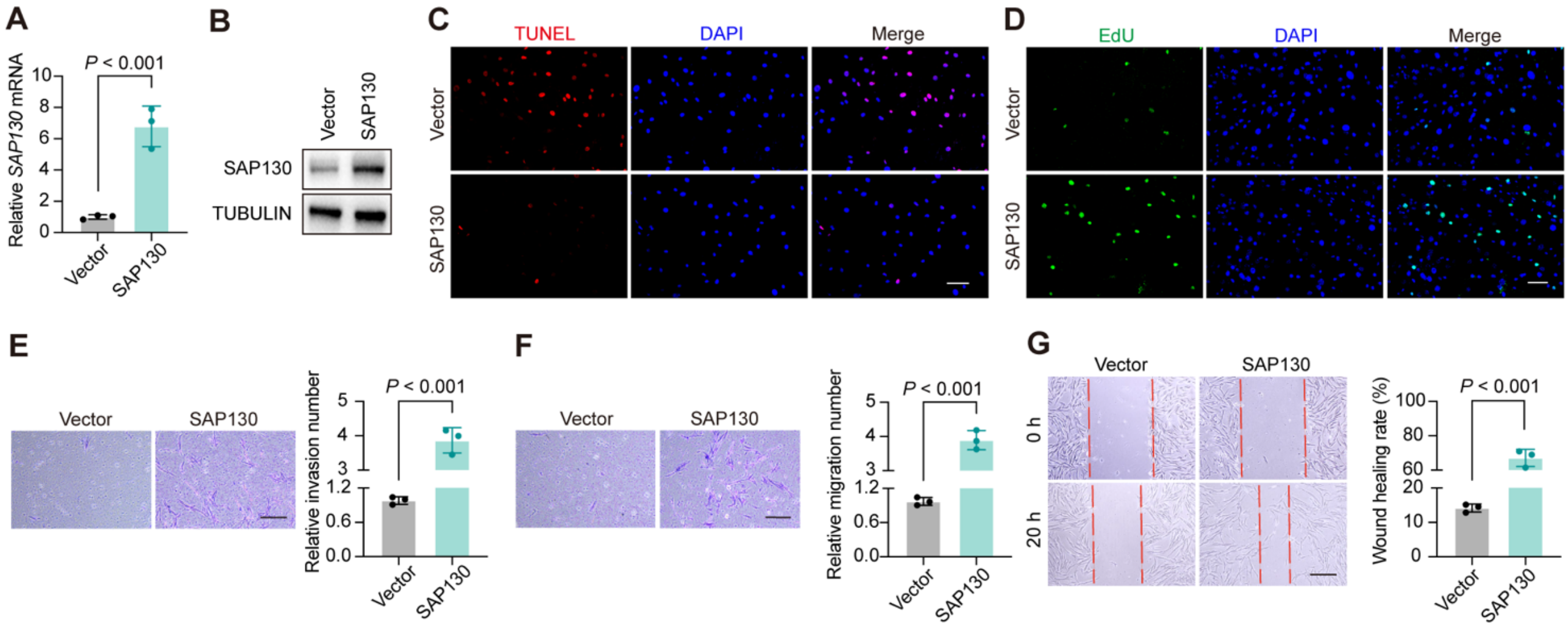

Fig. S5. Effect of SAP130 overexpression on phenotypes of RA-FLSs. (A and B) mRNA **(A)** and protein levels **(B)** of SAP130 in RA-FLSs after transfection with the empty vector or the SAP130-overexpressing vector. n = 3. **(C)** Apoptosis of RA-FLSs after transfection with the empty vector or the SAP130-overexpressing vector, as measured by TUNEL staining. n = 3. Scale bars, 100 μm. **(D)** Proliferation of RA-FLSs after transfection with the empty vector or the SAP130-overexpressing vector, as measured by EdU staining. n = 3. Scale bars, 100 μm. **(E)** Transwell invasion of RA-FLSs after transfection with the empty vector or the SAP130-overexpressing vector. Quantification is shown on the right. n = 3. Scale bars, 50 μm. **(F)** Transwell migration of RA-FLSs after transfection with the empty vector or the SAP130-overexpressing vector. Quantification is shown on the right. n = 3. Scale bars, 50 μm. **(G)** Wound healing assays of RA-FLSs after transfection with the empty vector or the SAP130-overexpressing vector. Quantification is shown on the right. n = 3. Scale bars, 50 μm. Data are presented as mean ± SD, and statistical significance is calculated by unpaired *t*-test (A and E-G).

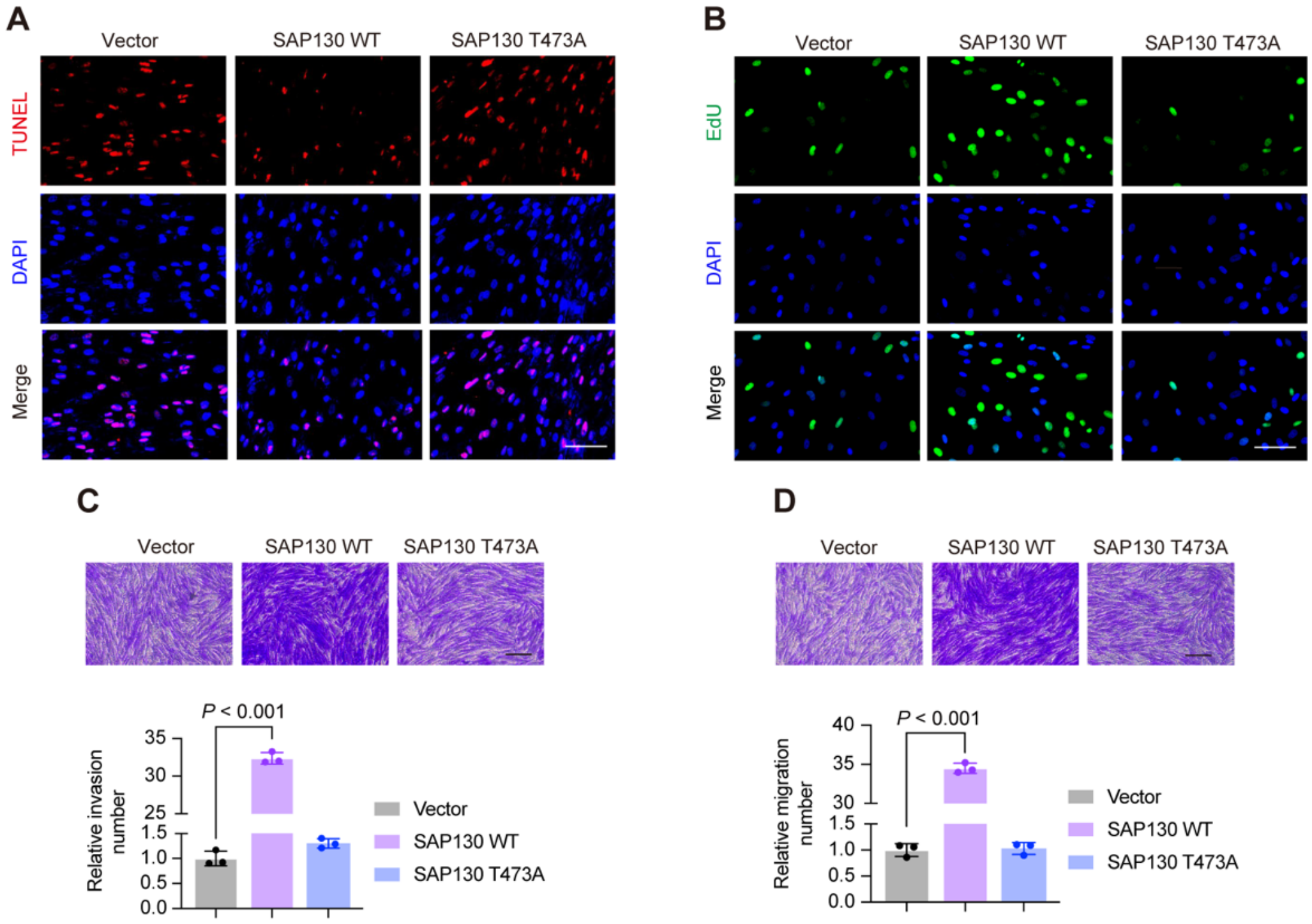

Fig. S6. Effect of SAP130 T473A overexpression on phenotypes of RA-FLSs. (A) Apoptosis of RA-FLSs after transfection with the empty vector, the SAP130-overexpressing vector, or the SAP130 T473A-overexpressing vector, as measured by TUNEL staining. n = 3. Scale bars, 100 μm. **(B)** Proliferation of RA-FLSs after transfection with the empty vector, the SAP130-overexpressing vector, or the SAP130 T473A-overexpressing vector, as measured by EdU staining. n = 3. Scale bars, 100 μm. **(C)** Transwell invasion of RA-FLSs after transfection with the empty vector, the SAP130-overexpressing vector, or the SAP130 T473A-overexpressing vector. Quantification is shown on the bottom. n = 3. Scale bars, 50 μm. **(D)** Transwell migration of RA-FLSs after transfection with the empty vector, the SAP130-overexpressing vector, or the SAP130 T473A-overexpressing vector. Quantification is shown on the bottom. n = 3. Scale bars, 50 μm. Data are presented as mean ± SD, and statistical significance is calculated by one-way ANOVA with Tukey's test (C and D).

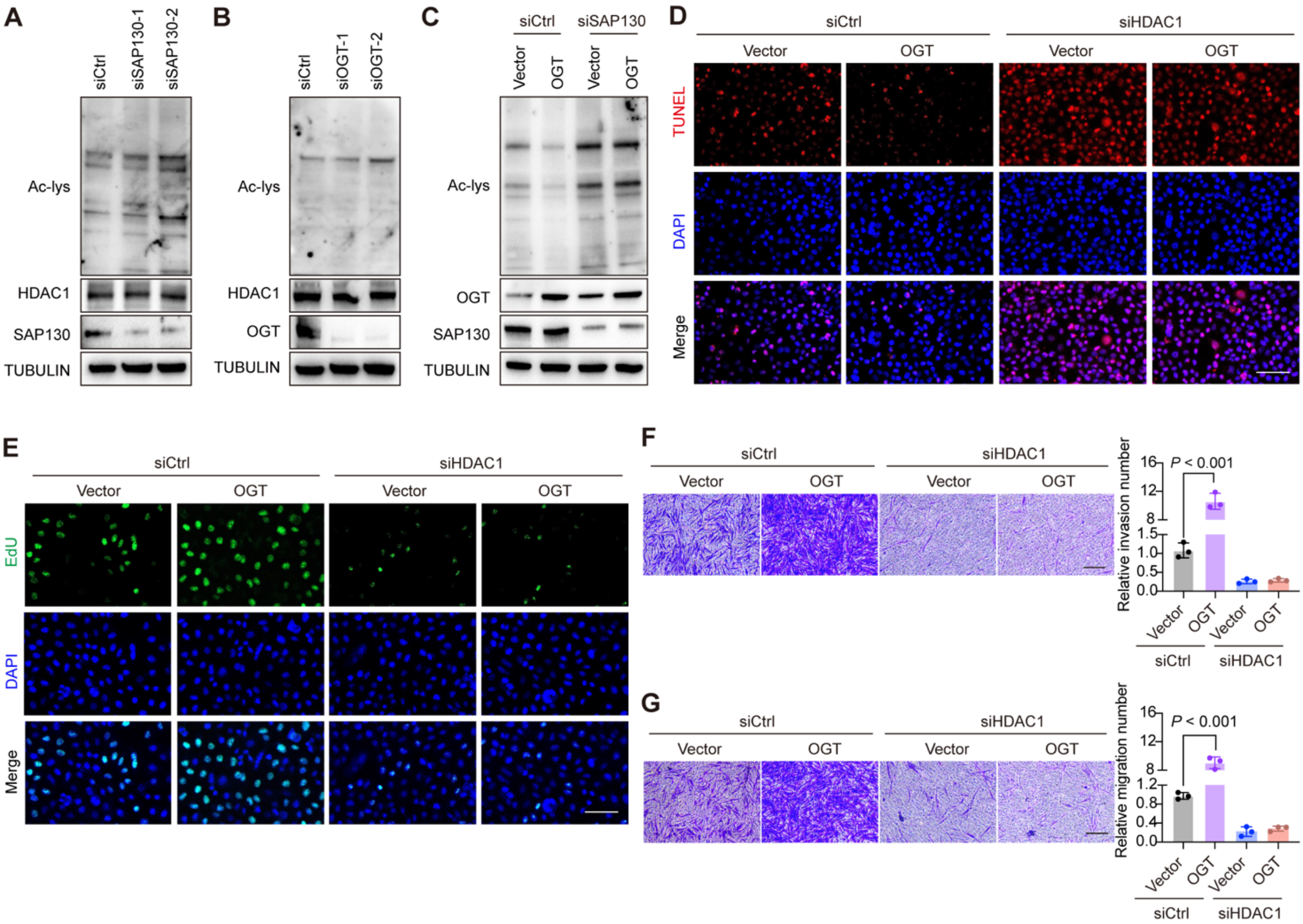

Fig. S7. Effects of OGT overexpression on phenotypes of HDAC1-silenced RA-FLSs. (A) Acetylation levels in RA-FLSs after transfection with siCtrl, siSAP130-1, or siSAP130-2. n = 3. **(B)** Acetylation levels in RA-FLSs after transfection with siCtrl, siOGT-1, or siOGT-2. n = 3. **(C)** Acetylation levels in SAP130-silenced RA-FLSs after transfection with the empty vector or the OGT-overexpressing vector. n = 3. **(D)** Apoptosis of HDAC1-silenced RA-FLSs after transfection with the empty vector or the OGT-overexpressing vector, as measured by TUNEL staining. n = 3. Scale bars = 100 μm. **(E)** Proliferation of HDAC1-silenced RA-FLSs after transfection with the empty vector or the OGT-overexpressing vector, as measured by EdU staining. n = 3. Scale bars = 100 μm. **(F)** Transwell invasion of HDAC1-silenced RA-FLSs after transfection with the empty vector or the OGT-overexpressing vector. Quantification is shown on the right. n = 3. Scale bars, 50 μm. **(G)** Transwell migration of HDAC1-silenced RA-FLSs after transfection with the empty vector or the OGT-overexpressing vector. Quantification is shown on the right. n = 3. Scale bars, 50 μm. Data are presented as mean ± SD, and statistical significance is calculated by one-way ANOVA with Tukey’s test (F and G).

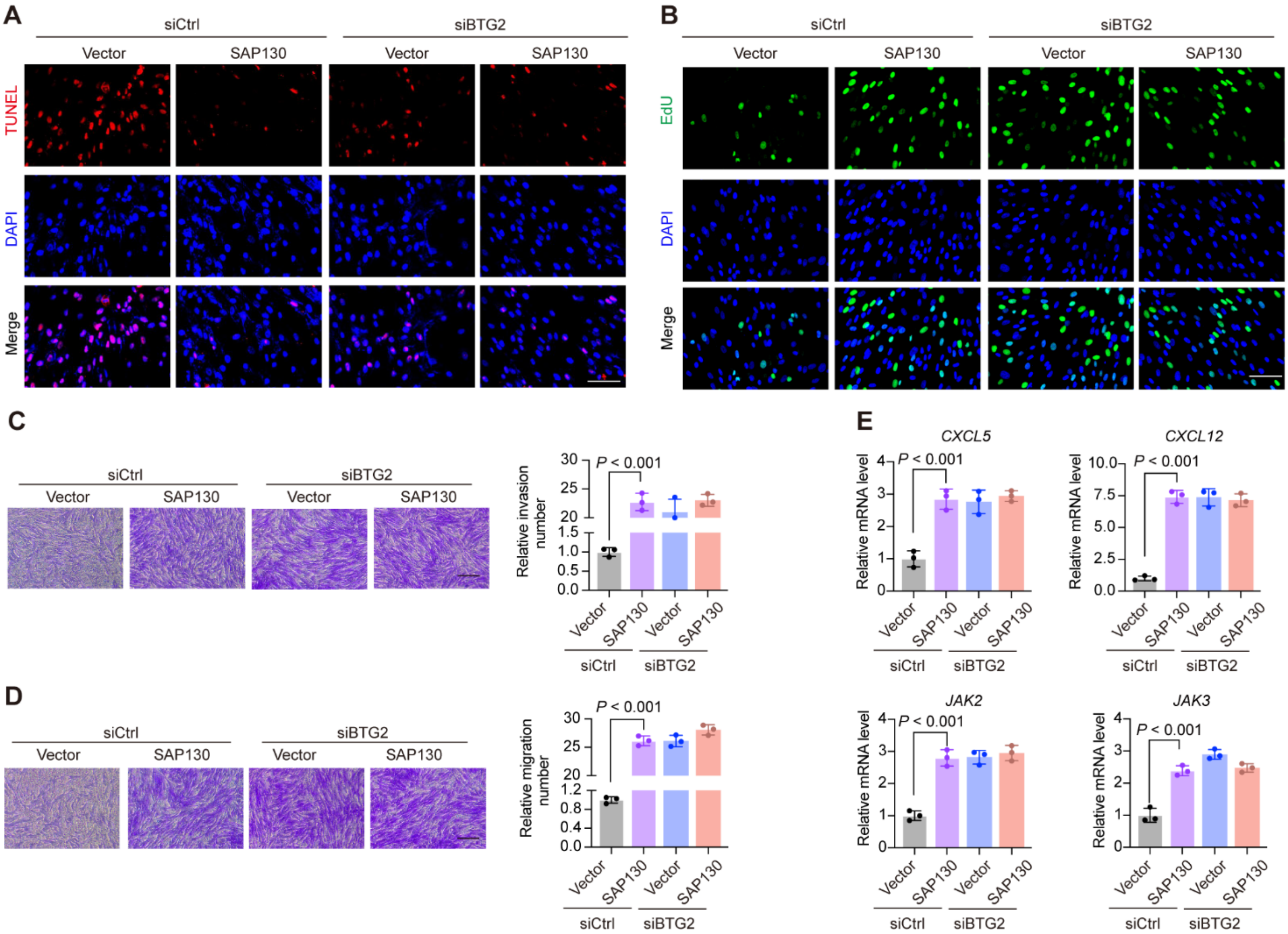

Fig. S8. Effects of SAP130 overexpression on phenotypes of BTG2-silenced RA-FLSs. (A) Apoptosis of BTG2-silenced RA-FLSs after transfection with the empty vector or the SAP130-overexpressing vector, as measured by TUNEL staining. n = 3. Scale bars = 100 μm. **(B)** Proliferation of BTG2-silenced RA-FLSs after transfection with the empty vector or the SAP130-overexpressing vector, as measured by EdU staining. n = 3. Scale bars = 100 μm. **(C)** Transwell invasion of BTG2-silenced RA-FLSs after transfection with the empty vector or the SAP130-overexpressing vector. Quantification is shown on the right. n = 3. Scale bars, 50 μm. **(D)** Transwell migration of BTG2-silenced RA-FLSs after transfection with the empty vector or the SAP130-overexpressing vector. Quantification is shown on the right. n = 3. Scale bars, 50 μm. **(E)** mRNA expression of *CXCL5*, *CXCL12*, *JAK2*, and *JAK3* in BTG2-silenced RA-FLSs after transfection with the empty vector or the SAP130-overexpressing vector. Data are presented as mean ± SD, and statistical significance is calculated by one-way ANOVA with Tukey's test (C-E).

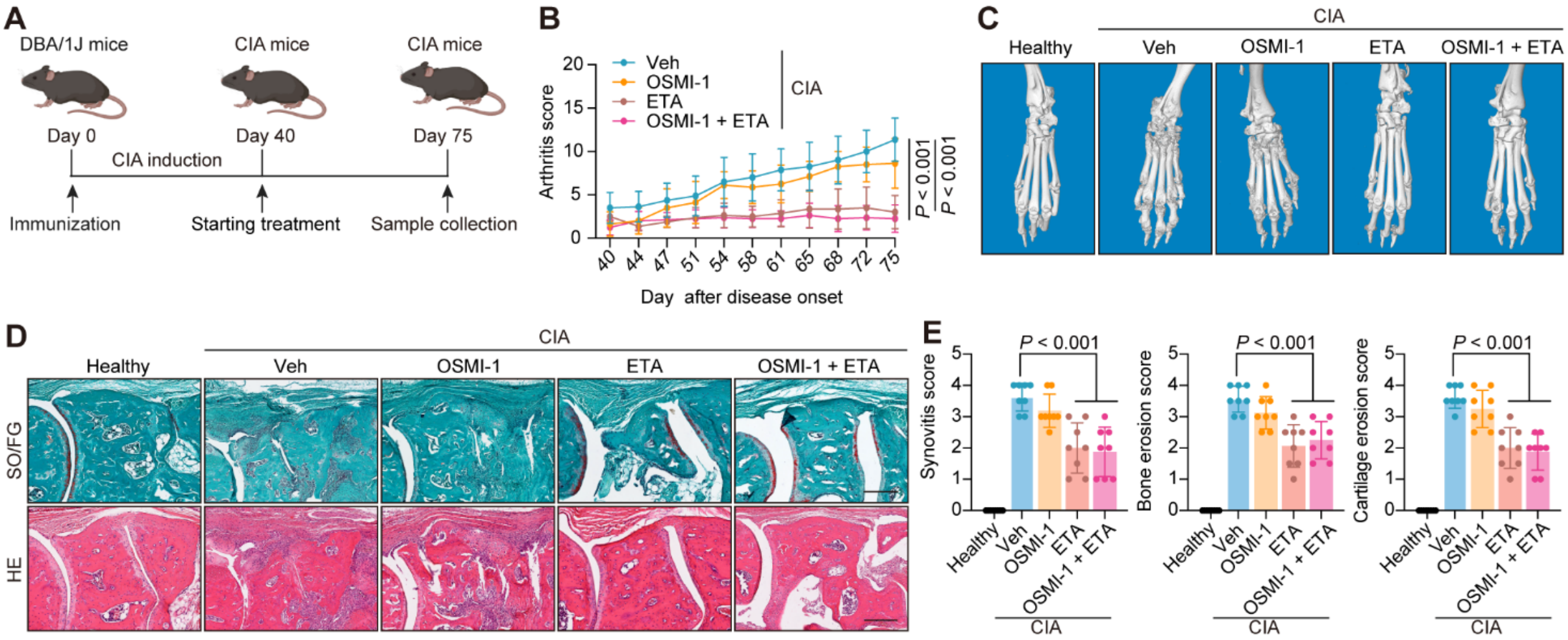

Fig. S9. Therapeutic effects of OSMI-1 in CIA mice. (A) Experimental design for CIA mouse models. DBA/1J mice were immunized with CII emulsified in CFA to induce CIA. Following CIA establishment, CIA mice received systemic treatments with Veh, OSMI-1, ETA, or OSMI-1 + ETA for 35 days. OSMI-1 was intravenously injected at a dosage of 2.5 μmol/kg, and ETA was intraperitoneally administered at a dosage of 5 mg/kg. Non-immunized mice served as healthy controls. **(B)** Arthritis scores of CIA mice from different treatment groups after disease onset. n = 8. **(C)** Micro-CT images of the hind paws of healthy controls or CIA mice from different treatment groups. n = 8. **(D)** Representative images of SO/FG and HE staining on sections of the hind paws of healthy controls or CIA mice from different treatment groups. n = 8. Scale bars, 200 μm. **(E)** Quantification of synovitis, bone, and cartilage erosion on SO/FG- and HE-stained sections. n = 8. Data are presented as mean ± SD, and statistical significance is calculated by two-way ANOVA (B) and one-way ANOVA with Tukey's test (E).

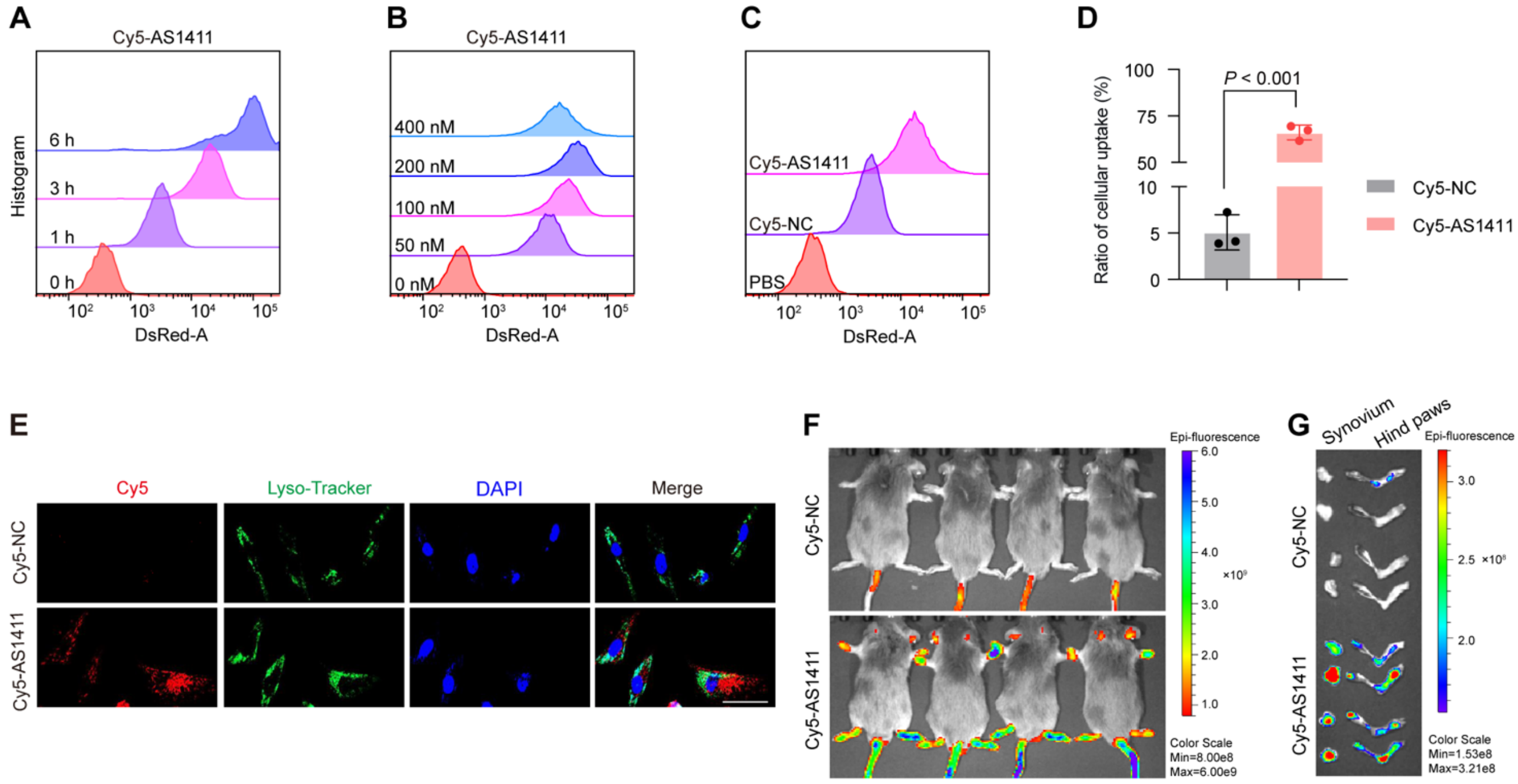

Fig. S10. *In vitro* binding, internalization, and intracellular trafficking, and *in vivo* biodistribution of AS1411. (A) Flow cytometry analysis of the binding of 200 nM Cy5-labeled AS1411 to RA-FLSs at the indicated time points. n = 3. **(B)** Binding of Cy5-labeled AS1411 to RA-FLSs after 3 h of incubation at the indicated concentrations. n = 3. **(C)** Binding of 200 nM Cy5-labeled NC or Cy5-labeled AS1411 to RA-FLSs after 3 h of incubation. n = 3. **(D)** Internalization of 200 nM Cy5-labeled AS1411 into RA-FLSs after 9 h of incubation. n = 3. **(E)** Colocalization of 200 nM Cy5-labeled AS1411 with lysosomal markers in RA-FLSs after 6 h of incubation. n = 3. Scale bars, 50 μm. **(F)** Whole-body *in vivo* imaging of CIA mice at 24 h after intravenous injection of 10 nmol Cy5-labeled NC or Cy5-labeled AS1411. n = 4. **(G)** *In vivo* imaging of synovium and hind paws from CIA mice at 24 h after injection of Cy5-labeled NC or Cy5-labeled AS1411. n = 4. Data are presented as mean ± SD, and statistical significance is calculated by unpaired *t*-test (D).

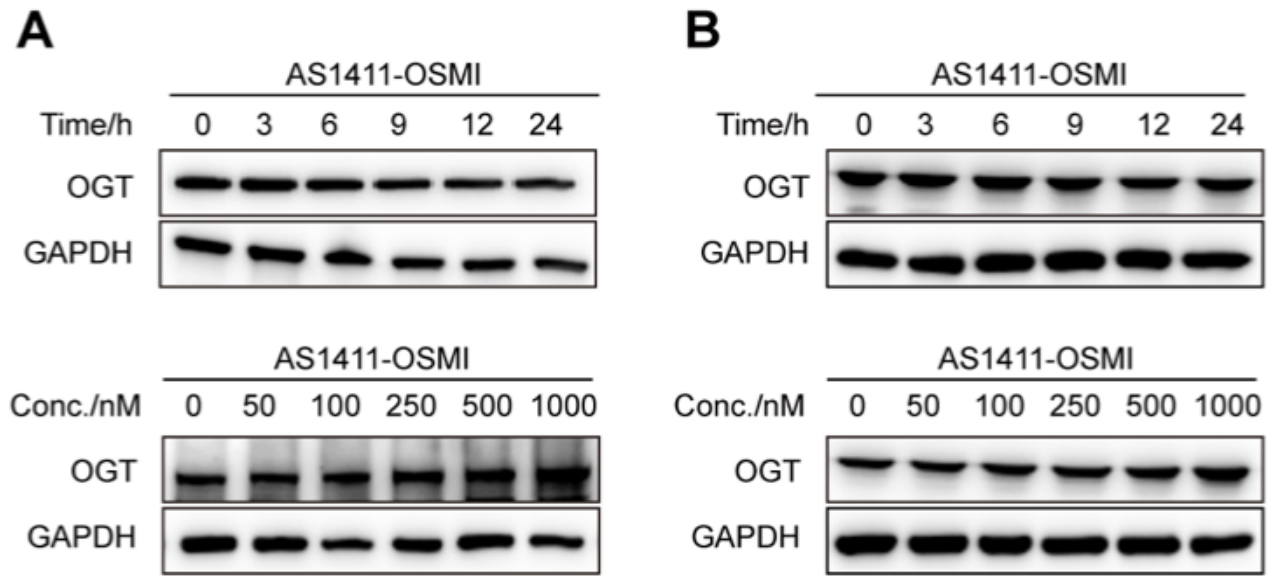

Fig. S11. No degradation of OGT by AS1411-OSMI in non-FLS cells. (A) OGT protein levels in ATDC5 chondroprogenitor cells after treatment with AS1411-OSMI at the indicated time points and concentrations. n = 3. **(B)** OGT protein levels in MC3T3-E1 preosteoblasts after treatment with AS1411-OSMI at the indicated time points and concentrations. n = 3.

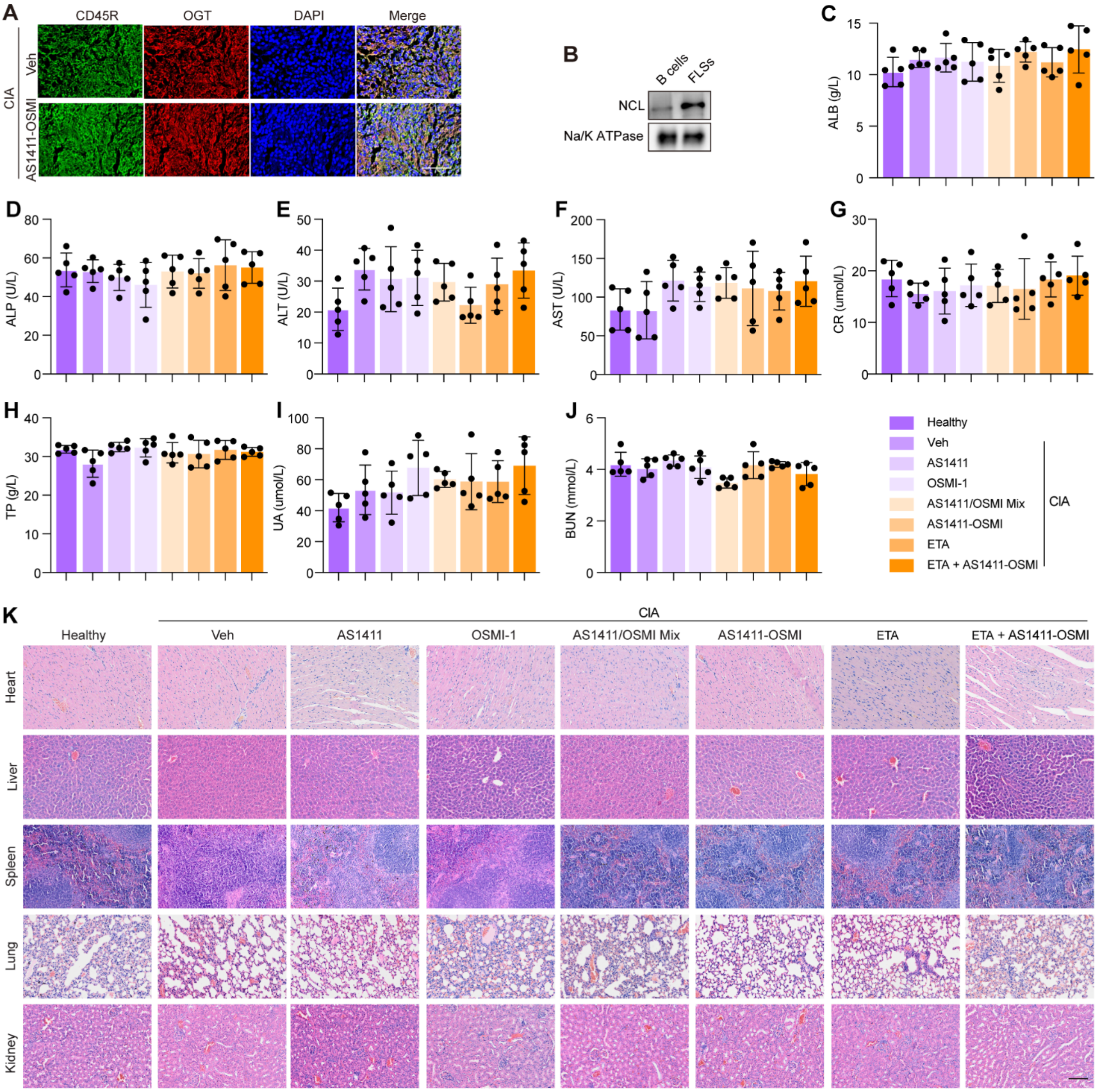

Fig. S12. Safety of AS1411-OSMI in CIA mice. (A) IF staining of OGT and CD45R on sections of spleens from AS1411-OSMI-treated and Veh-treated CIA mice. n = 5. Scale bars, 50 μm. **(B)** Level of surface NCL expression on FLSs and B cells isolated from AS1411-OSMI-treated mice. n = 5. **(C-J)** Serum levels of ALB **(C)**, ALP **(D)**, ALT **(E)**, AST **(F)**, TP **(G)**, CR **(H)**, UA **(I)**, and BUN **(J)** in healthy controls and CIA mice from different treatment groups, as measured by an automated biochemistry analyzer. n = 5. **(K)** Histological analysis of major organs, including the heart, liver, spleen, lung, and kidney, in healthy controls and CIA mice from different treatment groups. n = 5. Scale bars, 200 μm. Data are presented as mean ± SD, and statistical significance is calculated by one-way ANOVA with Tukey's test (C-J).

Table S1 Basic information about clinical samples

Samples	Age	Sex	ESR (mm/h)	CRP (mg/L)
		Healthy vs RA		
Healthy-1	28	Male	/	/
Healthy-2	20	Male	/	/
Healthy-3	30	Male	/	/
RA-1	72	Male	70	64.46
RA-2	40	Female	57	116.87
RA-3	67	Female	29	20.7
		OA VS RA		
OA-1	63	Female	15	2.18
OA-2	76	Male	21	12.91
OA-3	76	Male	18	2.09
OA-4	72	Male	8	1.07
OA-5	78	Male	6	2.01
OA-6	74	Female	5	7.18
OA-7	64	Female	20	6.84
OA-8	69	Female	20	14.45
OA-9	62	Female	6	2.25
OA-10	69	Female	16	1.32
RA-1	72	Male	70	64.46
RA-2	40	Female	57	116.87
RA-3	67	Female	29	20.7
RA-4	62	Female	35	27.58
RA-5	67	Female	16	3.9
RA-6	44	Female	24	6.22
RA-7	78	Female	32	3.07
RA-8	55	Male	12	14.18
RA-9	78	Female	12	6.35

RA-10	72	Female	13	3.08

Table S2 Primers for qRT-PCR and ChIP-qPCR

Genes	Species	Forward (5'-3')	Reverse (5'-3')
For qRT-PCR			
OGT	Human	CATCGAGAATATCAGGCAGGAG	CCTTCGACACTGGAAGTGTATAG
CXCL5	Human	CAGACCACGCAAGGAGTTCA	TCTTCAGGGAGGCTACCACT
CXCL12	Human	TGCCCTTCAGATTGTAGCCC	CTGTAAGGG TTCCTCAGGCG
JAK2	Human	GGATGTGAGTGGGAGCTGAG	CCTGCTTCTGAAACCCGGC
JAK3	Human	CTGCCCGCTAGGCAAGTT	CCAGAGCAAAGAGGGAGTGG
SAP130	Human	AACAGTTTCCTCGGTTAGGAGC	TGGGTTTATCAATCCAGCAGAAC
GAPDH	Human	GGAGCGAGATCCCTCCAAAAT	GGCTGTTGTCATACTTCTCATGG
Ogt	Mouse	CGGCTTGTGTTGTTTCGGTC	GGGCGGCACAGAATACTGAA
Gapdh	Mouse	AGGTCGGTGTGAACGGATTTG	TGTAGACCATGTAGTTGAGGTCA
For ChIP-qPCR			
BTG2 promoter	Human	TTCAGAGCTCTCAGTCCCGT	GCTGTCGTCAGTGCTAGGAA

Table S3 Sequences of siRNAs

Genes	Sequences (5'-3')
siCtrl	UUCUCCGAACGUGUCACGUTT
siOGT-1	GGAUGCUUAUAUCAAUUUAGGTT
siOGT-2	GCAGAAGAUUGUUAUAAUACATT
siSAP130-1	GCUGUGAUGUCCAGUUCUATT
siSAP130-2	CGAGAUGUCACAACAAGAATT
siHDAC1	GCUUCAAUCUAACUAUCAATT
siBTG2	GCAUUCGCAUCAACCACAATT

Table S4 Primers of plasmid construction

Genes	Forward (5'-3')	Reverse (5'-3')

OGT	AATTAAGGCCGGCCAATGGCGTCTTCCGTGGGCAA	TTAATTGGCGCGCCTTGAAGTCACTGAGTCAGCATAA
SAP130 WT	AATTAAGGCCGGCCAATGAGTTCTCAACAGTTTCCTCGG	TTAATTGGCGCGCCGACTTTTTCCTTTCGCTTCAATTTG
SAP130 T281A	GTCTCGGCCACCTGTGACCGCCTCCAATGCCATCCCTC	GAGGGATGGCATTGGAGGCGGTCACAGGTGGCCGAGAC
SAP130 S282G	GCCACCTGTGACCACCGGCAATGCCATCCCTCCTG	CAGGAGGGATGGCATTGCCGGTGGTCACAGGTGGC
SAP130 T296A	GTAGCAACTGTCTCAGCCGCCAGAGCTCAGTCTCCAG	CTGGAGACTGAGCTCTGGCGGCTGAGACAGTTGCTAC
SAP130 S316G	CTACTGATTCAGCACTTGGTAGGCCAACCTTGTC	GACAAGGTTGGCCTACCAAGTGCTGAATCAGTAG
SAP130 T473A	CAATCCTGTGGCCATGGAAGCCCGAAGTGACAACAGACC	GGTCTGTTGTCACTTCGGGCTTCCATGGCCACAGGATTG
BTG2 promoter	CGGGGTACCAAGGTGGAAGACCAAGATTA	CCGCTCGAGTGCCCGGGGAAAGTCCGGGC

Scheme S1

1. Synthesis of compound B:

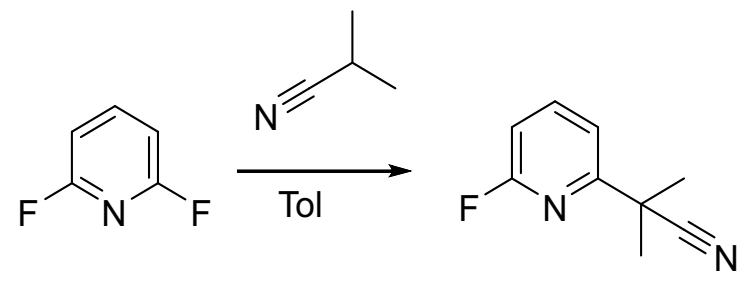

A B

To a solution of **A** (10.0 g, 144.92 mmol) and 3-methylbut-1-yne (25.0 g, 217.39 mmol) in toluene (200.0 mL) was added LiHMDS (145.0 mL), reaction was stirred at 80ºC for 12 hours. After the reaction completed, poured the reaction solution into ice water, then extracted with EA (100.0 mL*3) and the combined organic was washed with brine (50.0 mL*3) and dried over Na_2SO_4. The crude product was purified by column flash chromatography (EA/PE= 10%~ 40%) to obtain compound **B** as a while solid (18.0 g, yield:78.2 %).

LCMS: m/z = 165.2 $[M+H]^+$, Rt = 1.345 min. **Purity**: 100% (214 nm)

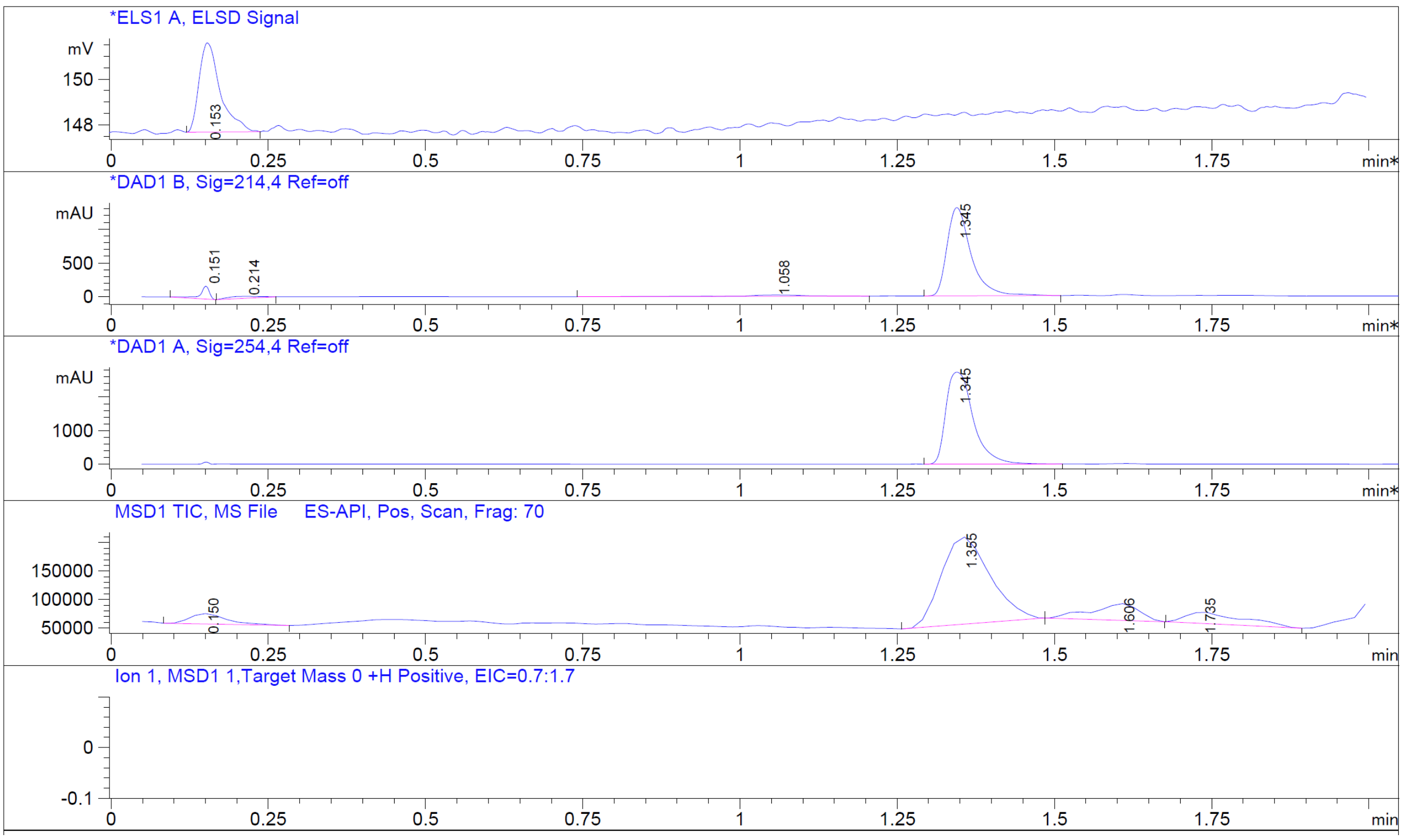

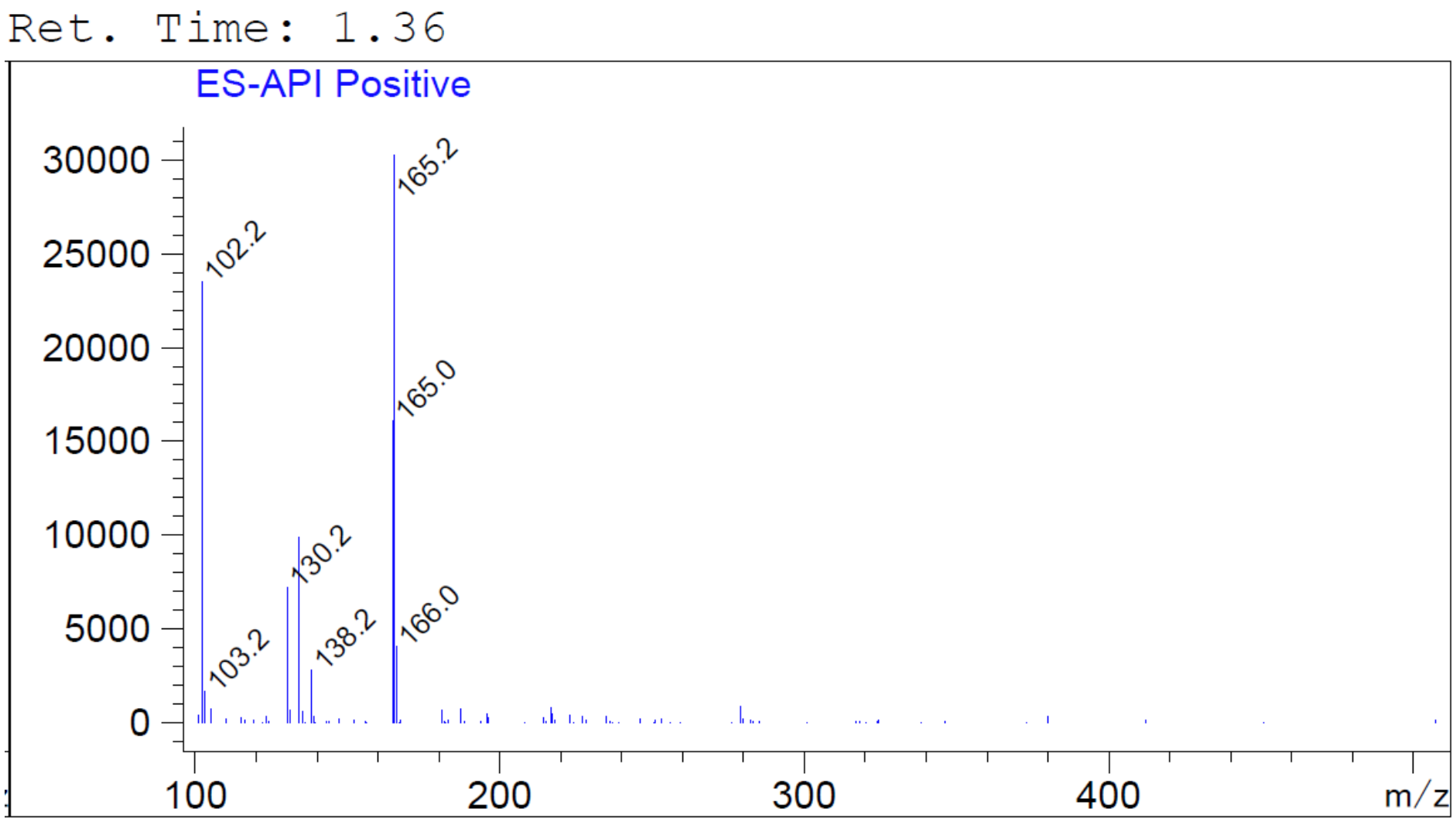

2. Synthesis of compound C:

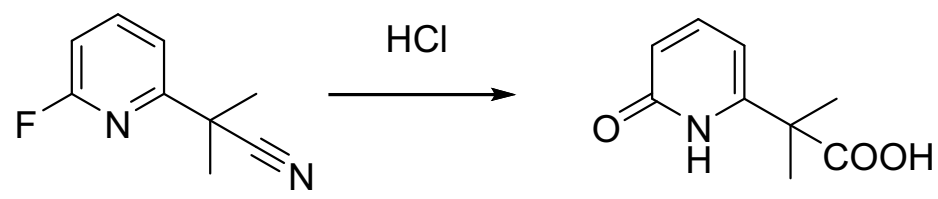

B C

The 2-(6-fluoropyridin-2-yl)-2-methylpropanenitrile (10.0 g, 6.135 mmol) was added to a round bottom flask and was added 9M HCl aq. to make 0.3M solution. The resulting mixture was heated to 90 ºC and stirred for overnight. The reaction progress can be monitored through LCMS by consumption of the starting materials. After completion, the solution was concentrated under reduced pressure then diluted with H2O. NaOH (4M) was added until the pH reaches 2~3. The resulting aqueous solution was extracted with CHCl3/IPA (3:1, 100 mL*3) and combined organic layers were dried and concentrated to afford the crude product. The crude product was dissolved in CHCl3 and washed with water. The organic layer was dried over sodium sulfate, filtered and evaporated to give 6.1 g of the title compound **C** as a white solid.

^{1}H NMR (400 MHz, Chloroform-d) δ 7.54 (dd, J = 9.2, 7.2 Hz, 1H), 6.56 (dd, J = 9.2, 1.2 Hz, 1H), 6.41 (dd, J = 7.2, 0.8 Hz, 1H), 1.64 (s, 6H).

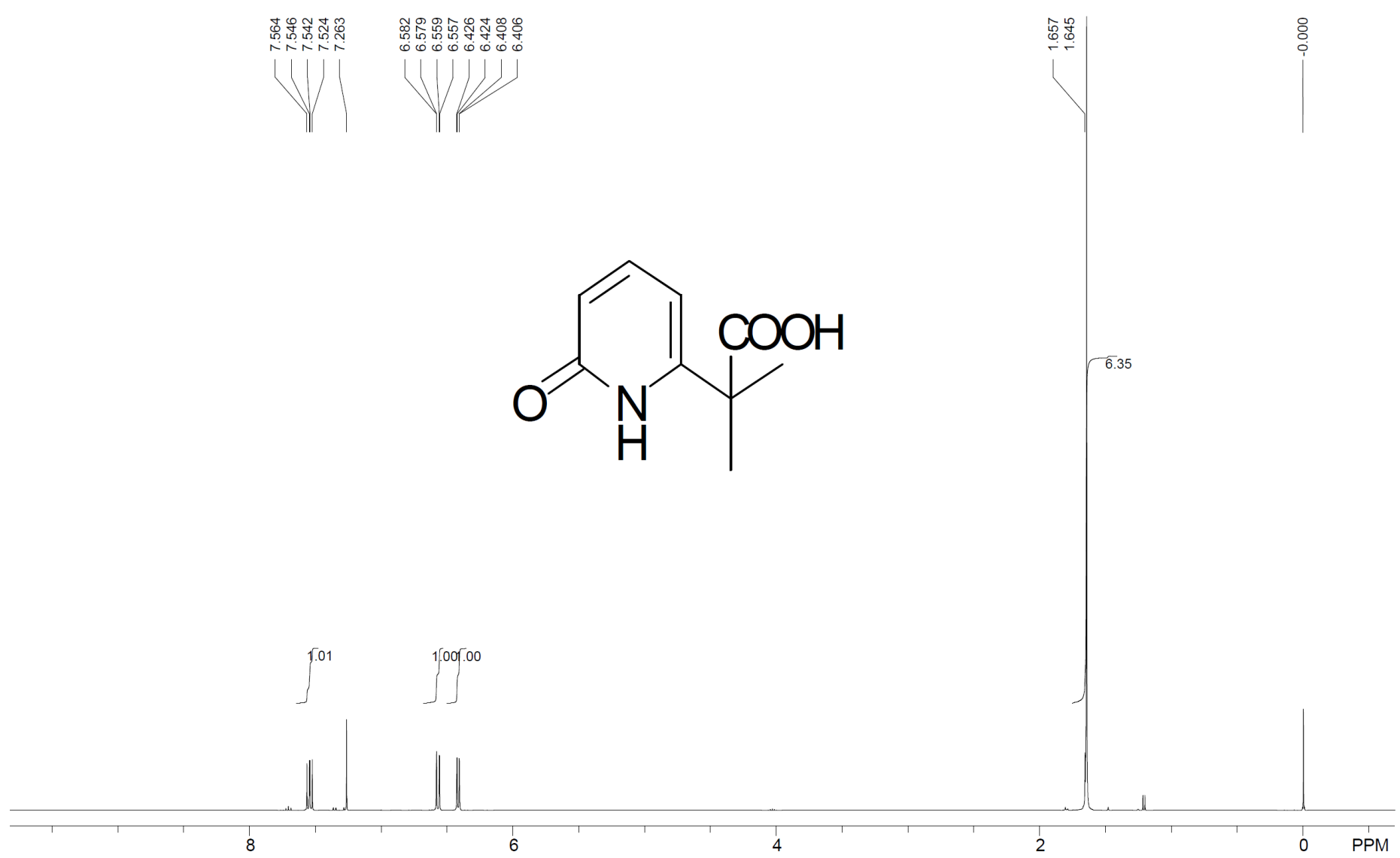

3. **Synthesis of compound 2**:

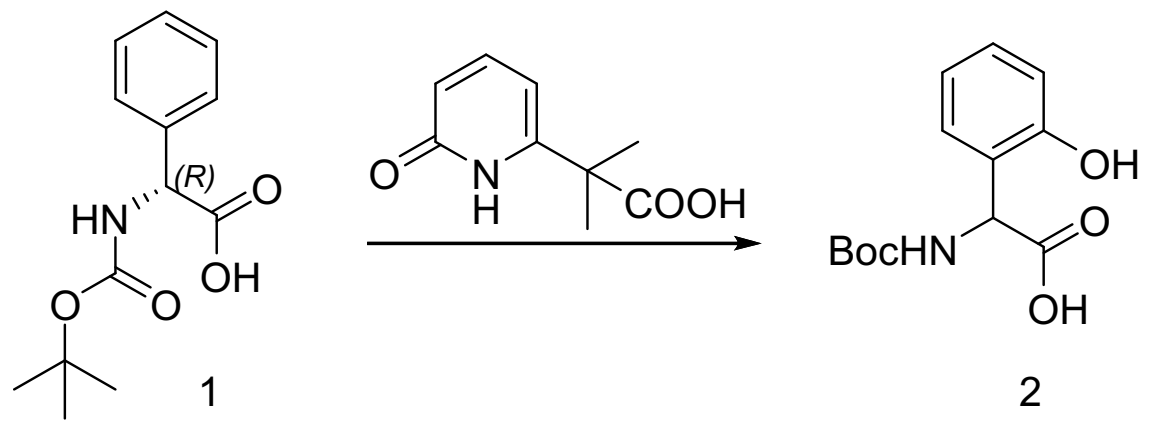

To a solution of compound **1** (40.0 g, 159.36 mmol) in ACN (500.0 mL) was added 2-methyl-2-(6-oxo-1,6-dihydropyridin-2-yl)propanoic acid (1.2 g, 6.63 mmol) , $Pd(OAc)_2$(720 mg, 3.21 mmol), K_2HPO_4(42.0 g, 241.37 mmol), the reaction was stirred at RT for 10 min, then added H_2O_2(40.0 mL,30% in water). The reaction mixture was stirred at room temperature for 18 hours under N_2. Upon completion, the reaction was diluted with methanol and acidified with 40 mL formic acid. The solution was filtered through a pad of Celite and washed with methanol then concentrated under vacuum). The crude mixture was purified by flash chromatography (DCM/MeOH with 1% HCOOH, v/v) to obtain compound **2** as a light yellow solid (32.7 g, yield:73%).

LCMS: m/z = 290.0 $[M+Na]^+$, Rt = 1.310 min. **Purity**: 98.58% (254 nm).

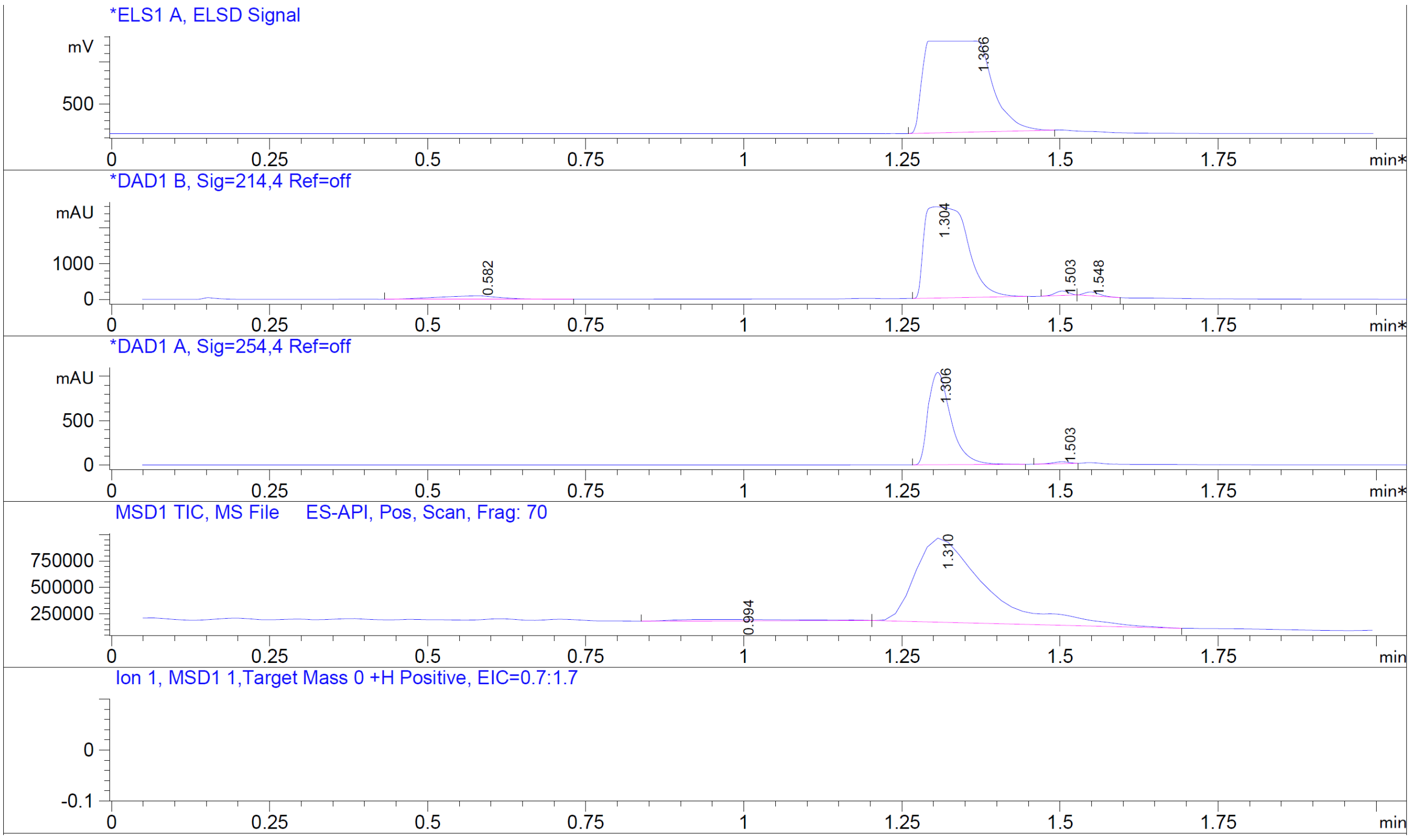

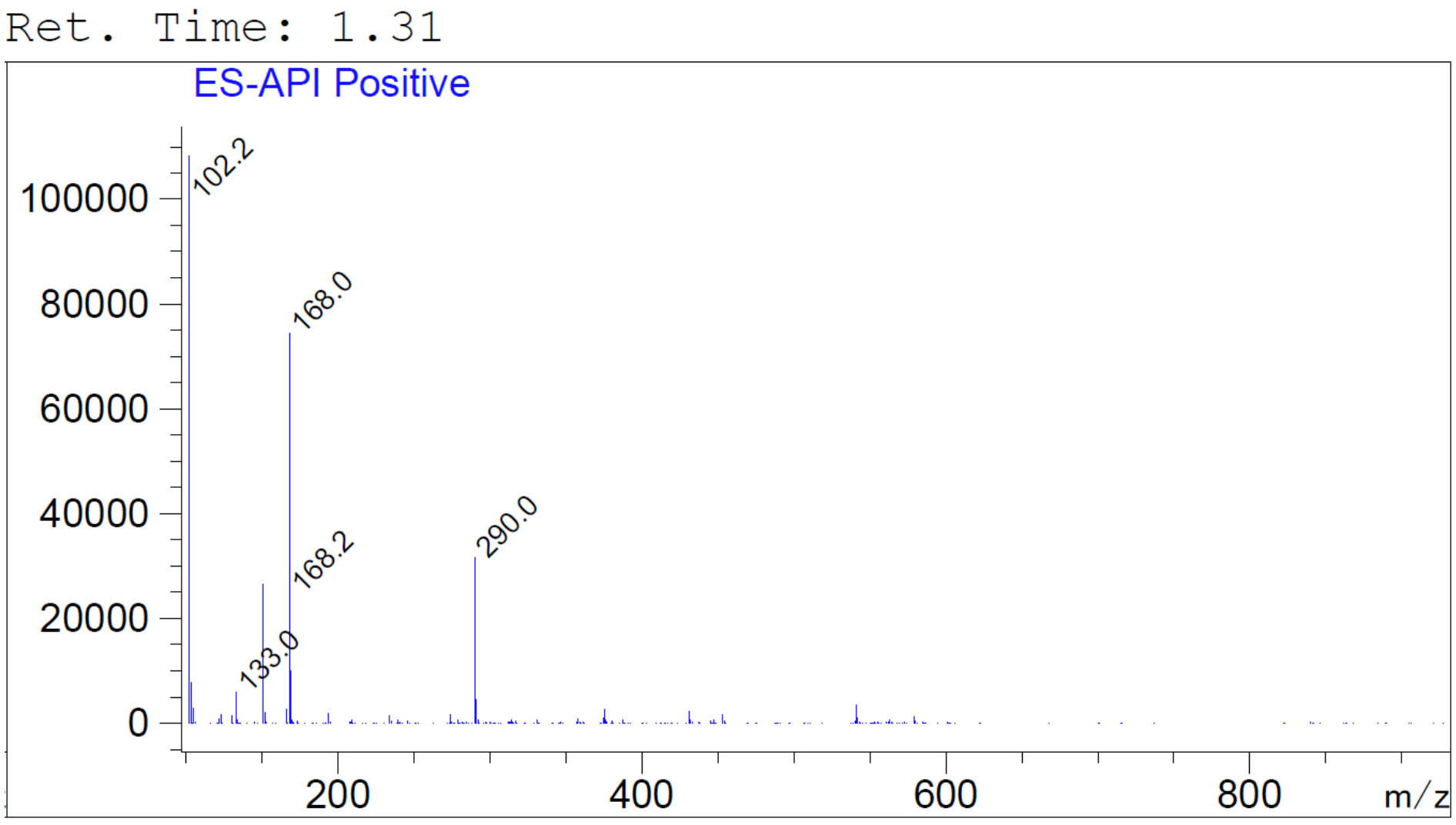

4. Synthesis of compound 3:

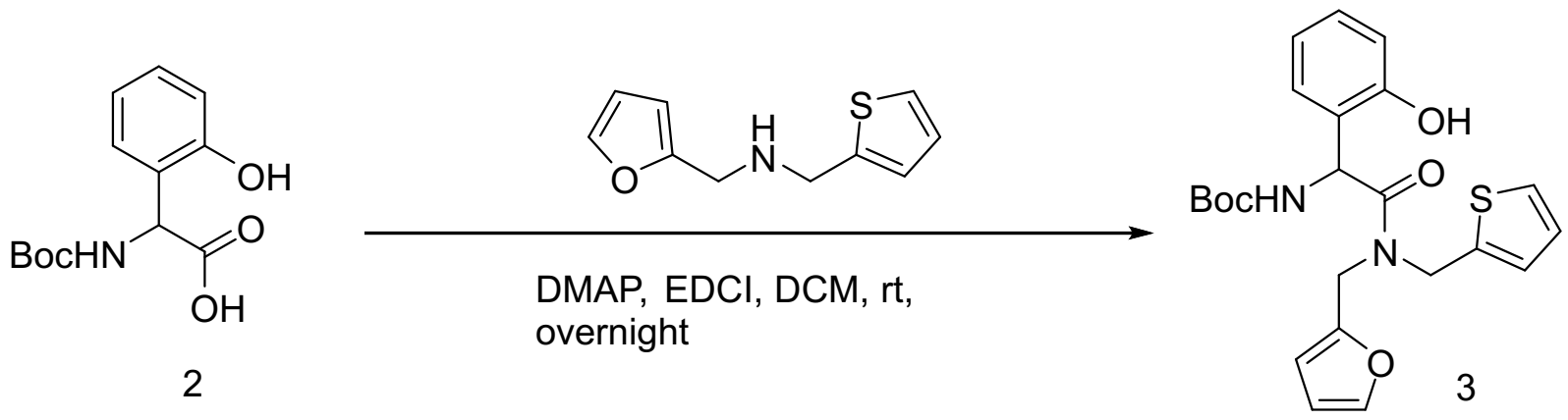

To a solution of compound **2** (20.0 g, 74.91 mol) in DCM (100.0 mL) was added 1-(furan-2-yl)-N-(thiophen-2-ylmethyl)methanamine (17.4 g， 90.15 mol), EDCI(44.0 g, 229.16 mmol), DMAP (26.0 g, 213.11 mmol), reaction was stirred at room temperature for 2 hours under N_2. After the reaction was completed, the reaction solution was concentrated, and the residue was purified by silica gel column chromatography(PE/EA=8/1) to obtain compound **3** as a light yellow solid (9.7 g, yield: 32%).

LCMS: m/z = 465.2 $[M+Na]^+$, Rt = 2.18 min. **Purity**: 81.6% (214 nm).

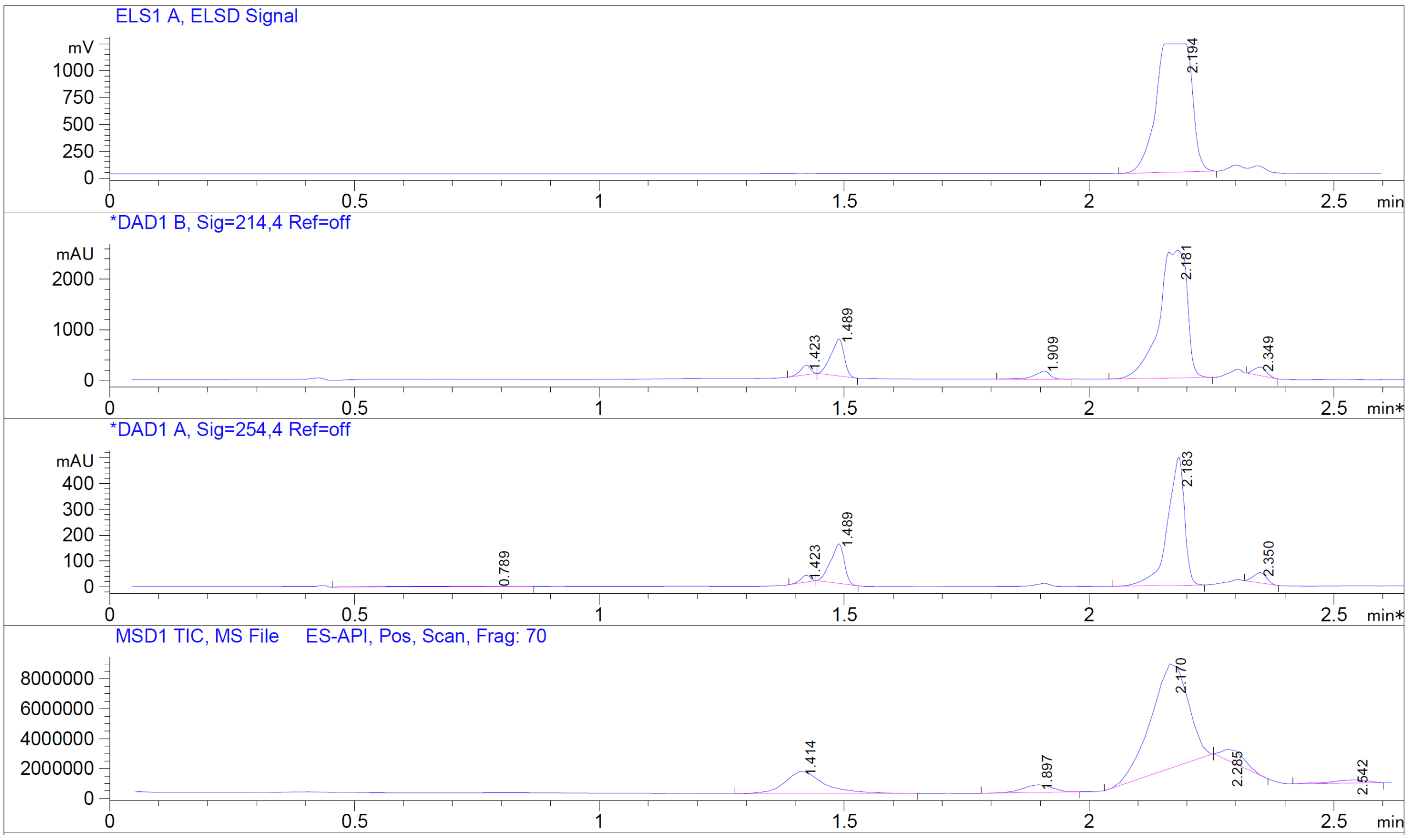

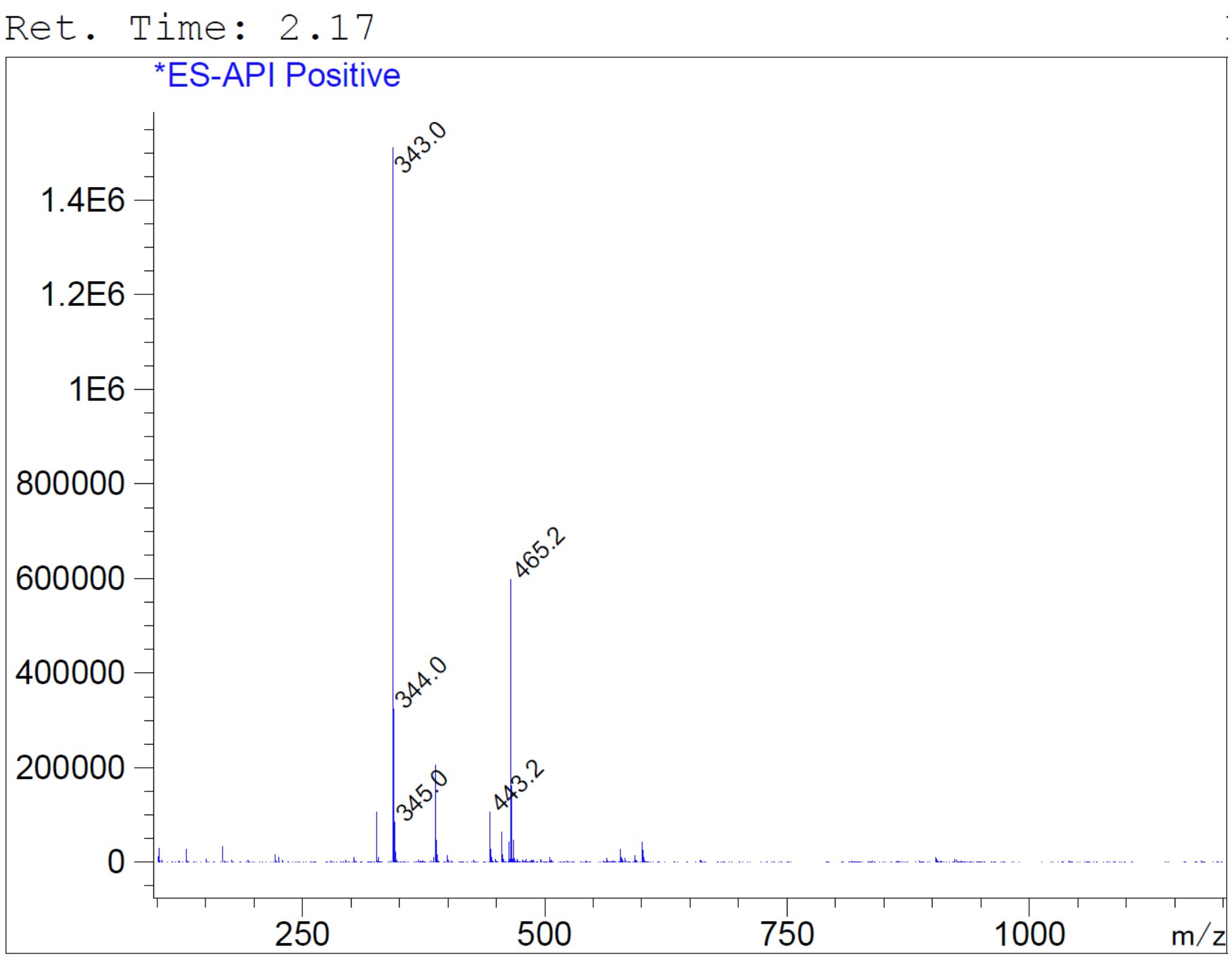

5. Synthesis of compound 4:

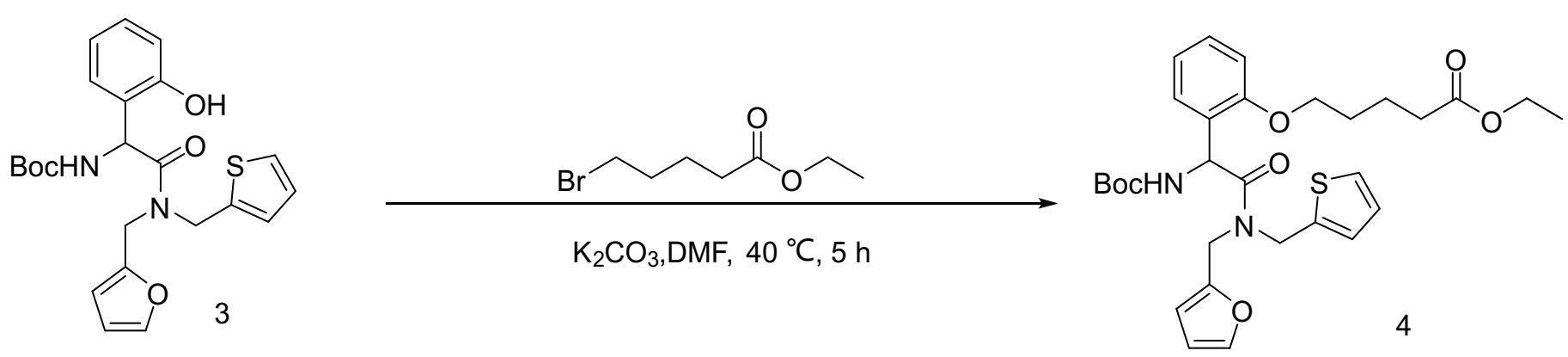

To a solution of compound **3** (9.7 g, 21.84 mmol) in DMF (500.0 mL) was added ethyl 5-bromopentanoate (5.5 g, 26.31 mmol), K_2CO_3(9.1 g, 65.94 mmol), reaction was stirred at 50°C for 2 hours. After the reaction was completed, the mixture was poured into water (100.0 mL) and extracted with EA (100.0 mL*3) and the combined organic was washed with brine (100.0 mL*3) and dried over Na_2SO_4. The crude product was purified by column flash chromatography(PE/EA=8/1) to obtain compound **4** as a light yellow solid (10 g, yield: 83 %). **LCMS**: m/z = 571.2 $[M+H]^+$, Rt = 1.861 min. **Purity**: 99.0% (214 nm).

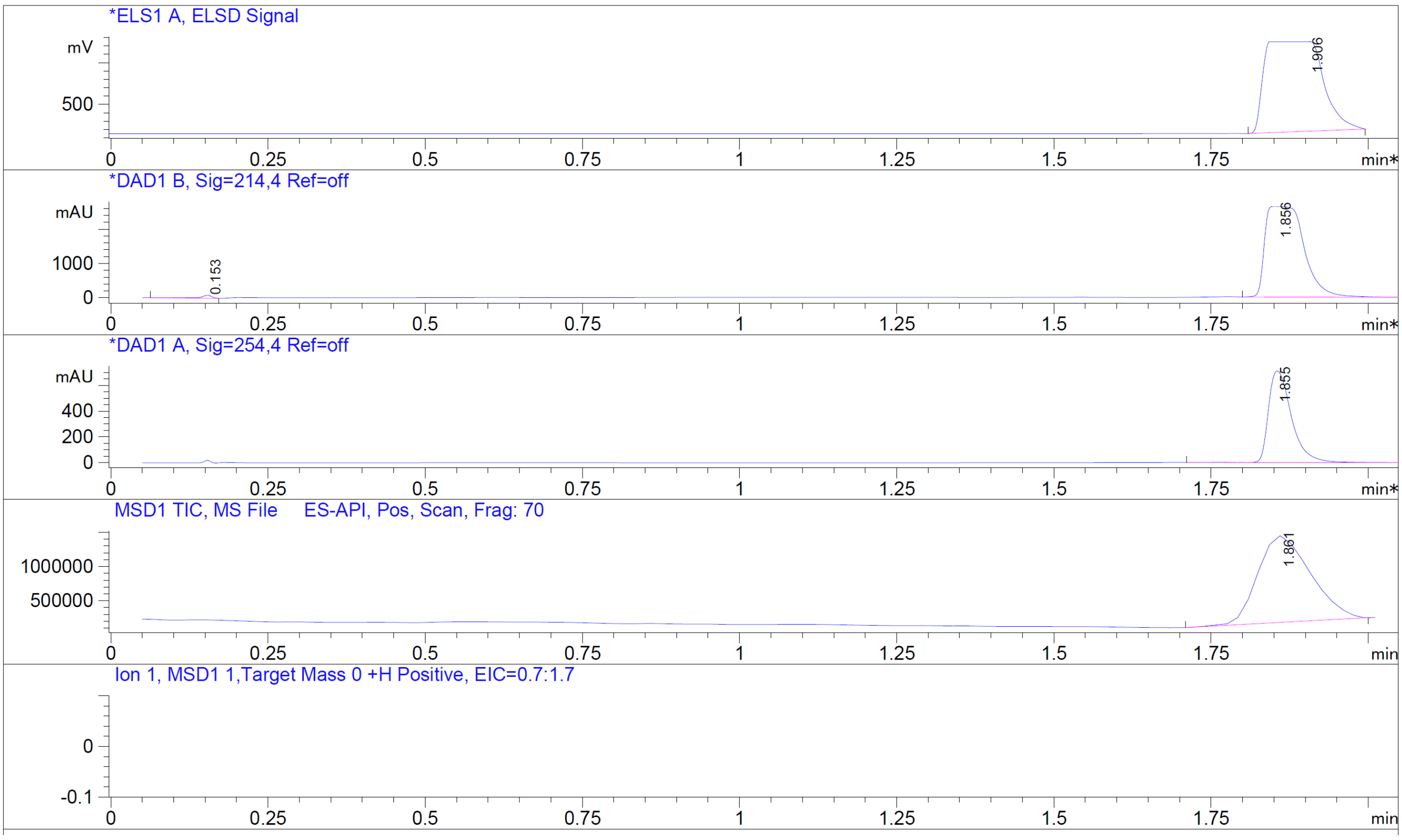

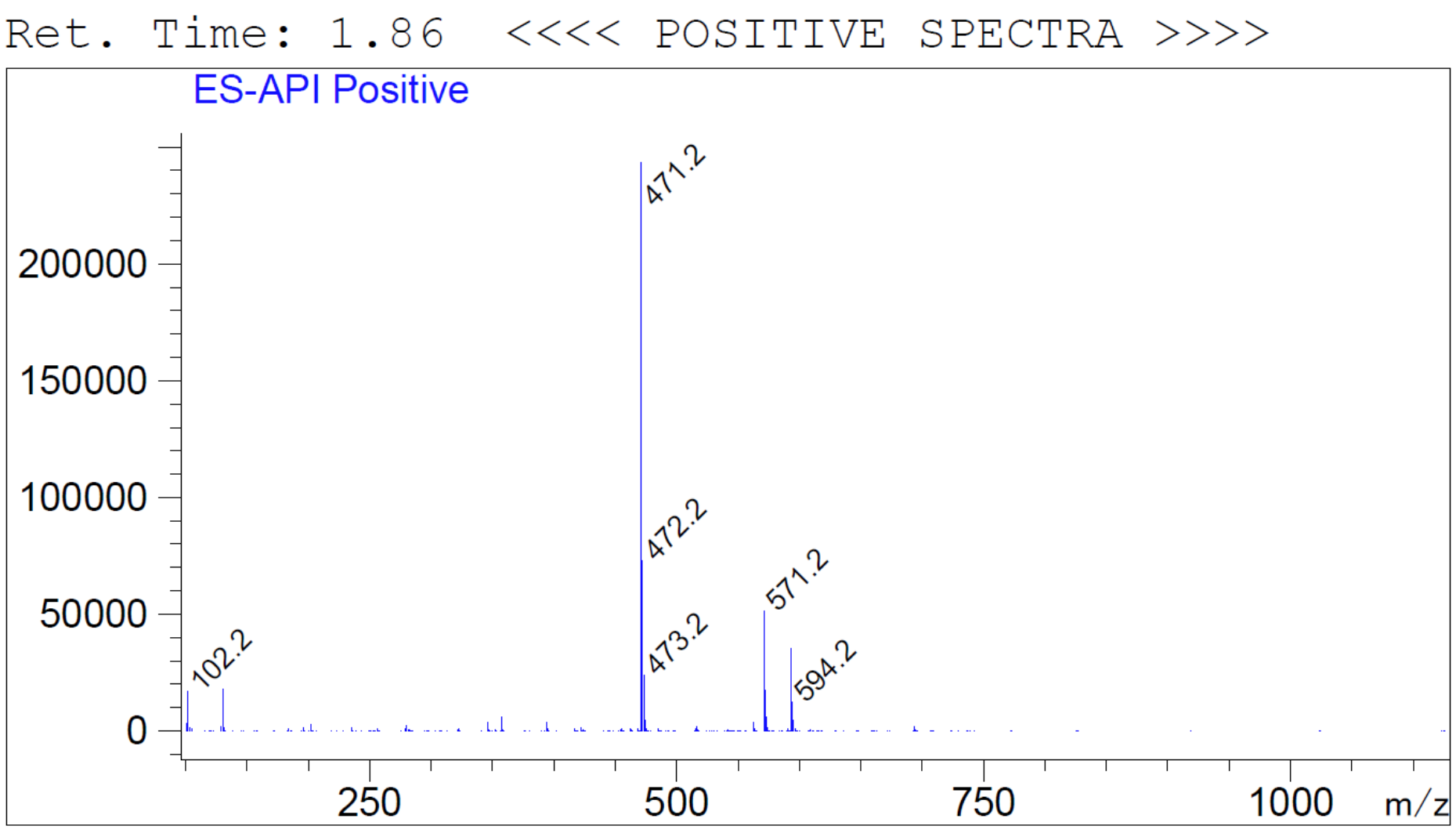

6. Synthesis of compound 5:

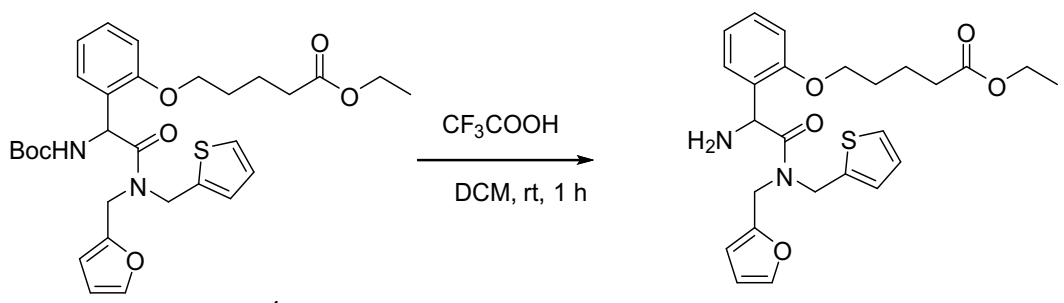

To a solution of compound **4** (10.0 g, 17.54 mmol) in DCM (100.0 mL) added TFA (20.0 mL), reaction was stirred at RT for 1 hours. After the reaction completed, the reaction was directly concentrated to obtain a product **5** (14.0 g) that can be used directly in the next step.

LCMS: m/z = 471.2 $[M+H]^+$, Rt = 1.823 min. **Purity**: 100% (254 nm).

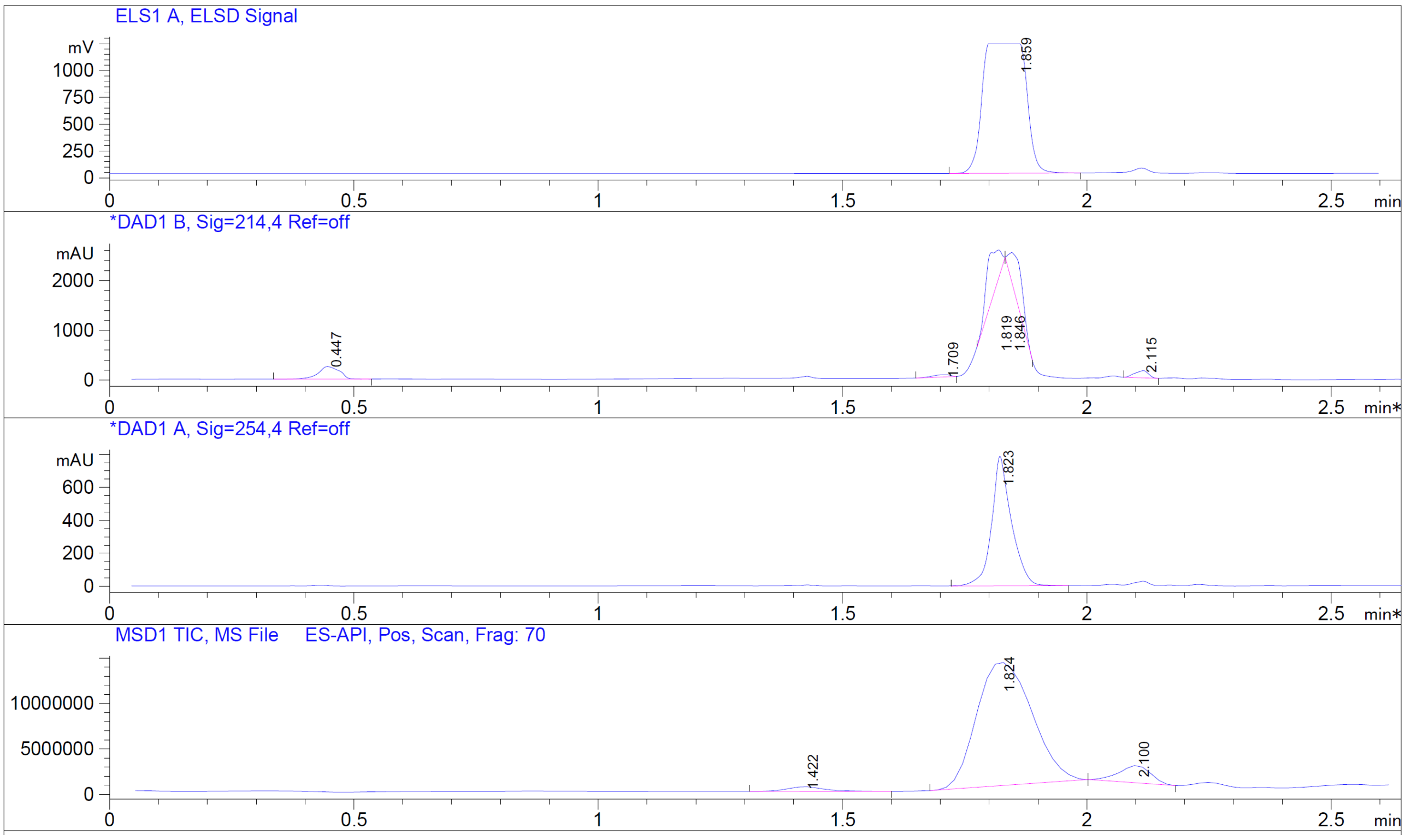

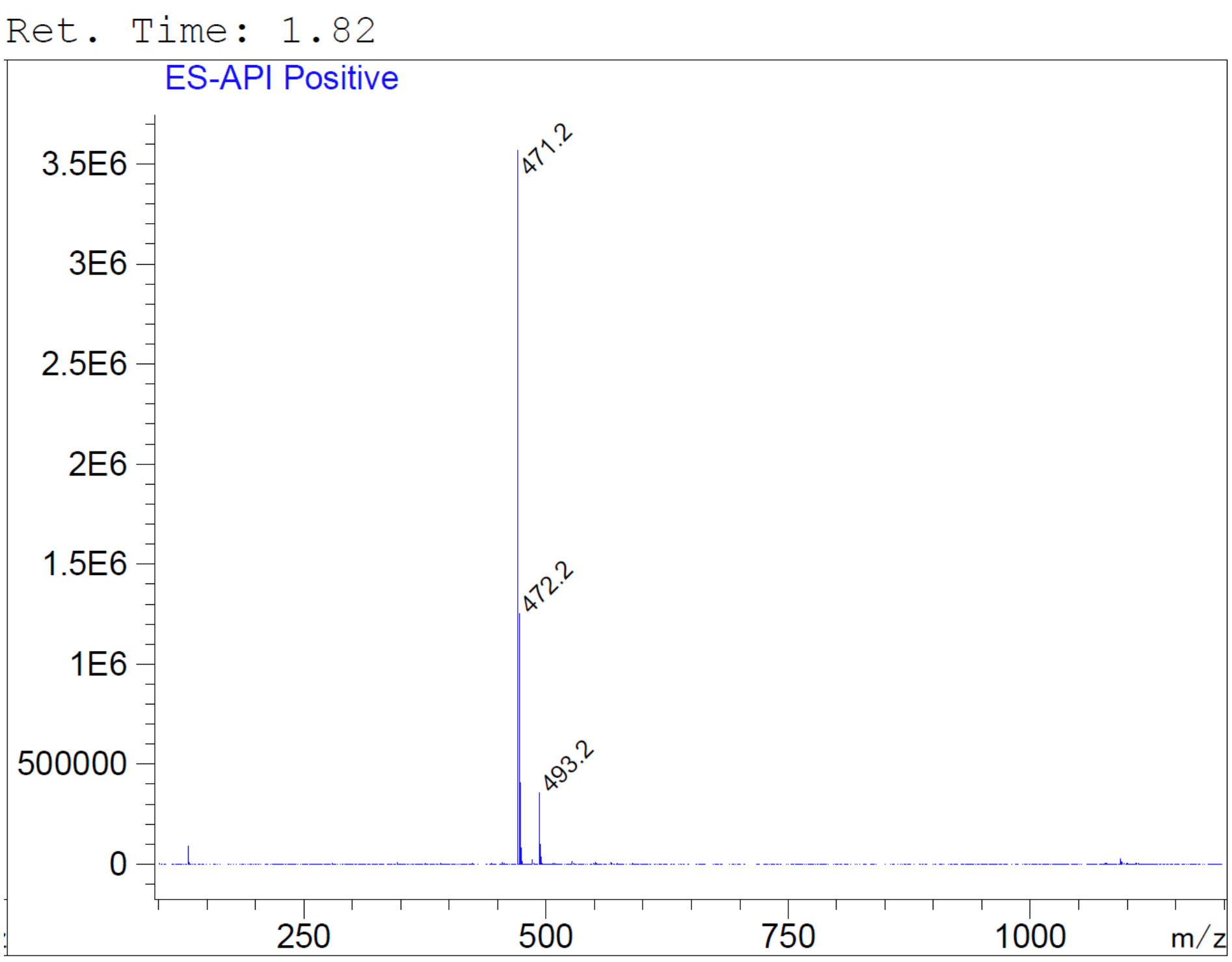

7. Synthesis of compound 6:

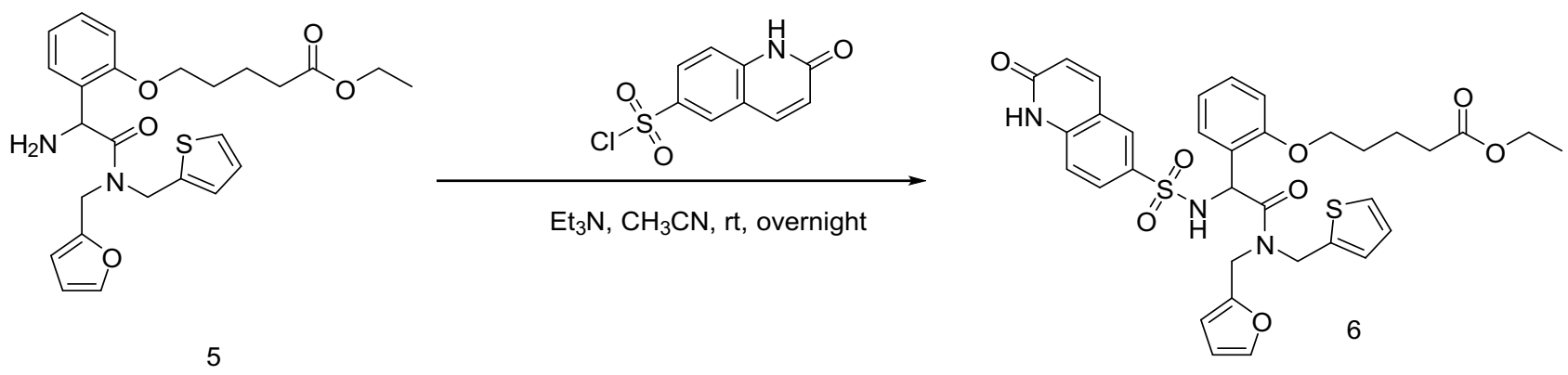

To a solution of compound **5** (14.0 g, 17.02 mmol) in ACN (150.0 mL) was added 2-oxo-1,2-dihydroquinoline-6-sulfonyl chloride (5.5 g, 22.63 mmol), TEA (21.0 mL), reaction was stirred at RT for 12 hours. After the reaction completed, the reaction solution was filtered, and the filter cake was washed with acetonitrile (50.0 mL) and dried to obtain compound **6** as a light yellow solid (7.0 g, yield:63 %).

LCMS: m/z = 678.2 $[M+H]^+$, Rt = 1.442 min. **Purity**: 98.2% (214 nm).

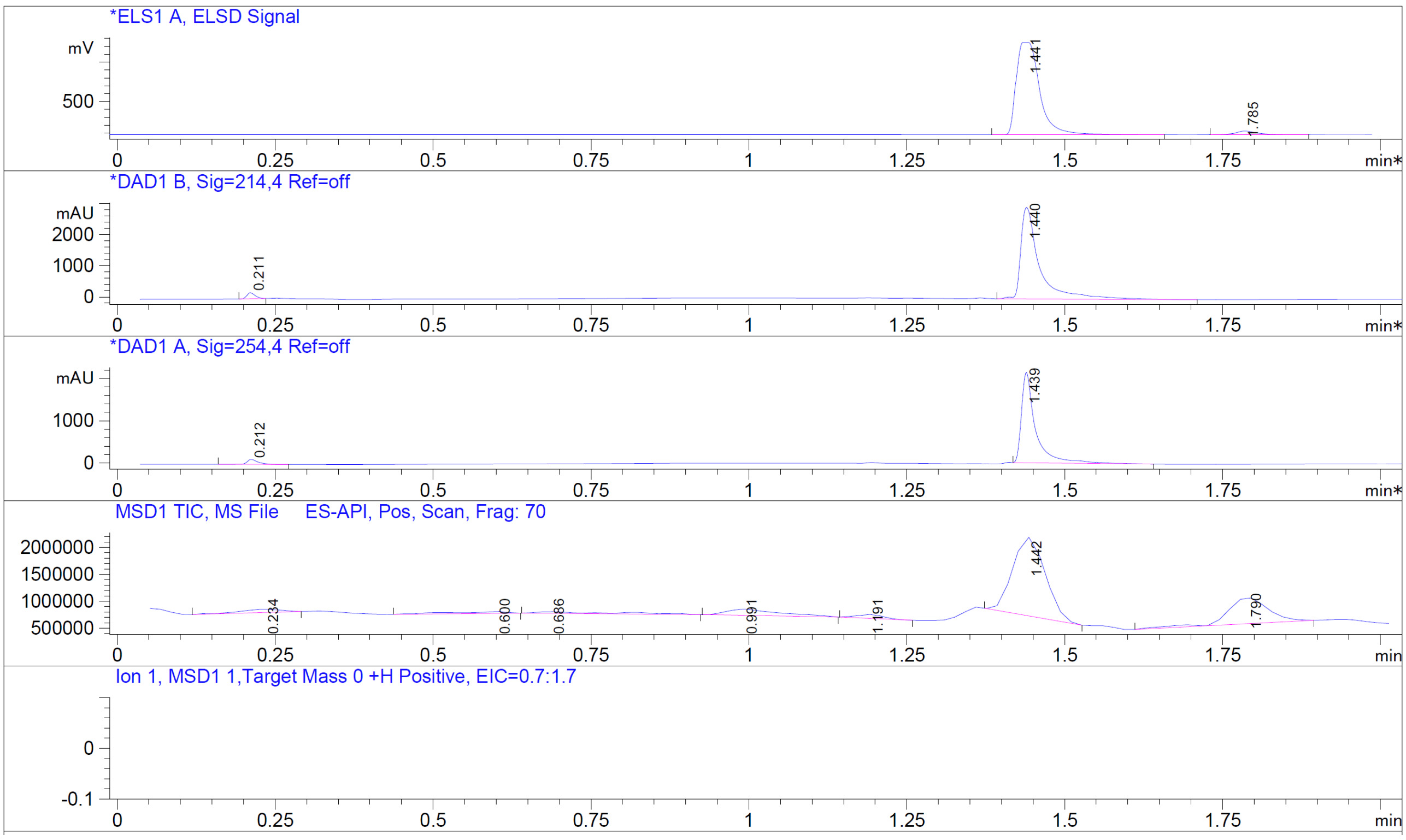

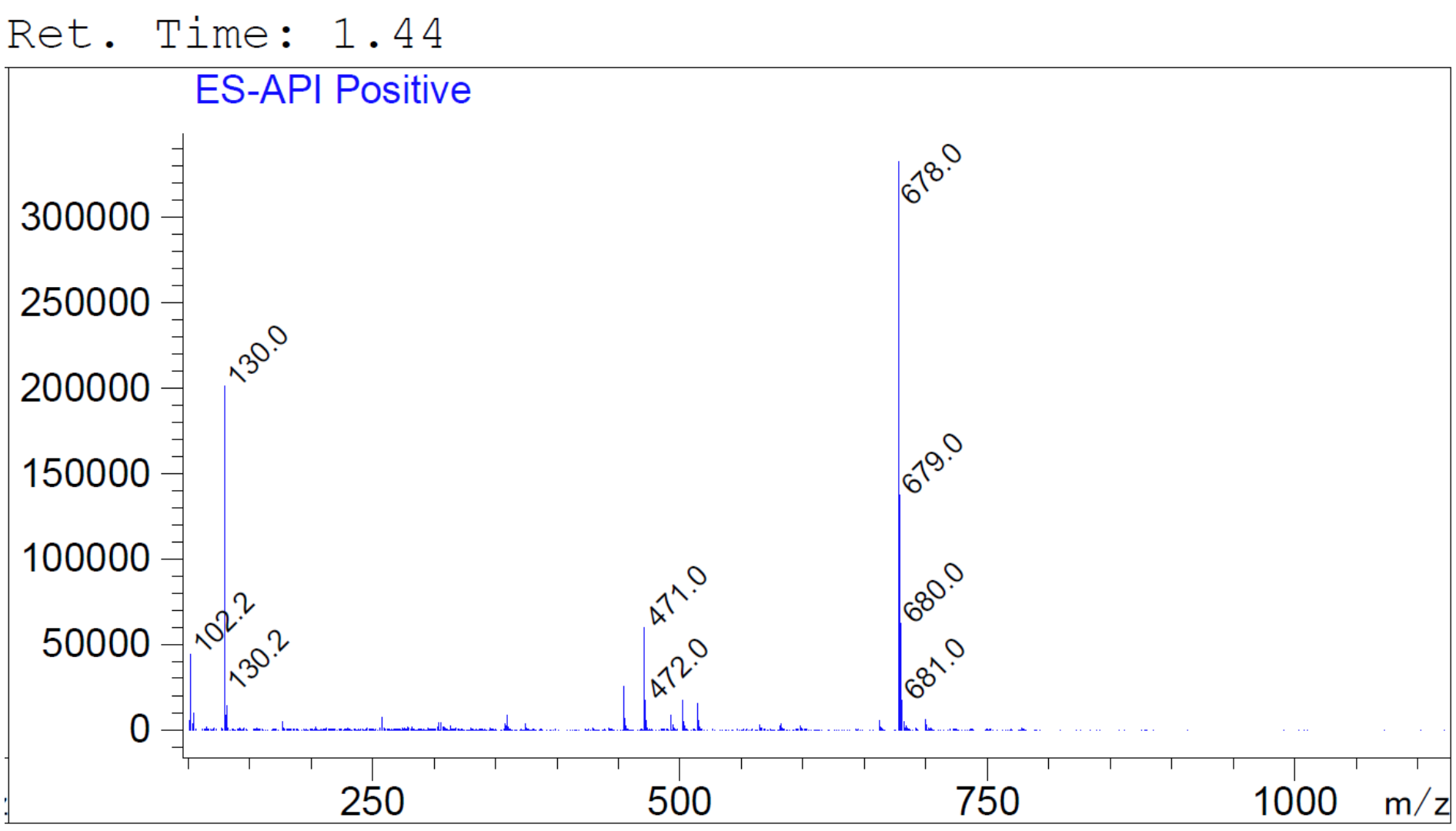

8. Synthesis of compound 7:

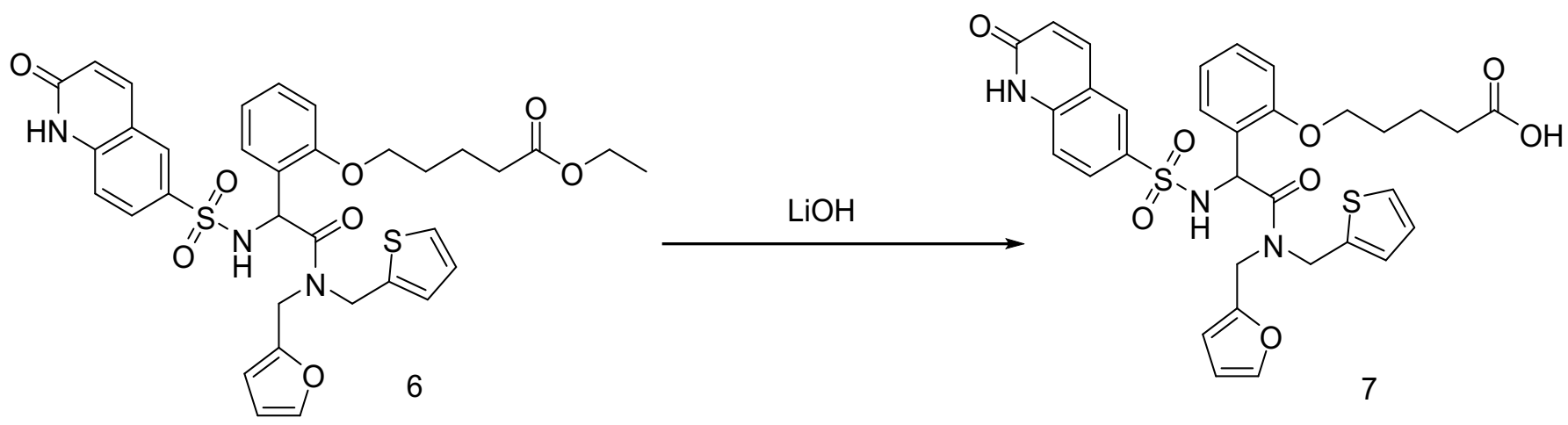

To a solution of compound **6** (7.0 g, 10.34 mmol) in THF/MeOH=4/1 (100.0 mL) was added LiOH (20.0 mL, 1M), the reaction was stirred at room temperature for 1 hour. After the reaction was completed, added diluted hydrochloric acid dropwise to the reaction solution to adjust the pH to weakly acidic. the mixture was poured into water (50.0 mL) and extracted with EA (50.0 mL*3) and the combined organic was washed with brine (50.0 mL*3) and dried over Na_2SO_4. The crude product was purified by column flash chromatography to obtain compound **7** as a white solid (5.7 g, yield:85.1 %).

LCMS: m/z = 650.2 $[M+H]^+$, Rt = 1.913 min. **Purity**: 86.5% (214 nm).

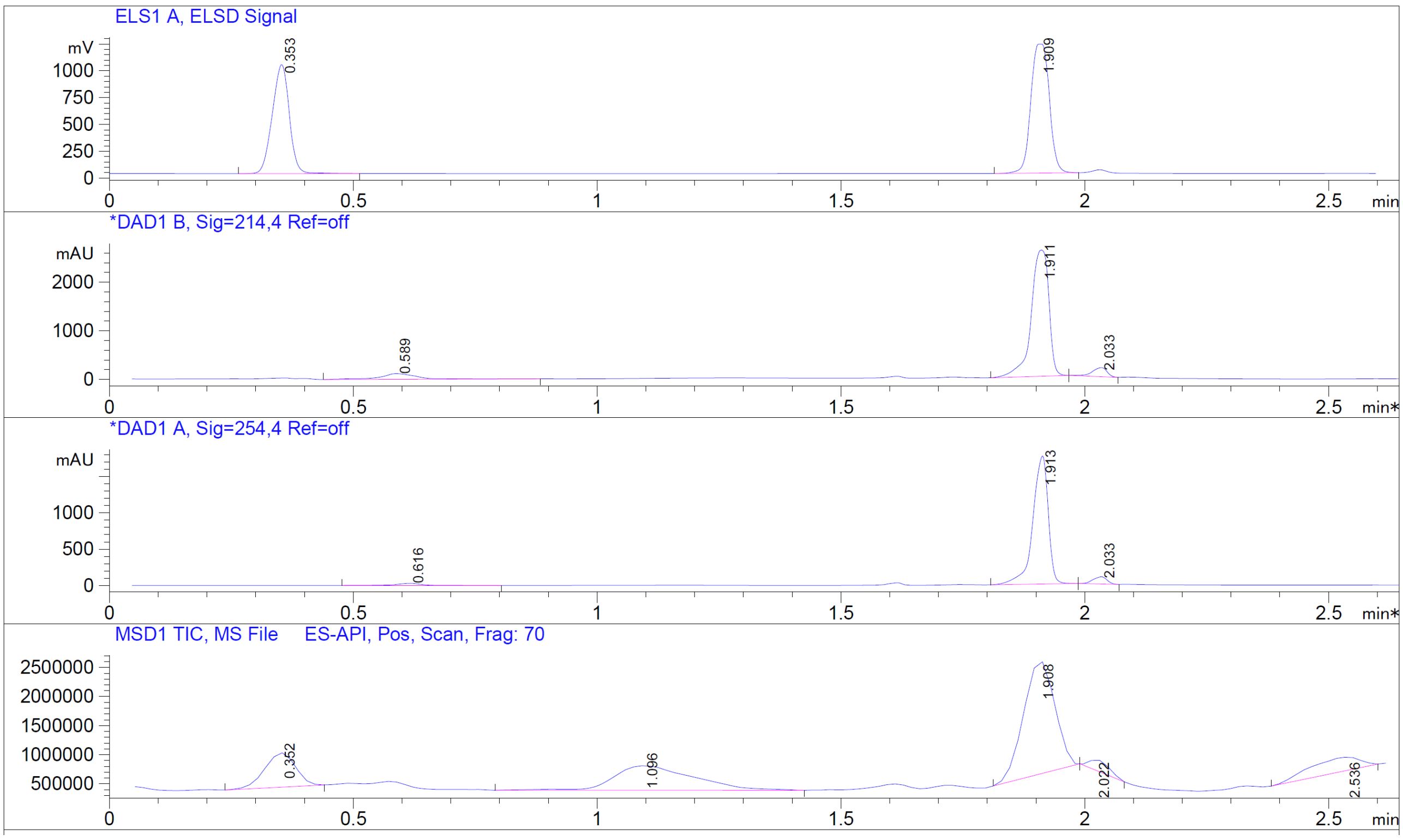

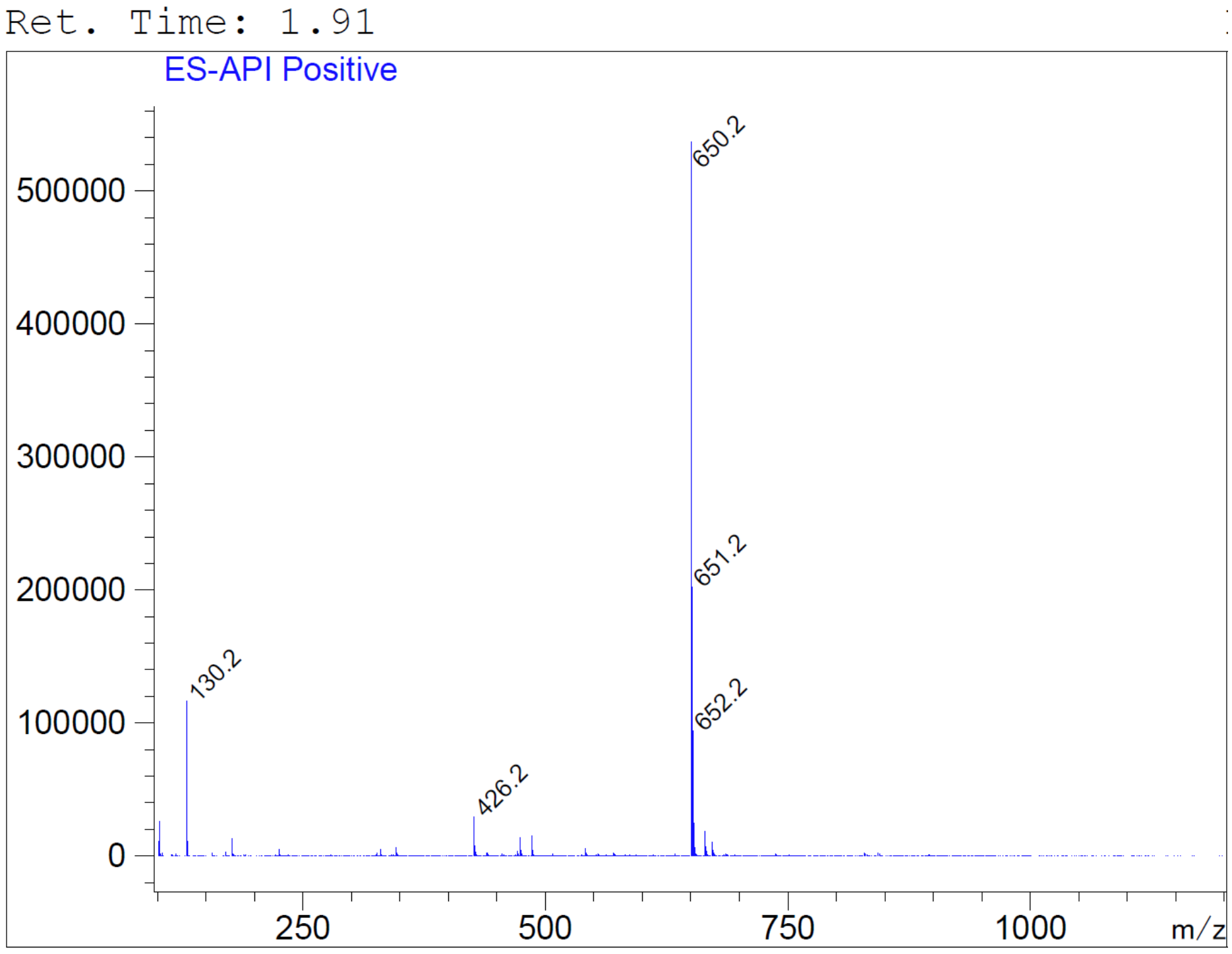

9. Synthesis of compound 8:

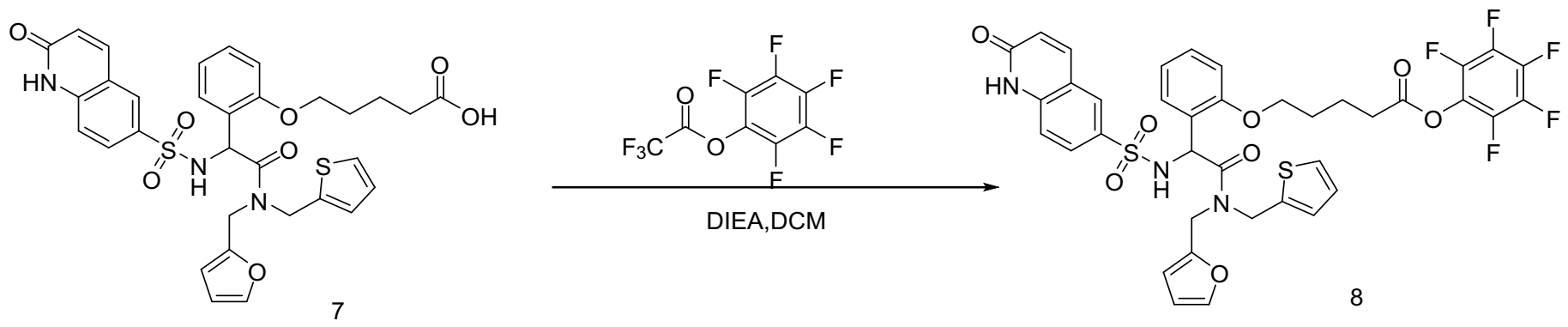

To a solution of compound **7** (4.2 g, 6.47 mmol) in DCM (50.0 mL) was added perfluorophenyl 2,2,2-trifluoroacetate (2.7 mL), DIEA (5.6 mL), the reaction was stirred at room temperature for 12 hours. After the reaction was completed, the mixture was poured into water (50.0 mL) and extracted with DCM (50.0 mL*3) and the combined organic was washed with brine (50.0 mL*3) and dried over Na_2SO_4. The crude product was purified by column flash chromatography (DCM/MeOH=10/1) to obtain compound **8** as a brown solid (5.0 g, yield:96.1 %).

LCMS: m/z = 816.0 $[M+H]^+$, Rt = 1.802 min. **Purity**: 89.6% (214 nm).

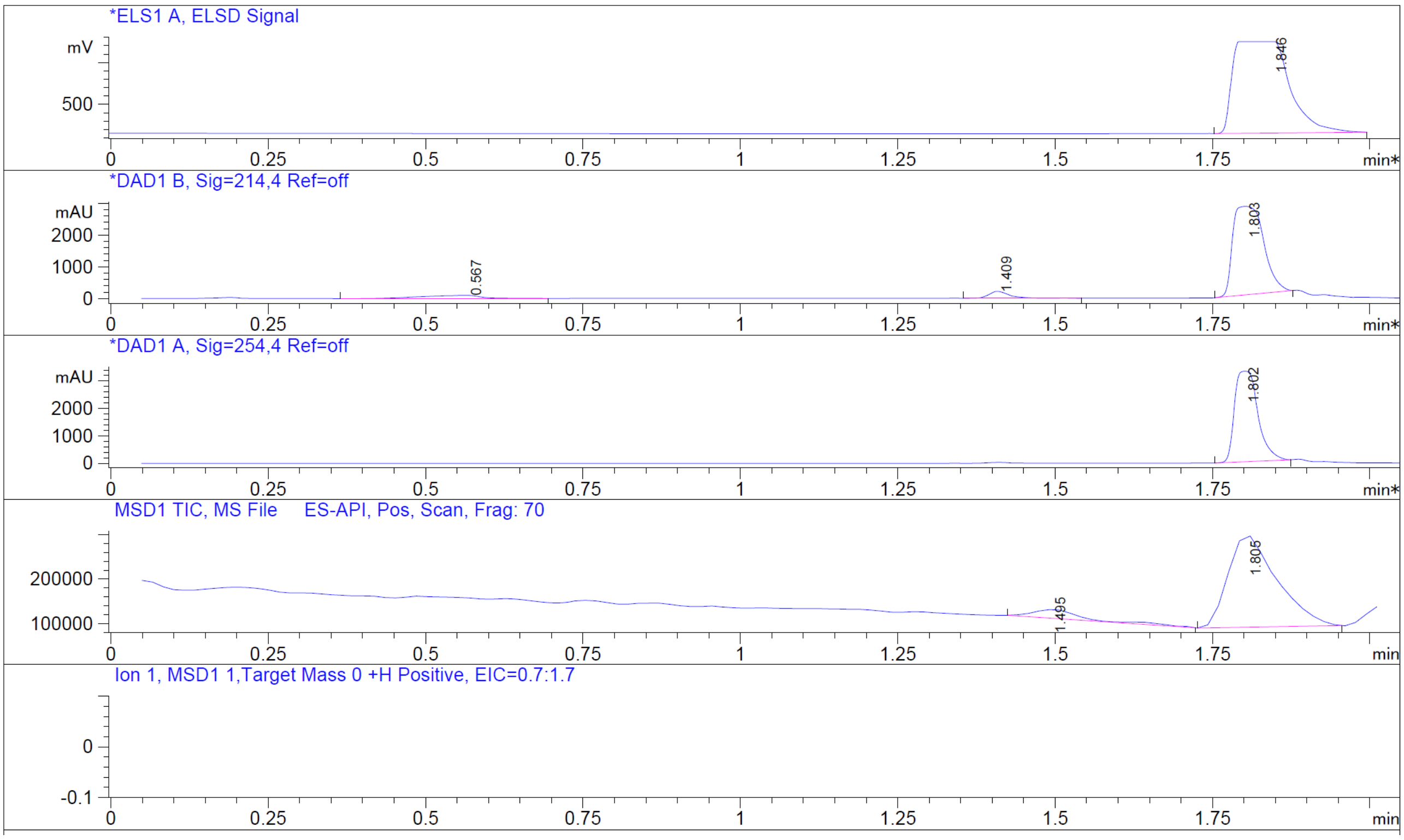

Ret. Time: 1.81

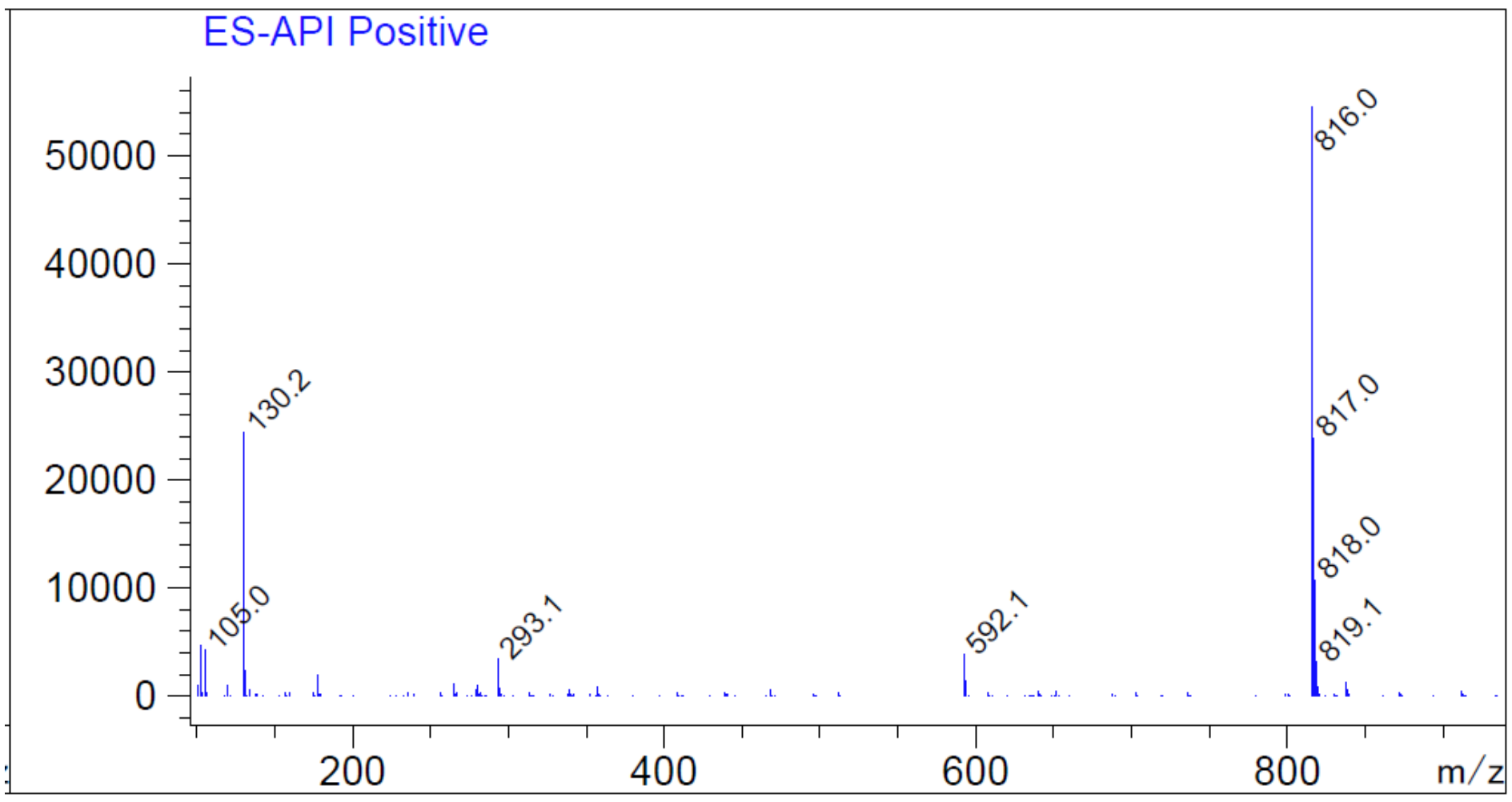

10. Synthesis of compound **9**:

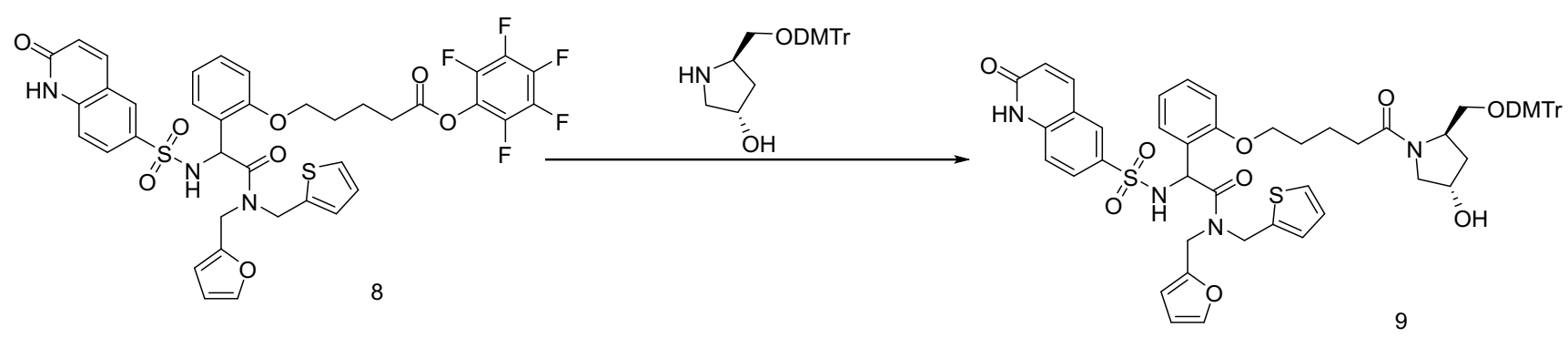

To a solution of compound **8** (4.7 g, 5.76 mmol) in ACN (50.0 mL) added (3S,5R)-5-((bis(4-methoxyphenyl)(phenyl)methoxy)methyl) pyrrolidin-3-ol (3.6 g, 8.59 mmol), DIEA(3.0 mL), reaction was stirred at room temperature for 2 hours. After the reaction was completed, the mixture was poured into water (50.0 mL) and extracted with EA (50.0 mL*3) and the combined organic was washed with brine (50.0 mL*3) and dried over Na_2SO_4. The crude product was purified by column flash chromatography (DCM/MeOH=10/1) to obtain compound **9** as a light yellow solid (6.0 g, yield: 99.1 %).

LCMS: m/z = 1049.2 [M-H]$^-$, Rt = 1.821 min. **Purity**: 96.4% (214 nm).

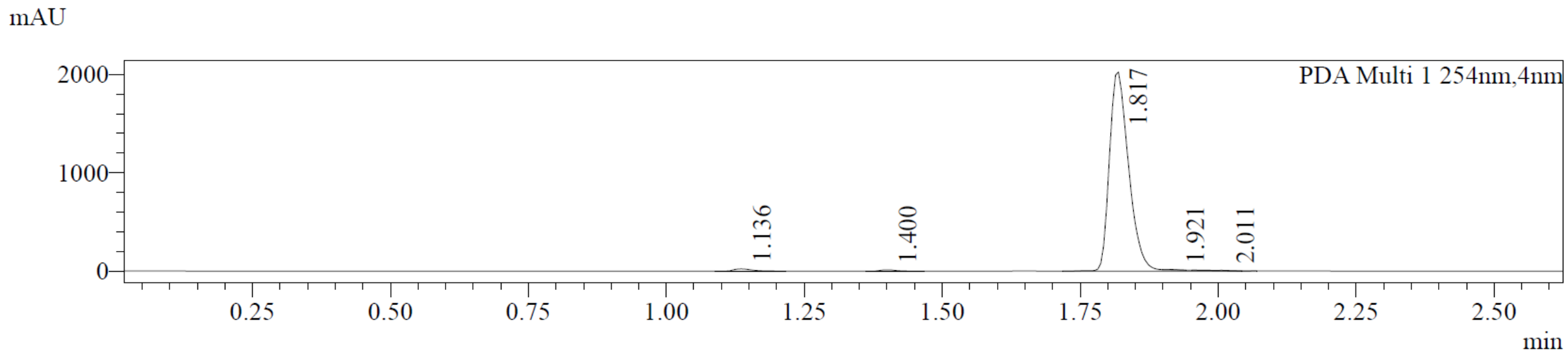

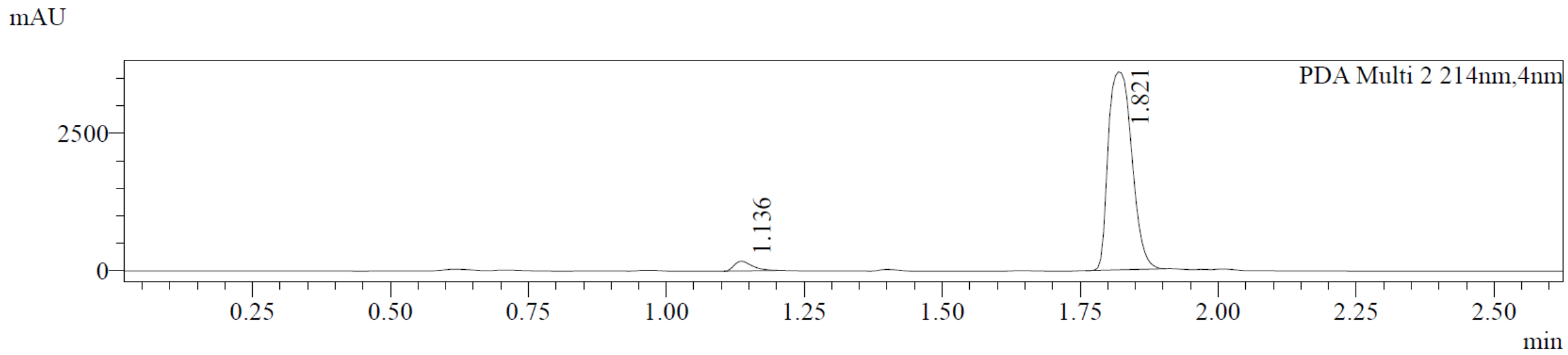

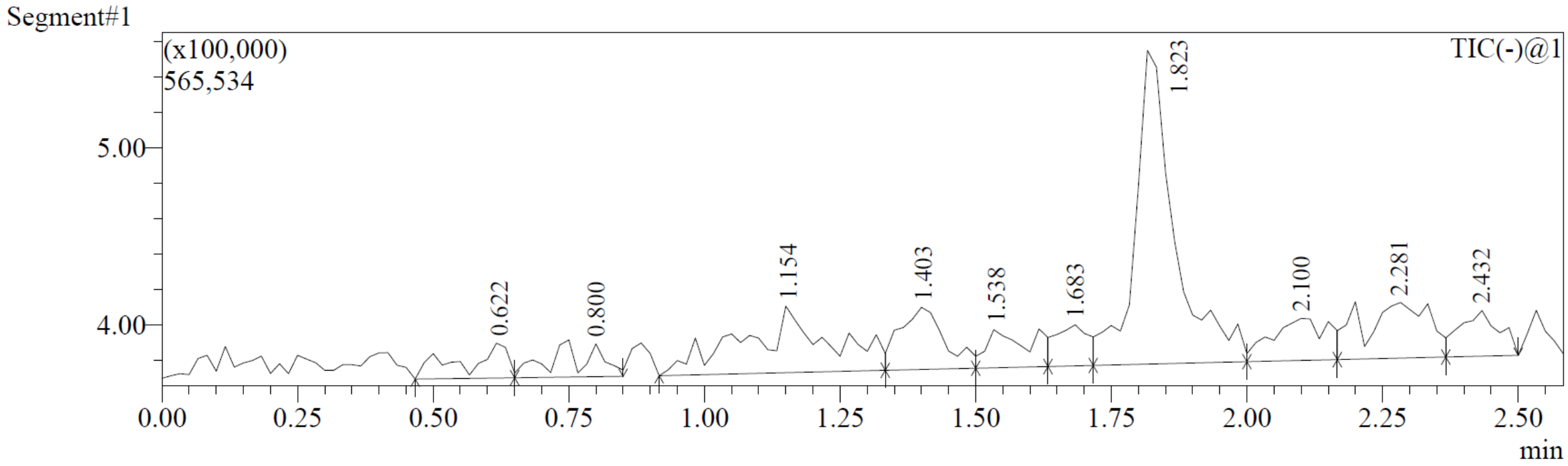

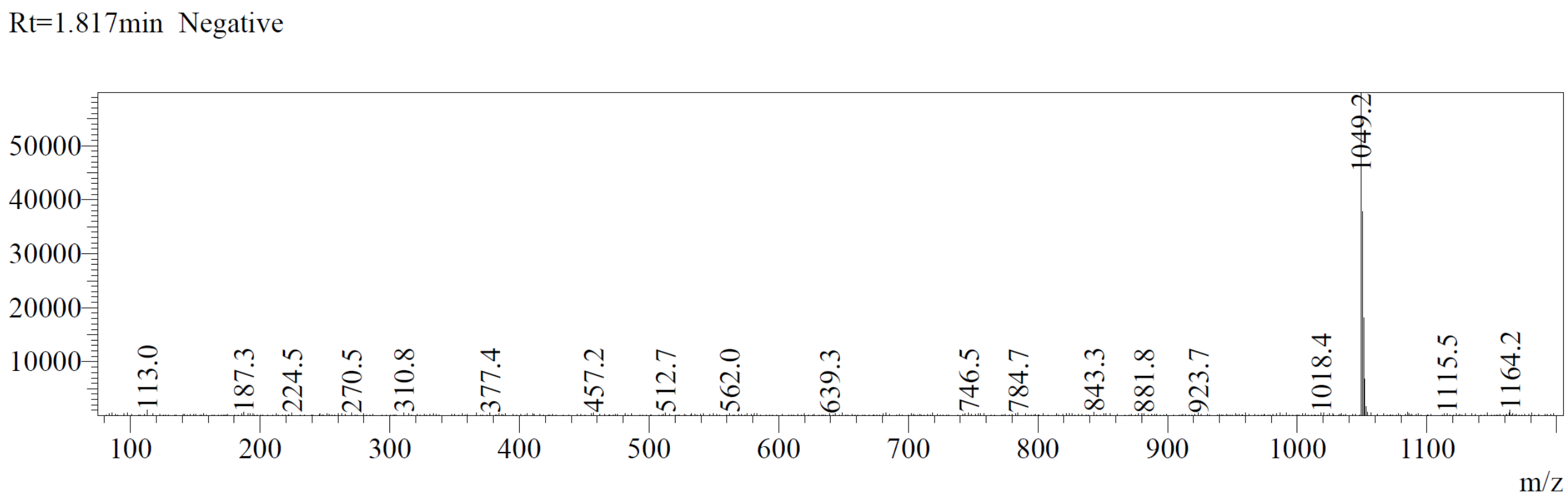

11. Synthesis of compound 10:

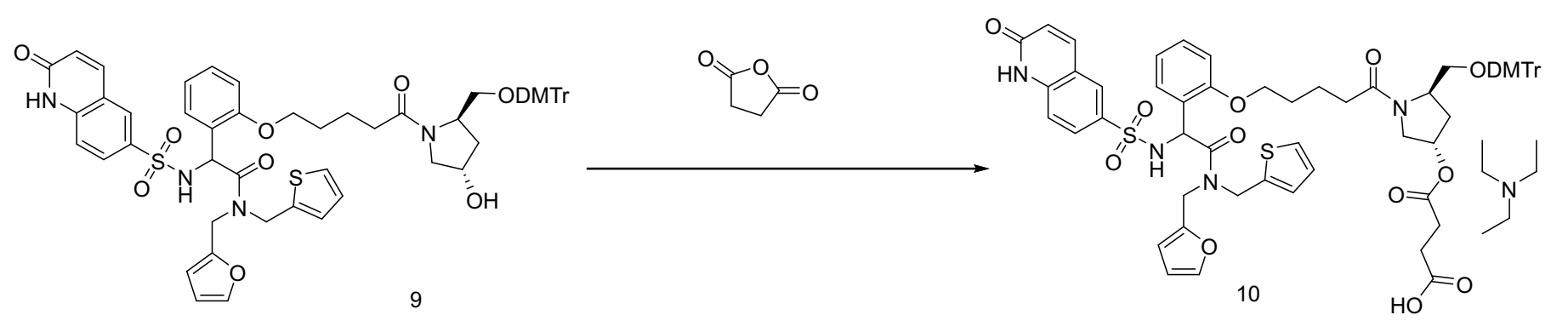

To a solution of compound **9** (6.0 g, 5.71 mmol) in DCM (50.0 mL) added succinic anhydride (1.2 g, 12.0 mmol), DMAP (1.38 g, 11.31 mmol), the reaction was stirred at room temperature for 0.5 hours. After the reaction

completed, the reaction solution was concentrated, and the residue was purified using reverse-phase column chromatography (ACN(0.1% TEA) /H_2O=1/1) to obtain compound **10** as a white solid(2.2 g, yield:31.9 %).

LCMS: m/z = 1149.6 [M-H]$^-$, Rt = 7.509 min. **Purity**: 100% (214 nm)

^{1}H NMR (400 MHz, DMSO-d_6) δ 12.16 (s, 1H), 8.40 (s, 1H), 7.90 – 7.76 (m, 2H), 7.68 – 7.47 (m, 2H), 7.37 – 7.09 (m, 13H), 6.92 – 6.80 (m, 8H), 6.59 – 6.44 (m, 1H), 6.41 – 6.21 (m, 1H), 6.19 – 5.75 (m, 2H), 5.46 – 5.19 (m, 1H), 4.67 – 3.95 (m, 5H), 3.83 – 3.67 (m, 10H), 3.23 – 3.05 (m, 3H), 2.48 – 2.40 (m, 4H), 2.27 – 2.10 (m, 3H), 1.61 – 1.40 (m, 4H), 1.02 – 0.81 (m, 5H).

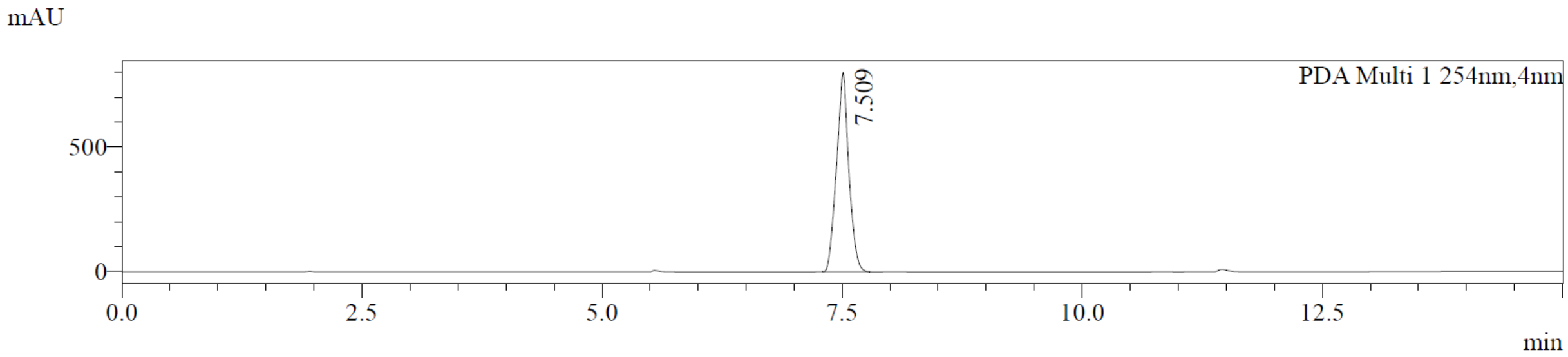

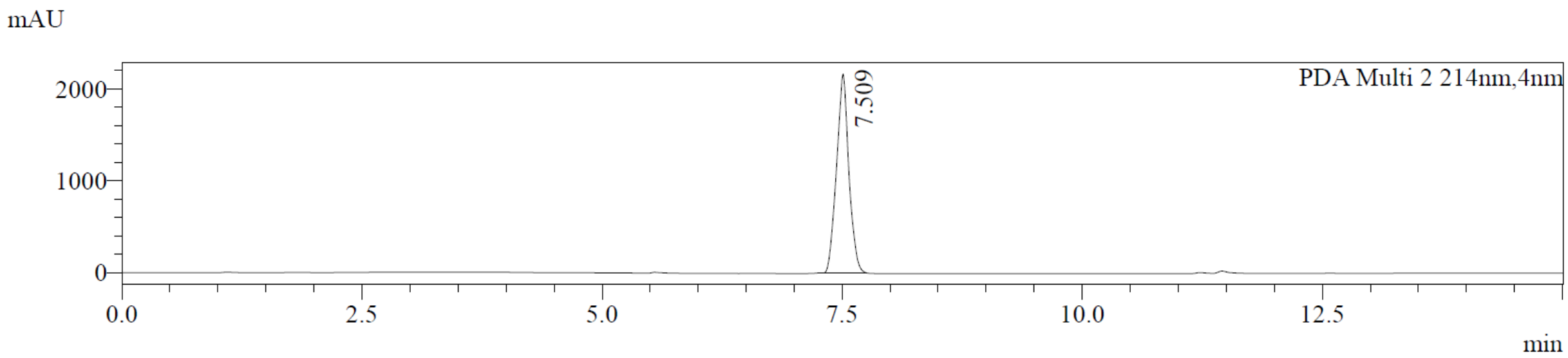

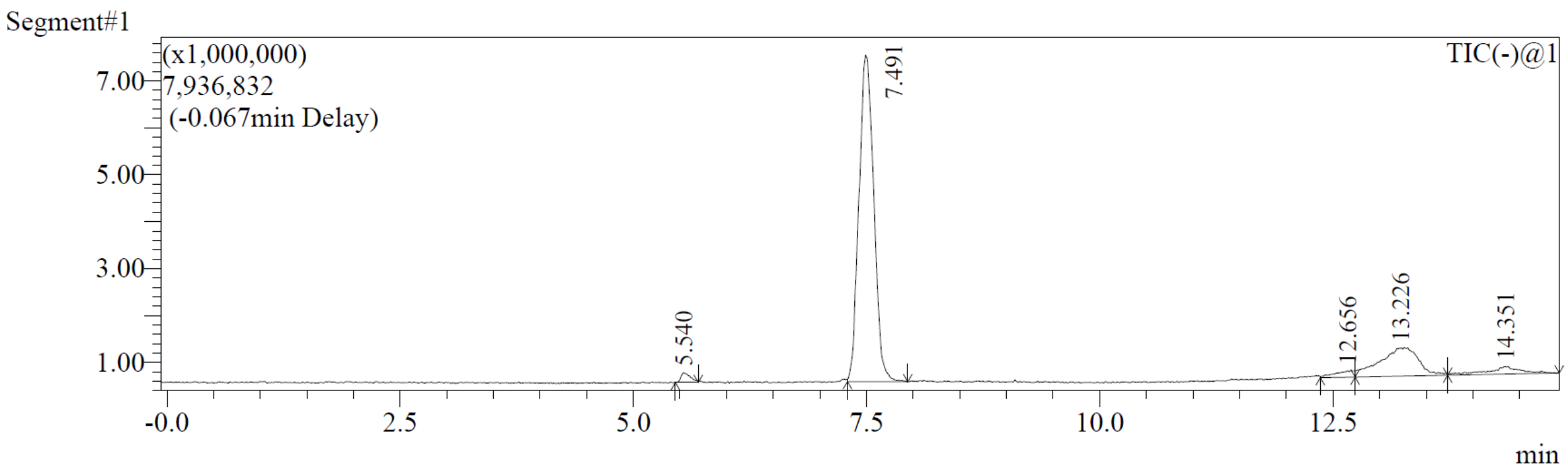

Rt=7.493min Negative

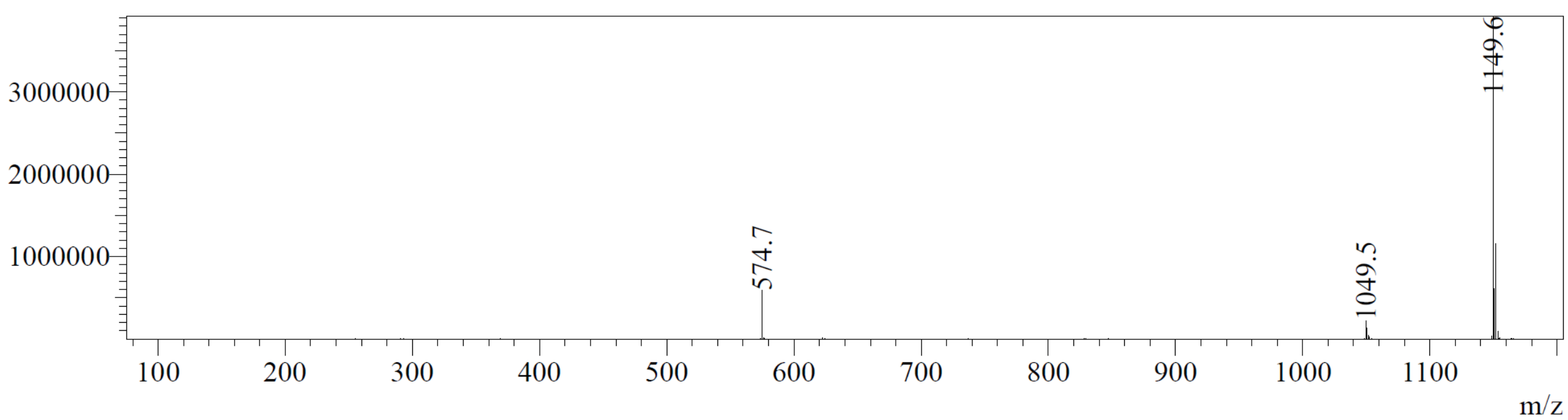

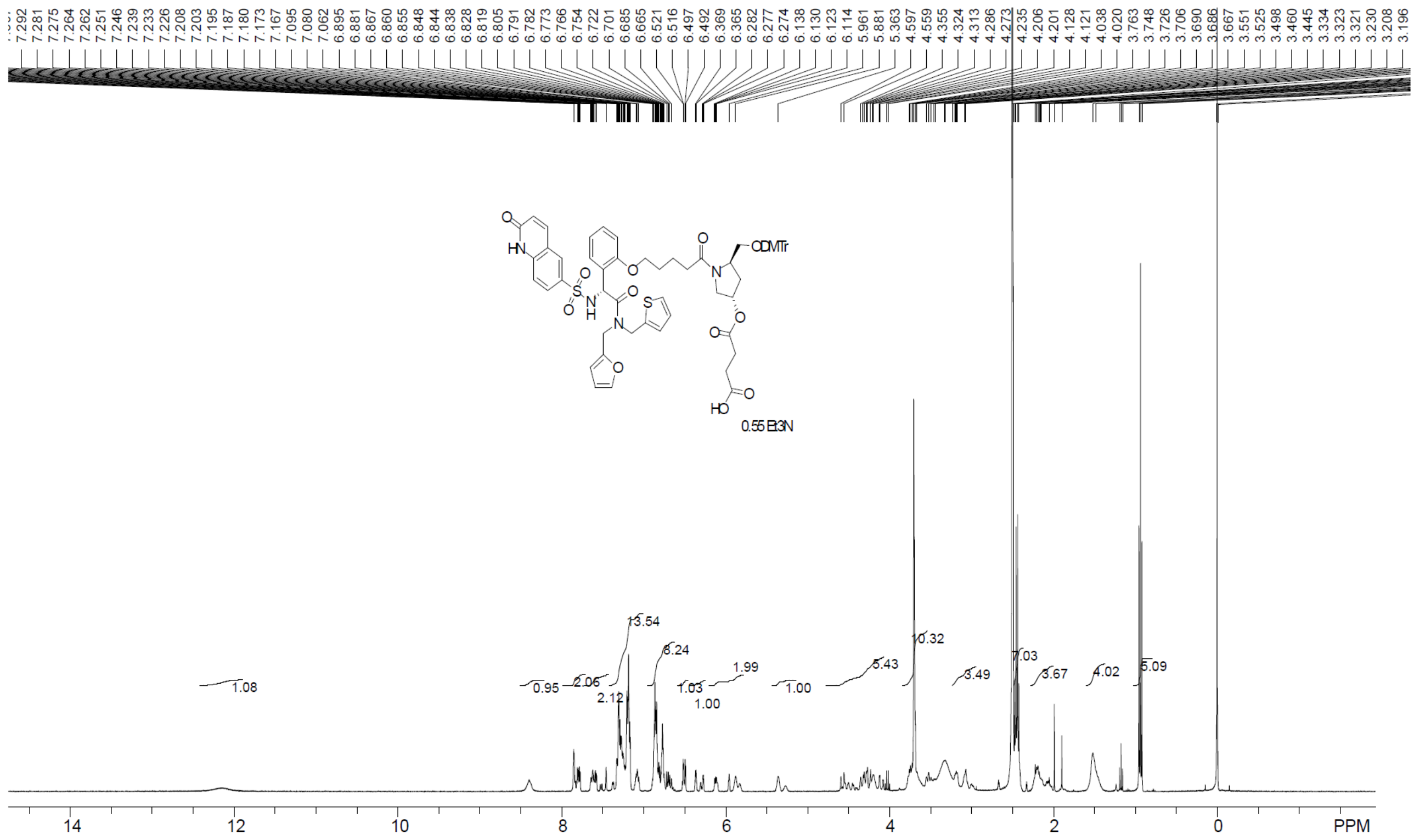

12. Synthesis of AS1411-OSMI-CPG:

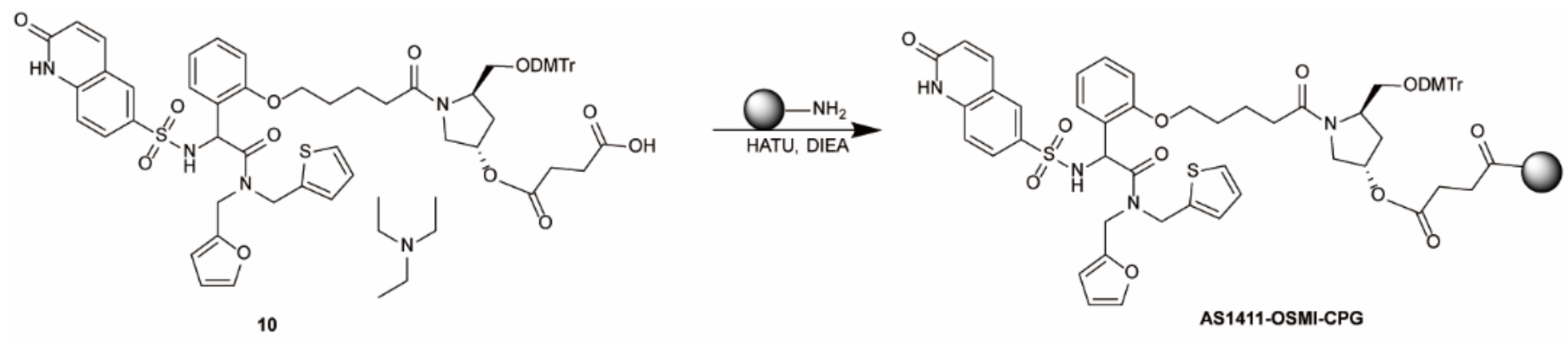

To a solution of **compound 10** (400 mg, 347.41 μmol) in ACN (24.0 mL) were added HATU (160 mg, 420.8 μmol), DIEA (160 μL), lcaa-CPG (1000 Å, 2.0 g) respectively at room temperature and oscillates for 12 hours. After the reaction was completed, CPG was washed with ACN, CAP A (acetic anhydride: tetrahydrofuran= 1:9, v/v, 16.0 mL) and CAP B (N-methylimidazole: pyridine: acetonitrile= 15:10:75, v/v/v, 16.0 mL) was added, the mixture was oscillated at room temperature for 1 hour. Then the mixture was filtered and washed with ACN (100 mL) for 3 times. **AS1411-OSMI-CPG** was obtained after freeze-drying of the mixture as a white power (2.0 g).

13. Synthesis of AS1411-OSMI:

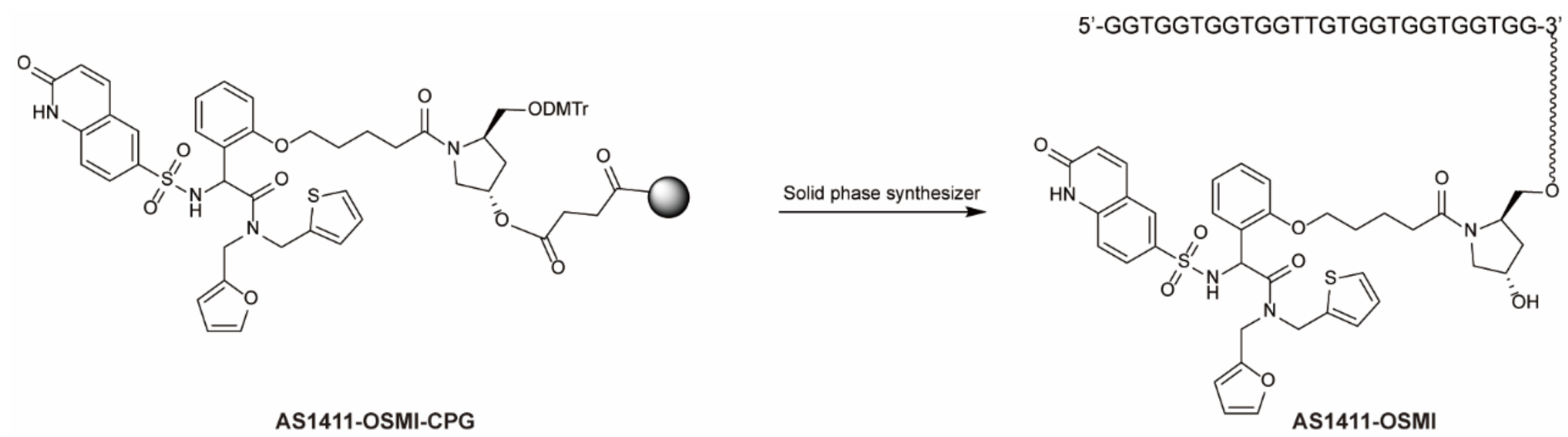

AS1411-OSMI-CPG in synthesis columns (60 mg*32) and synthesize by K-A H-8 Solid phase synthesizer. Solid phase synthesis involves four steps: Detritylation, Coupling, Capping and Oxidation. After reaction was done, CPG of each synthesis column was added 1.5 mL aqueous ammonia and heated in oven at 65 °C for 16 hours. Then collected the supernatant and washed with water (1 mL*3). The combined crude was purified by RP-IP-HPLC (WATERS 2489, 3767) (**Column:** XBridge BET C18 2.5μm. **RP-IP-HPLC method**: Mobile phase A: 2 % HFIP +0.1 % TEA in DD water, Mobile phase B: MeOH) to afford **AS1411-OSMI** as a white power (95.87 mg, purity = 98.792%).

UPLC-MS (WATERS ACQUITY PREMIER): **AS1411-OSMI-UPLC**, *m/z* = 9082.92823 $[M]^-$(deconvolution); t_R = 9.801 min (260 nm). Mass error <50 ppm.

HPLC (SPT-M40): **AS1411-OSMI-HPLC**, t_R = 11.021 min (260 nm), purity: 98.792%.

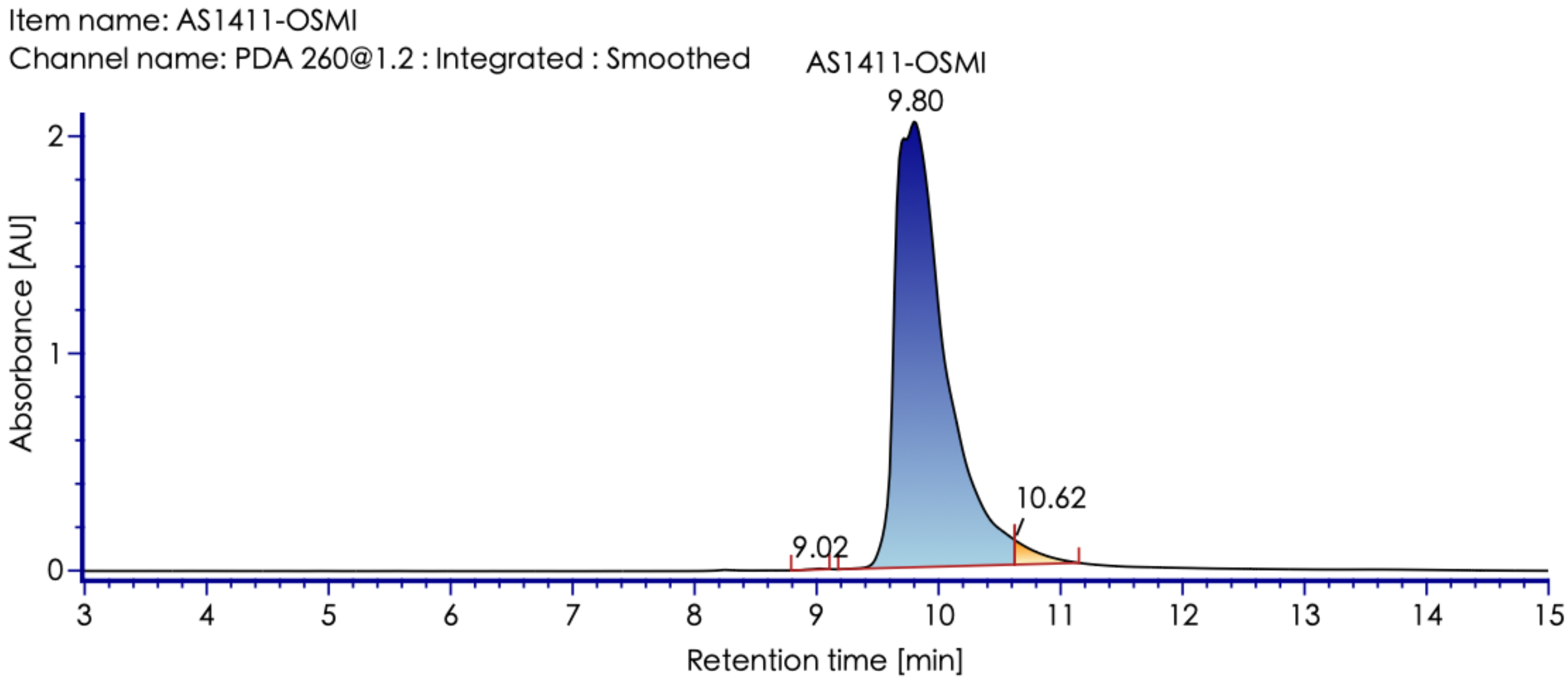

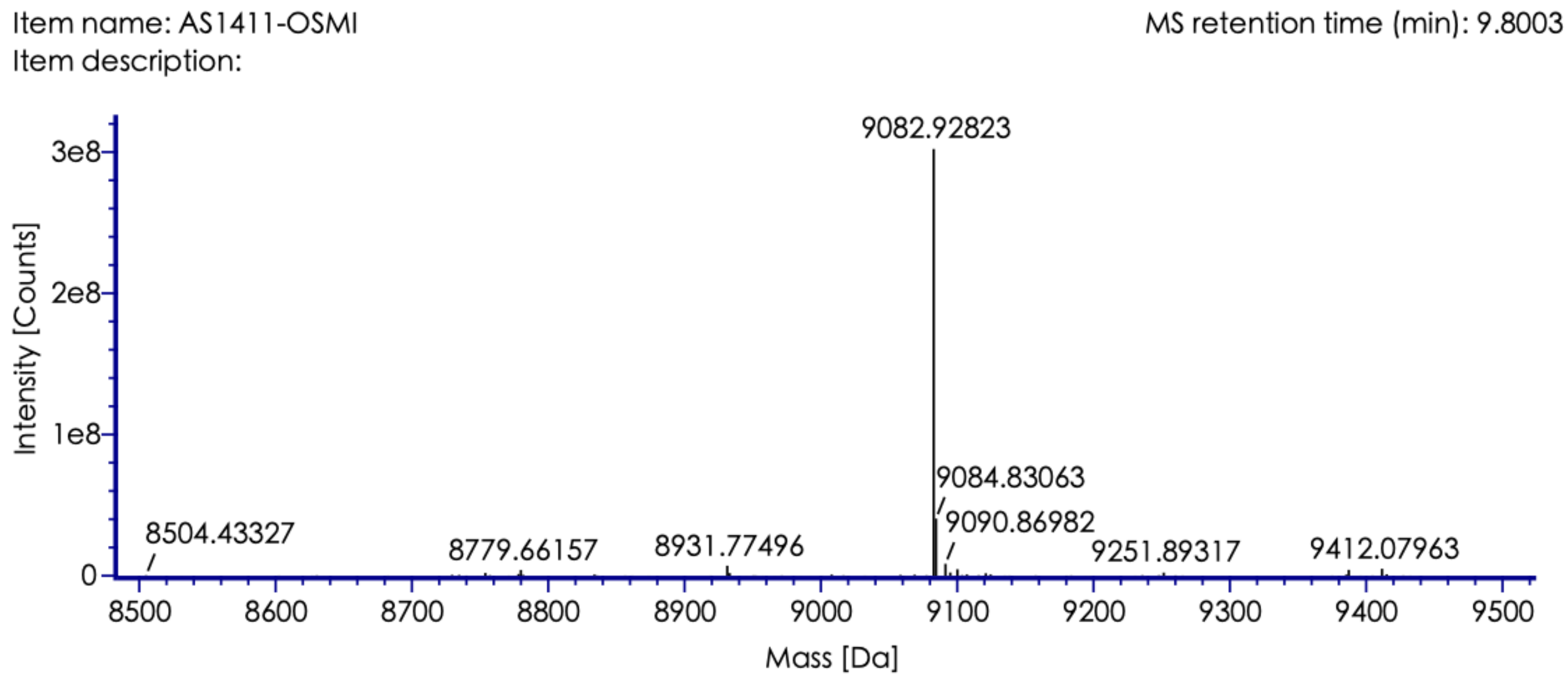

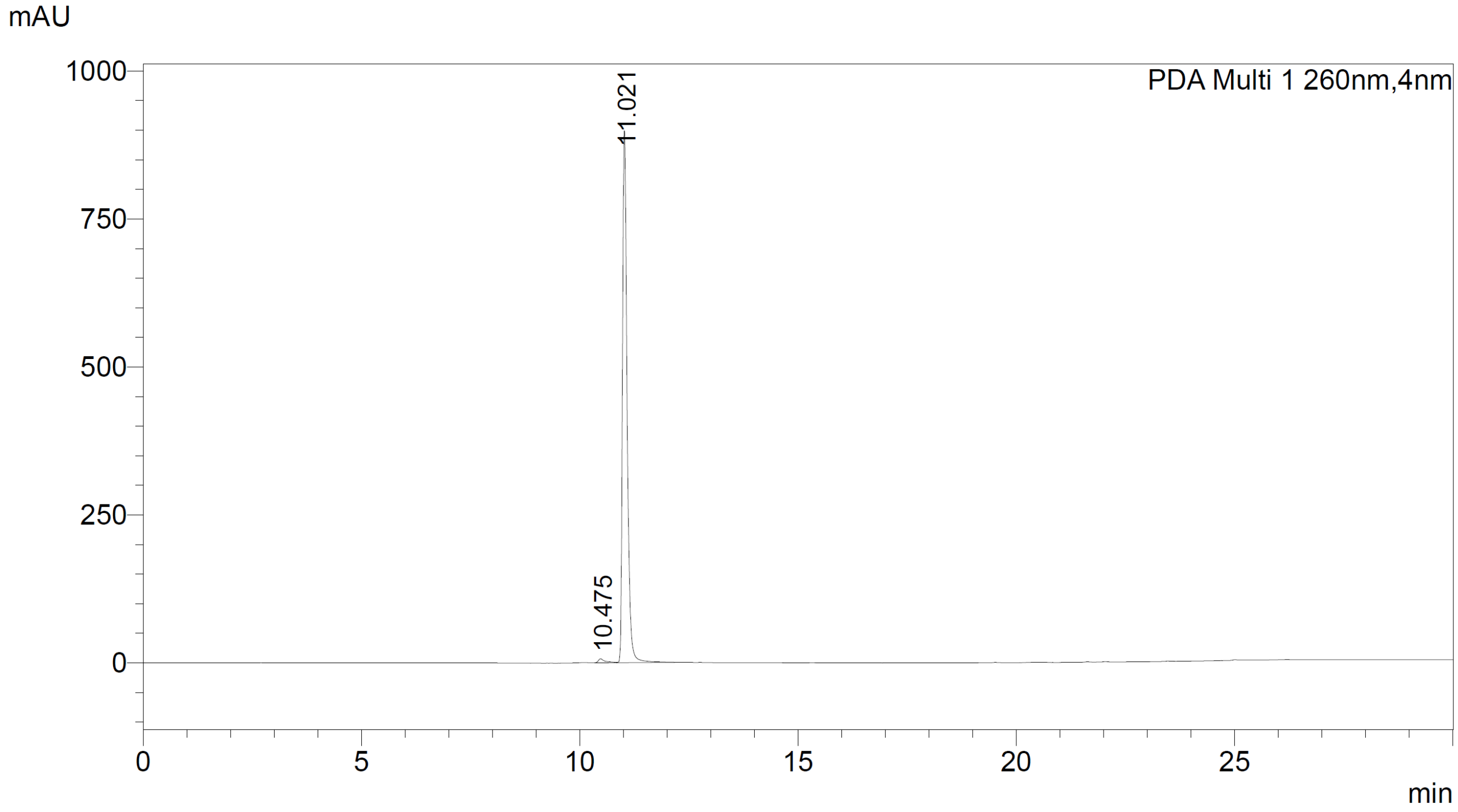
mAU
PDA Multi 1 260nm,4nm
11.021
10.475
1000
750
500
250
0
0
5
10
15
20
25
min
